# Development of a new antigen-based microarray platform for screening and detection of human IgG antibodies against SARS-CoV-2

**DOI:** 10.1038/s41598-022-10823-7

**Published:** 2022-05-16

**Authors:** Sindy Burgold-Voigt, Elke Müller, David Zopf, Stefan Monecke, Sascha D. Braun, Katrin Frankenfeld, Michael Kiehntopf, Sebastian Weis, Thomas Schumacher, Mathias W. Pletz, Ralf Ehricht, The CoNAN Study Group, Thomas Hotz, Petra Enders, Renate Koch, Steffen Mai, Matthias Ullrich, Cora Richert, Cornelius Eibner, Bettina Meinung, Kay Stötzer, Julia Köhler, Michael Kiehntopf, Hans Cipowicz, Christine Pinkwart, Hans Proquitté, Michael Bauer, Petra Dickmann, Annika Licht, Juliane Scholz, Wibke Wetzker, Anita Hartung, Daniel Weiß, Lara Thieme, Gabi Hanf, Clara Schnizer, Jasmin Müller, Jennifer Kosenkow, Franziska Röstel, Joel Guerra, Oliwia Makarewicz, Steffi Kolanos, Juliane Ankert, Stefan Hagel, Christina Bahrs, Nico Andreas, Raphaela Marquardt, Thomas Kamradt, Sabine Baumgart, Stefanie Deinhardt-Emmer, Sebastian Kuhn, Bettina Löffler, Michael Baier, Stefan Glöckner, André Scherag, Mathias W. Pletz

**Affiliations:** 1 Leibniz-Institute of Photonic Technology (Leibniz-IPHT), Jena, Germany; 2 InfectoGnostics Research Campus, Centre for Applied Research, Jena, Germany; 3 Institute of Physical Chemistry, Friedrich Schiller University Jena, Jena, Germany; 4 Institute for Medical Microbiology and Virology, Dresden University Hospital, Dresden, Germany; 5 INTER-ARRAY, Research Center for Medical Technology and Biotechnology (fzmb GmbH), Bad Langensalza, Germany; 6 Institute for Clinical Chemistry and Laboratory Diagnostics and Integrated Biobank Jena (IBBJ), Jena University Hospital - Friedrich Schiller University Jena, Jena, Germany; 7 Institute for Infectious Diseases and Infection Control, Jena University Hospital - Friedrich Schiller University Jena, Jena, Germany; 8 Leibniz-Institute for Infection Biology and Natural Product Research-Hans Knöll Institute - HKI, Jena, Germany; 9 Institut Virion\Serion GmbH, Würzburg, Germany; 10 Technische Universität Ilmenau, Ilmenau, Germany; 11 Head of County Council, Arnstadt, Germany; 12 Public Health Department, Arnstadt, Germany; 13 Family Physician, Großbreitenbach, Germany; 14 Institute of Clinical Chemistry and Laboratory Diagnostics and Integrated Biobank Jena (IBBJ), Jena University Hospital – Friedrich Schiller University Jena, Jena, Germany; 15 Children’s Hospital, Jena University Hospital – Friedrich Schiller University Jena, Jena, Germany; 16 Department of Anesthesiology and Intensive Care Medicine, Jena University Hospital – Friedrich Schiller University Jena, Jena, Germany; 17 Institute for Infectious Disease and Infection Control, Jena University Hospital – Friedrich Schiller University Jena, Jena, Germany; 18 Institute of Immunology, Jena University Hospital – Friedrich Schiller University Jena, Jena, Germany; 19 Institute of Medical Microbiology, Jena University Hospital – Friedrich Schiller University Jena, Jena, Germany; 20 Institute of Medical Statistics, Computer and Data Sciences, Jena University Hospital - Friedrich Schiller University Jena, Jena, Germany

**Keywords:** Infectious diseases, Epidemiology, Immunology

## Abstract

Strategies to contain the current SARS-CoV-2 pandemic rely, beside vaccinations, also on molecular and serological testing. For any kind of assay development, screening for the optimal antigen is essential. Here we describe the verification of a new protein microarray with different commercially available preparations significant antigens of SARS-CoV-2 that can be used for the evaluation of the performance of these antigens in serological assays and for antibody screening in serum samples. Antigens of other pathogens that are addressed by widely used vaccinations were also included. To evaluate the accuracy of 21 different antigens or antigen preparations on the microarray, receiver operating characteristics (ROC) curve analysis using ELISA results as reference were performed. Except for a single concentration, a diagnostic sensitivity of 1 was determined for all antigen preparations. A diagnostic specificity, as well as an area under the curve (AUC) of 1 was obtained for 16 of 21 antigen preparations. For the remaining five, the diagnostic specificity ranged from 0.942 to 0.981 and AUC from 0.974 to 0.999. The optimized assay was subsequently also applied to determine the immune status of previously tested individuals and/or to detect the immunization status after COVID-19 vaccination. Microarray evaluation of the antibody profiles of COVID-19 convalescent and post vaccination sera showed that the IgG response differed between these groups, and that the choice of the test antigen is crucial for the assay performance. Furthermore, the results showed that the immune response is highly individualized, depended on several factors (e.g., age or sex), and was not directly related to the severity of disease. The new protein microarray provides an ideal method for the parallel screening of many different antigens of vaccine-preventable diseases in a single sample and for reliable and meaningful diagnostic tests, as well as for the development of safe and specific vaccines.

## Introduction

The current SARS-CoV-2 pandemic is the third major Betacoronavirus outbreak among humans within the last twenty years: These causative viruses of these outbreaks were SARS-CoV in 2002/2003^1^, MERS-CoV since 2012^2^, and, in the ongoing pandemic, SARS-CoV-2^3^.

The latter originated from South Eastern Asia, and, as it was also the case for SARS- and MERS-CoV, a link to bats has been implicated^3^. So far, four major protein receptors have been characterized that are responsible for binding the various coronaviruses to their host cells^4^. SARS-CoV-2 binds, just like SARS-CoV and certain SARS-related viruses of bats, to angiotensin-converting enzyme 2 (ACE2) receptor^4^ resulting in a variety of pulmonary, vascular, hemostatic and neurological symptoms^5^. High morbidity and mortality rates especially among elderly and patients with comorbidities in a variety of settings ranging from care homes over prisons to dormitories and cruise ships have been observed^6–8^. However, a substantial proportion of infected individuals, especially, of younger, previously healthy individuals, did not presented with clinical symptoms at all, or experienced very mild, uncharacteristic symptoms^9^. This has serious consequences as it complicates outbreak investigations and contact tracing. This, together with the constantly evolving high transmissibility of the virus, led to an explosive spread around the globe with about 360 million cases and 5.6 million deaths within 2 years (https://www.worldometers.info/coronavirus/) and to an inestimably high economic damage. Studies even estimate the worldwide number of excess deaths to be twice or even four times this figure^10^ up to 18.6 million deaths^11^.

Unspecific and diverse clinical presentations make it impossible to identify potentially infective individuals by a simple use of thermal scanners or by screening for infection-associated symptoms. Tests for the detection of SARS-CoV-2/COVID-19 need to fulfill two very different tasks simultaneously. First, they need to be usable for *diagnostics*, i.e., for detecting the causative agent in clinically ill patients, in order to distinguish SARS-CoV-2 infection from other respiratory syndromes. Second, they will be inevitably used, or abused, for *screening* purposes, i.e., in order to find a more or less asymptomatic carrier among a high number of healthy individuals. When thus anticipating a need for serological tests to evaluate antibody levels, the advantages and disadvantages of test principles and platforms need to be discussed. Enzyme-linked Immunosorbent Assays (ELISA) work on serum samples, need laboratory infrastructure and can be automated allowing high throughput testing. Lateral flow (LF) technology can be employed as rapid antigen test to detect a SARS-CoV-2 infection but it also can be used to detect IgG antibodies against the virus in serum or in capillary blood, which is often much easier to obtain. LF assays can be performed virtually anywhere but they are not designed for high throughput applications.

For ELISA as well as for LF-tests, the choice of the test antigen is particularly important^12^ especially given the high rate of mutation and variation of the virus and its antigens. Selection of test antigens can affect the test performance and is one reason, among others, why the performance of the various lateral flow tests and ELISA varies considerably between different sample cohorts and different commercial tests^13^.

Despite all developments in the field of diagnostic tests^14^, an effective, adaptable and safe vaccination is the most crucial tool in curbing Coronavirus disease. The fact that an increasing number of people might have been vaccinated using a wide range of different vaccines, or might be convalescent from possibly undetected Coronavirus disease, there is an increasing need for simple and rapid serological tests that might be used to detect human IgG antibodies against specific molecules of the SARS-CoV-2 virus after vaccination and/or disease, that might distinguish between both, and that might be employed to identify non-responders who need an further injection as well as immunocompromised subjects, people with waning immunity^15^ or with fraudulent documentation on vaccination or recovery.

As microarrays allow discerning reactivities against a multitude of individual antigens and epitopes simultaneously, under identical reaction conditions within a single sample, they allow a more exhaustive characterization of the immunological response than conventional serological tests. They also permit to screen entire panels of antigens simultaneously, saving sample material and lab time. Besides, it is possible to use capillary rather than venous blood or serum samples which makes sample acquisition easier, especially in pediatric, outpatient or community settings.

Against this background, a protein microarray-based assay was developed in this study, which not only serves to determine serum reactivities against various SARS-CoV-2 antigens, but as proof-of-principle, screens in parallel also reactivities against antigens of other clinically relevant vaccine-preventable disease pathogens. Antigens for the detection of antibodies against, *Clostridium tetani*, *Corynebacterium diphtheriae* and the Morbilli virus, were thus included.

The aim of this study is to demonstrate that a new protein microarray platform allows simultaneous screening for the presence or absence of antibodies against different antigens of the SARS-CoV-2 virus and antigens of the above-mentioned pathogens. It also allows a direct comparison of different commercially available antigen preparations. The parallel, cost-effective and time-saving comparison of antigens will, against the background of the high mutation rate of SARS-CoV-2^16^ and its variants^17^, be indispensable in future to keep serological tests and vaccinations up-to-date.

## Results

### Verification of the new SARS-CoV2-VAC antigen microarray

A total of 101 serum samples from the CoNAN study^18^ were tested with the SARS-CoV2-VAC antigen microarray. Details of the study protocol, population and results have been published previously^18^.

The grey value distribution and the individual receiver operating curves (ROC) for each spotted concentration of, at least, 18 different SARS-CoV-2 antigen preparations and the lowest concentrations of the additional vaccination antigens are shown in Supplemental Figs. S1 and S2.

The results of the ROC analyses are summarized in Table 1 where the calculated areas under the curve (AUCs), threshold values, diagnostic sensitivities and specificities are shown for each immobilized antigen concentration. We showed that under the same reaction parameters, spotted and immobilized concentrations of different antigens lead to different performance parameters (Youden Index, AUC, threshold value) with respect to the potential use in a diagnostic test.

**Table 1:** Listed are the antigen manufacturer, antigen graphic and original names, calculated AUCs and Youden indices, threshold values, diagnostic sensitivities and specificities for each immobilized antigen concentration tested in the corresponding buffer.

Antigen manufacturer	Antigen (graphic name)	Antigen (original name)	Concentration (µg/µL)	Max Youden Index	AUC	Threshold (arb. u.)	Diagnostic sensivity	Diagnostic specificity
Biomapper Technology Co., Ltd	Ag_Spike-01_0.1	S2_Spike_BioM_0.1	0.1	1	1	0.326	1	1
	Ag_Spike-01_0.2	S2_Spike_BioM_0.2	0.2	1	1	0.374	1	1
	Ag_Spike-01_0.5	S2_Spike_BioM_0.5	0.5	1	1	0.382	1	1
	Ag_Spike-02_0.1	S2_Spike_BioM_ugp_0.1	0.1	1	1	0.380	1	1
	Ag_Spike-02_0.2	S2_Spike_BioM_ugp_0.2	0.2	1	1	0.391	1	1
	Ag_Spike-02_0.45	S2_Spike_BioM_ugp_0.45	0.45	1	1	0.383	1	1
Medix Biochemica	Ag_Spike-03_0.1	S2_Spike-S1_Med_0.1	0.1	1	1	0.436	1	1
	Ag_Spike-03_0.2	S2_Spike-S1_Med_0.2	0.2	1	1	0.439	1	1
	Ag_Spike-03_0.5	S2_Spike-S1_Med_0.5	0.5	1	1	0.435	1	1
Institut Virion\Serion GmbH	Ag_Spike-04_0.1	S2_Spike-S1-S2_2.23.6.90_VS_0.1	0.1	0.942	0.993	0.059	1	0.942
	Ag_Spike-04_0.2	S2_Spike-S1-S2_2.23.6.90_VS_0.2	0.2	0.981	0.998	0.225	1	0.981
	Ag_Spike-04_0.45	S2_Spike-S1-S2_2.23.6.90_VS_0.45	0.45	1	1	0.615	1	1
	Ag_Spike-05_0.2	S2_Spike-S1-S2_2.23.6.90_VS_ugp_0.2	0.2	1	1	0.587	1	1
	Ag_Spike-05_0.27	S2_Spike-S1-S2_2.23.6.90_VS_ugp_0.27	0.27	1	1	0.628	1	1
Sino Biological Inc	Ag_Spike-06_0.1	S2_Spike-S1_Sino_ugp_0.1	0.1	0.962	0.986	0.394	1	0.962
	Ag_Spike-06_0.2	S2_Spike-S1_Sino_ugp_0.2	0.2	0.962	0.992	0.455	1	0.962
	Ag_Spike-06_0.5	S2_Spike-S1_Sino_ugp_0.5	0.5	0.962	0.992	0.475	1	0.962
Beijing DIAGREAT Biotechnology Co., Ltd	Ag_Spike-07_0.1	S2_S-RBD_DG_0.1	0.1	1	1	0.354	1	1
	Ag_Spike-07_0.2	S2_S-RBD_DG_0.2	0.2	1	1	0.401	1	1
	Ag_Spike-07_0.5	S2_S-RBD_DG_0.5	0.5	1	1	0.397	1	1
Medix Biochemica	Ag_Spike-08_0.1	S2_S-RBD1_Med_0.1	0.1	1	1	0.302	1	1
	Ag_Spike-08_0.2	S2_S-RBD1_Med_0.2	0.2	1	1	0.291	1	1
	Ag_Spike-08_0.5	S2_S-RBD1_Med_0.5	0.5	1	1	0.350	1	1
	Ag_Spike-09_0.1	S2_S-RBD2_Med_0.1	0.1	1	1	0.421	1	1
	Ag_Spike-09_0.2	S2_S-RBD2_Med_0.2	0.2	1	1	0.429	1	1
	Ag_Spike-09_0.5	S2_S-RBD2_Med_0.5	0.5	1	1	0.419	1	1
Institut Virion\Serion GmbH	Ag_nucleocapsid-01_0.1	S2_N_2.23.6.89_VS_0.1	0.1	1	1	0.534	1	1
	Ag_nucleocapsid-01_0.2	S2_N_2.23.6.89_VS_0.2	0.2	1	1	0.598	1	1
	Ag_nucleocapsid-01_0.5	S2_N_2.23.6.89_VS_0.5	0.5	1	1	0.625	1	1
	Ag_nucleocapsid-02_0.1	S2_N_2.23.6.89_VS_ugp_0.1	0.1	1	1	0.616	1	1
	Ag_nucleocapsid-02_0.2	S2_N_2.23.6.89_VS_ugp_0.2	0.2	1	1	0.591	1	1
	Ag_nucleocapsid-02_0.5	S2_N_2.23.6.89_VS_ugp_0.5	0.5	1	1	0.594	1	1
Biomapper Technology Co., Ltd	Ag_nucleocapsid-03_0.1	S2_N_BioM_0.1	0.1	1	1	0.639	1	1
	Ag_nucleocapsid-03_0.2	S2_N_BioM_0.2	0.2	1	1	0.674	1	1
	Ag_nucleocapsid-03_0.5	S2_N_BioM_0.5	0.5	1	1	0.710	1	1
	Ag_nucleocapsid-04_0.1	S2_N_BioM_ugp_0.1	0.1	1	1	0.578	1	1
	Ag_nucleocapsid-04_0.2	S2_N_BioM_ugp_0.2	0.2	1	1	0.574	1	1
	Ag_nucleocapsid-04_0.5	S2_N_BioM_ugp_0.5	0.5	1	1	0.598	1	1
Medix Biochemica	Ag_nucleocapsid-05_0.1	S2_N_Med_0.1	0.1	1	1	0.596	1	1
	Ag_nucleocapsid-05_0.2	S2_N_Med_0.2	0.2	1	1	0.557	1	1
	Ag_nucleocapsid-05_0.5	S2_N_Med_0.5	0.5	1	1	0.552	1	1
University Zürich AG Aguzzi	Ag_nucleocapsid-06_0.1	S2_N_Pr.Ag_ugp_0.1	0.1	1	1	0.309	1	1
	Ag_nucleocapsid-06_0.2	S2_N_Pr.Ag_ugp_0.2	0.2	1	1	0.541	1	1
	Ag_nucleocapsid-06_0.3	S2_N_Pr.Ag_ugp_0.3	0.3	1	1	0.583	1	1
BioVendor	Ag_nucleocapsid-07_0.1	S2_N_Biov_ugp_0.1	0.1	0.962	0.996	0.536	1	0.962
	Ag_nucleocapsid-07_0.2	S2_N_Biov_ugp_0.2	0.2	0.981	0.999	0.629	1	0.981
Beijing DIAGREAT Biotechnology Co., Ltd	Ag_nucleocapsid-08_0.1	S2_N_DG_0.1	0.1	0.980	0.999	0.680	1	0.980
	Ag_nucleocapsid-08_0.2	S2_N_DG_0.2	0.2	1	1	0.673	1	1
	Ag_nucleocapsid-08_0.45	S2_N_DG_0.45	0.45	1	1	0.675	1	1
GeneTex Inc	Ag_nucleocapsid-09_0.1	S2_N_Genet_0.1	0.1	0.865	0.974	0.019	1	0.865
	Ag_nucleocapsid-09_0.2	S2_N_Genet_0.2	0.2	0.875	0.979	0.164	0.913	0.962
	Ag_nucleocapsid-09_0.35	S2_N_Genet_0.35	0.35	0.981	0.986	0.493	1	0.981
Institut Virion\Serion GmbH	Ag_Diphtheria_Toxoid_VS_0.1	Ag_Diphtheria_Toxoid_VS_0.1	0.1	1	1	0.371	1	1
	Ag_Diphtheria_Toxoid_VS_0.5	Ag_Diphtheria_Toxoid_VS_0.5	0.5	1	1	0.387	1	1
	Ag_Measles_Virus_Premium_VS_0.2	Ag_Measles_Virus_Premium_VS_0.2	0.2	1	1	0.037	1	1
	Ag_Measles_Virus_Premium_VS_0.5	Ag_Measles_Virus_Premium_VS_0.5	0.5	1	1	0.111	1	1
	Ag_Tetanus_Toxid_VS_0.1	Ag_Tetanus_Toxid_VS_0.1	0.1	1	1	0.142	1	1
	Ag_Tetanus_Toxid_VS_0.5	Ag_Tetanus_Toxid_VS_0.5	0.5	1	1	0.160	1	1

**Figure 1:**
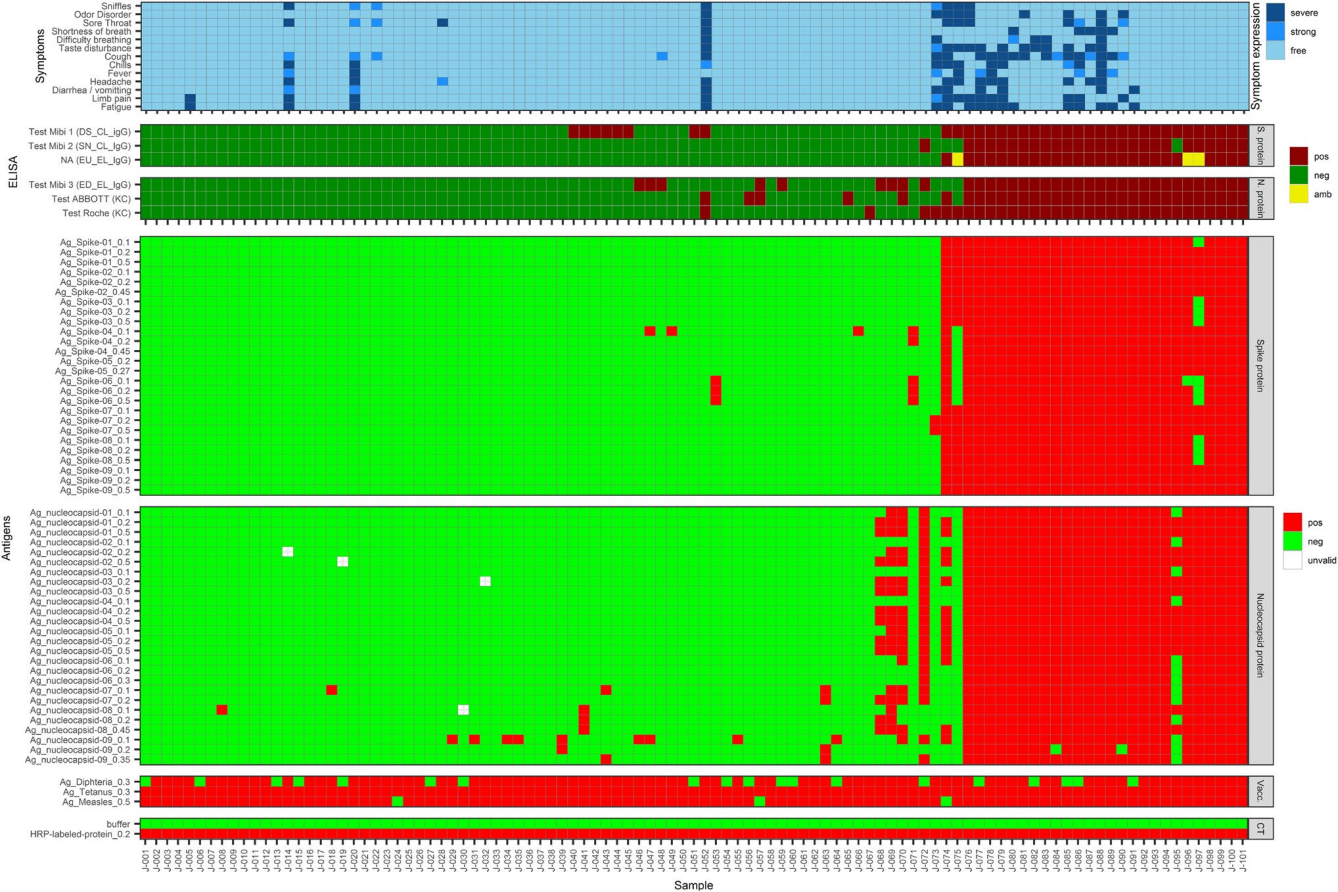
Verification of the new SARS-CoV2-VAC-antigen microarray: 101 ELISA-tested sera from the CoNAN study were tested with a total of 14 antigens spotted at different concentrations and in different buffer conditions.

The SARS-CoV2-VAC antigen microarray results for the 101 CoNAN samples are shown in Fig. 1 (Raw data: Supplemental Fig. S3).

#### Microarray results for test sera with negative results in all six reference ELISAs (group A)

Of all 52 CoNAN samples that were negative in all six commercial ELISA tests, 38 samples (73.1%) were also negative with all antigens on the microarray. For ten samples of this group, reactivities were observed only against the immobilized Nucleoprotein (N) antigen preparations: Ag_nucleocapsid-07, Ag_nucleocapsid-08 and Ag_nucleocapsid-09 at all spotted concentrations. Four samples yielded positive signals with four of the spotted and immobilized Spike (S) antigens (i.e., Ag_Spike-04 at concentrations 0.1 µg/µL and 0.2 µg/µL and Ag_Spike-06 at all concentrations).

#### Microarray results for test sera, with positive results in all six reference ELISAs (group B)

Twenty-three CoNAN samples were positive in all six commercially available ELISAs. Of these, 21 samples (91.3%) were positive for all antigens on the microarray. Only two samples were negative for the Ag_nucleocapsid-09 antigen at a concentration of 0.2 µg/µL. Sixteen of these 23 subjects reported strong to severe symptoms. That is, 30.4% of the patients with positive test results experienced either mild symptoms or remained asymptomatically.

#### Microarray results for test sera, with divergent results of the six reference ELISAs (group C)

The remaining 26 CoNAN samples yielded divergent results with the different ELISAs used, i.e., they yielded positive results in only one to five of these ELISAs. Among them, there were six samples that were positive in the microarray test only for the antigens with AUCs between 0.865 and 0.999 (Ag_Spike-04 and -06, as well as the Ag_nucleocapsid-07, -08 and -09, see above).

One patient (corresponding to sample J-052), reported strong to severe symptoms. However, no Anti-N IgG antibodies could be detected by microarray, despite of four positive ELISAs.

In four sera from this group with inconclusive ELISA results (J-068, J-069, J-070, and J-072), only IgG antibodies against the N protein, but no IgG antibodies against the S protein of SARS-CoV-2 were detected. Since no symptoms were reported by these four donors, it is questionable whether these reactions should be attributed to COVID-19 infections or to cross-reactivity with other agents.

Five sera (J-074, J-075, J-095, J-096, and J-097) were positive with both, S and N antigens. Symptoms were reported by only 2 of 5 subjects.

One last serum (J-073) of this group was particularly interesting: the serum was tested positive only in one ELISA, and it was positive on the microarray only for the antigen Ag_Spike-07 at concentrations 0.2 µg/µL and 0.5 µg/µL. Strong to severe symptoms were reported by the patient, particularly taste disturbance and odor disorder, which are COVID-19 specific.

Antigens Ag_Spike-04 and -06, as well as Ag_nucleocapsid-07, -08 and -09 were excluded from further analyses and array development because of their low specificity.

As a further, important result, it can be stated, that only 20 of 29 seropositive-tested individuals (69%) reported symptoms, whereas 7 of the 72 negative- or cross-reactive individuals (9.7%) did so as well.

### Verification of additional vaccination antigens

**Figure 2:**
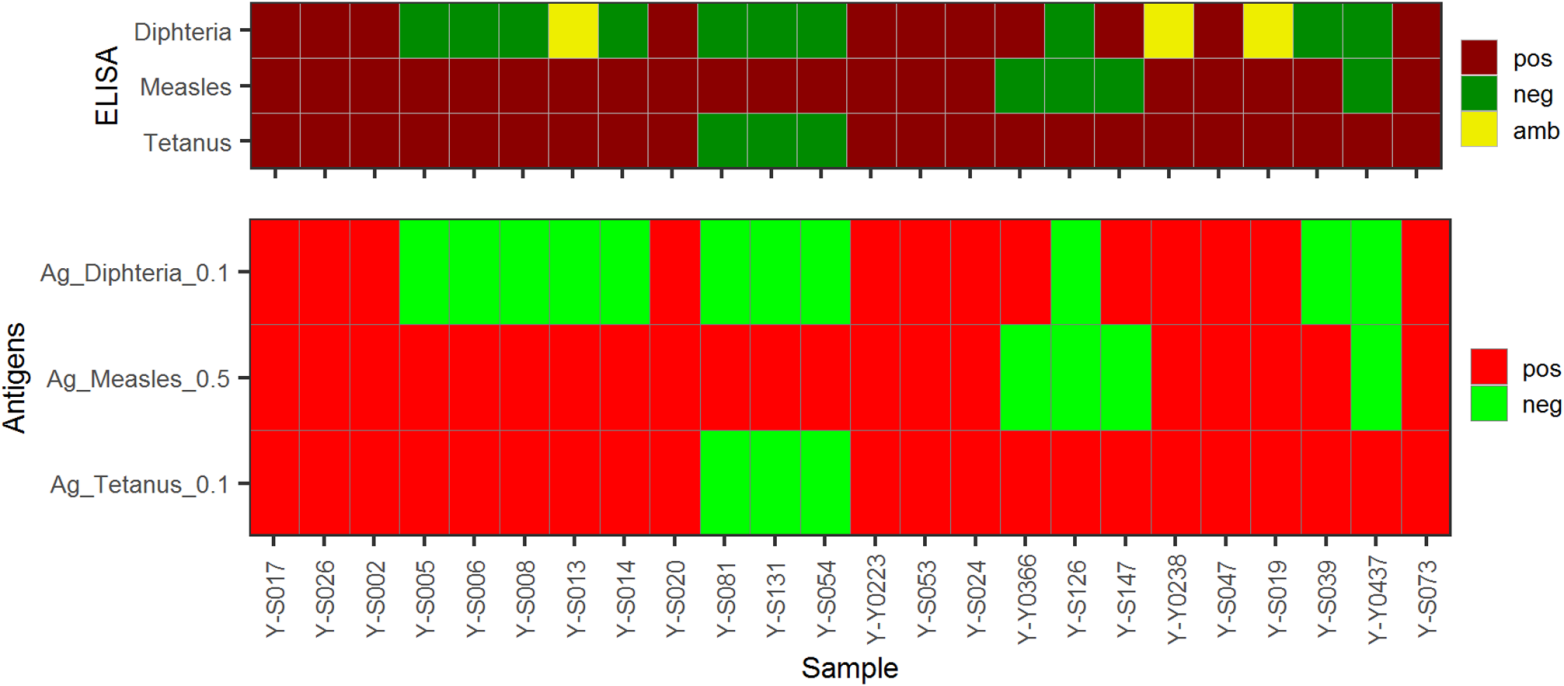
Verification of the additional vaccination antigens on microarray: 24 ELISA-tested sera were tested on the new microarray, 3 typical vaccine antigens were evaluated and used for further analyses.

Twenty-four ELISA-tested serum samples were used to verify the microarray-based assay. The results are shown in Table 1 and Fig. 2 (Raw data: Supplemental Fig. S4).

A parallel analysis for the reactivities towards antigens of *Clostridium tetani* and Morbilli virus for all 24 subjects showed a full concordance between the array and ELISA results.

For the antigen of *Corynebacterium diphtheriae*, array and ELISA results were identical in 21 samples. Three samples yielded ambiguous ELISA results although they were by array clearly negative (Y-S013), or, respectively, positive (Y-Y0238 and Y-S039).

### Application of the optimized SARS-CoV2-VAC antigen assay

The optimized assay was applied to determine the immune status of previously tested individuals. For the above-mentioned reasons, Ag_nucleocapsid-07, Ag_nucleocapsid-08, Ag_nucleocapsid-09, Ag_Spike-04 and Ag_Spike-06 were excluded from the analysis.

**Figure 3:**
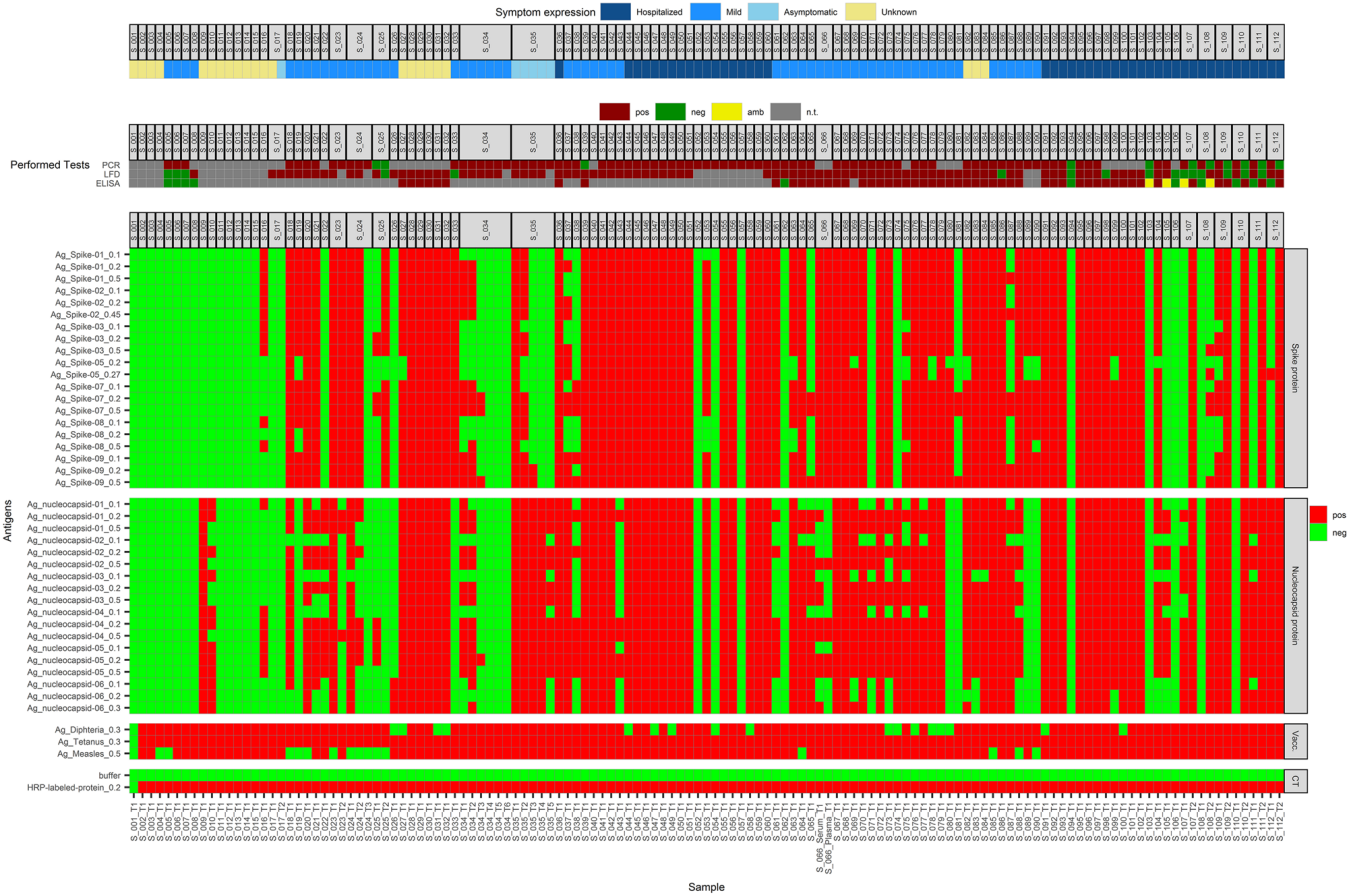
Shown are the results of the new SARS-CoV2-VAC antigen microarray and the available results of real-time (RT)-PCR, lateral flow (LF) and ELISA tests for a total of 112 sera tested, with some subjects tested multiple times, at different time points.

131 sera from a total of 112 subjects were tested, with some of them donating multiple samples at different time points. Each array result is shown in one column in Fig. 3 (Supplemental Fig. S5). In the first three rows, available results of the Real-Time (RT)-PCR, SARS-CoV-2 Nucleoprotein (N) antibody lateral flow (LF) test and ELISAs are listed for comparison. The subsequent rows represent the concentrations of the different antigens.

#### Negative controls and negative samples (group D)

Column 1 in Fig. 3 shows the result for the negative controls without serum; columns 2 to 4 show the results for the pre-2020 negative control sera. These samples were all tested negative for all COVID-19 antigen preparations on the microarray.

Patients who contributed samples S_094, S_103 and S_106 were hospitalized with severe symptoms possibly consistent with COVID-19. All three patients were negative in N antibody LF and ELISA. S_094 and S_103 were also RT-PCR negative while RT-PCR testing was not performed for S_106. All three sera were negative on the array.

#### Sera with positive RT-PCR test as reference (group E)

For 14 subjects, only a positive RT-PCR result, but no SARS-CoV-2N-LF and no ELISA result was available (sera from the follow-up controls and known cross-reactive sera were not included): S_005 to S_007, S_033, S_052 to S_059, S_089 and S_090. For four of these patients, serology by LF and/or ELISA was negative and for ten patients, no serological tests were performed. Of these 14 sera, six were negative and seven were positive for all antigen preparations, except for the Ag_Spike-08 at the spotting concentration of 0.5 µg/µL for serum S_090. For one serum of this group, S_033, which gave a negative result in the LF-test, only antibodies against S antigens were detected with the microarray-based assay. Another conspicuous observation was that three of the six array-negative sera (S_052, S_054 and S_057) originated from patients who were hospitalized because of the severity of their disease.

#### Known cross-reactive sera (group F)

Sera S_009 to S_016 are cross-reactive sera purchased for verification of the new microarray (AbBaltis Ltd, Sittingbourne, UK). The results of the three reference tests were not known for these sera. Based on the array results, it can be determined that N antigens of the SARS-CoV-2 virus have a lower specificity than those of the S protein. For four out of eight of the known-cross-reactive sera in this group (S_011, S_012, S_014 and S_015), no cross-reactivity could be detected for any of the microarray antigens.

#### Sera with positive LF test as reference (group G)

For patients who donated sera S_008, S_017 and S_025 (the last two with follow-ups after 5 months), S_022, S_026 and S_040, positive anti-N antibody LF tests were observed/reported. Two (S_008 and S_017) of these six sera remained completely negative on the microarray, for S_017 also in the follow-up. It can be assumed that the LF-test were false-positive. For two (S_022 and S_026) sera, only anti-N IgG antibodies were detected. S_040 was positive on the microarray with each single antigen. In one sample of this group, serum S_025, anti-N and anti-S IgG antibodies were detected in the first test, but only anti-S antibodies were detected in a follow-up examination after 5 months.

#### Follow-up controls (group H)

In addition to the above-mentioned follow-up controls, the dynamics of post-infection antibody development were tested for three additional subjects at different time points: S_024 three times (sampling interval: 5 months), S_035 five times, and S_034 six times (for the latter two were the intervals of sampling dates between the first three, or four sampling points 4–6 weeks, at the penultimate date three months, and at the last date six months). While donors of S_024 and S_034 showed mild symptoms, the infection for S_035 was asymptomatic although detectable anti-S and anti-N IgG antibodies were produced. At timepoint T1, antibodies against all S and N antigens of the microarray could be detected for serum S_024. At time point T2, N antibodies had already decreased significantly and at time point T3, neither reactivities against S, nor against N antigens were detectable. A comparable time course in antibody decrease was found for sera S_034 and S_035: while the array-pattern was still very similar at times T1 and T2, the positive signals at T3 were already markedly reduced. For S_034, no antibodies could be detected from T4 onwards. For S_035, S antibodies were no longer detectable from T4 on and less N antibodies could already be observed at timepoint T5.

#### Sera with two and three positive reference tests (group I)

For 65 sera, at least two of the three reference tests were positive (serial samples of group H and known cross-reactive sera not included). For 64 sera, the microarray results were also clearly positive. Only in serum S_062, no antibodies were detectable, whereby it must be noted that the ELISA test was also negative. For serum S_019, only anti-S antibodies could be detected, for sera: S_071, S_074, S_080, S_081 and S_105 only anti-N antibodies could be detected. For 32 out of 65 of these sera (49%), reactivities to all antigens were detected.

Subjects 107–112 were hospitalized patients for whom RT-PCR and ELISA testing were performed twice. The first serum sampling time was 2–3 days after the positive RT-PCR, and the 2nd serum sampling was 20–30 days later. ELISA at the first sampling time was negative for four subjects (S_108, S_110, S_111, and S_112), ambiguous for S_107, and already positive for S_109. This ELISA result for S_109 completely coincides with the array result: anti-S and anti-N IgG antibodies were already present at the same time point. In the other five sera, no anti-S IgG antibodies were detectable at T1, in three of five sera, anti-N antibodies could be detected. At time T2, ELISAs were positive for five of six subjects, and the one for sample S_108 was ambiguous. This result was 100% consistent with the results of the array: only for S_108 reactivities were not detectable for all tested S antigens of the microarray. For the other five samples, antibodies against N and S antigens were detected on the microarray.

#### Reactivity towards other antigens

Reactivity to the tetanus toxoid was detected for all 112 subjects, whereas no antibodies to the measles virus antigen were detectable on the microarray for 12 (11%), and to the diphtheria toxoid for 17 (15%) subjects.

### Analysis of post-vaccination sera

**Figure 4:**
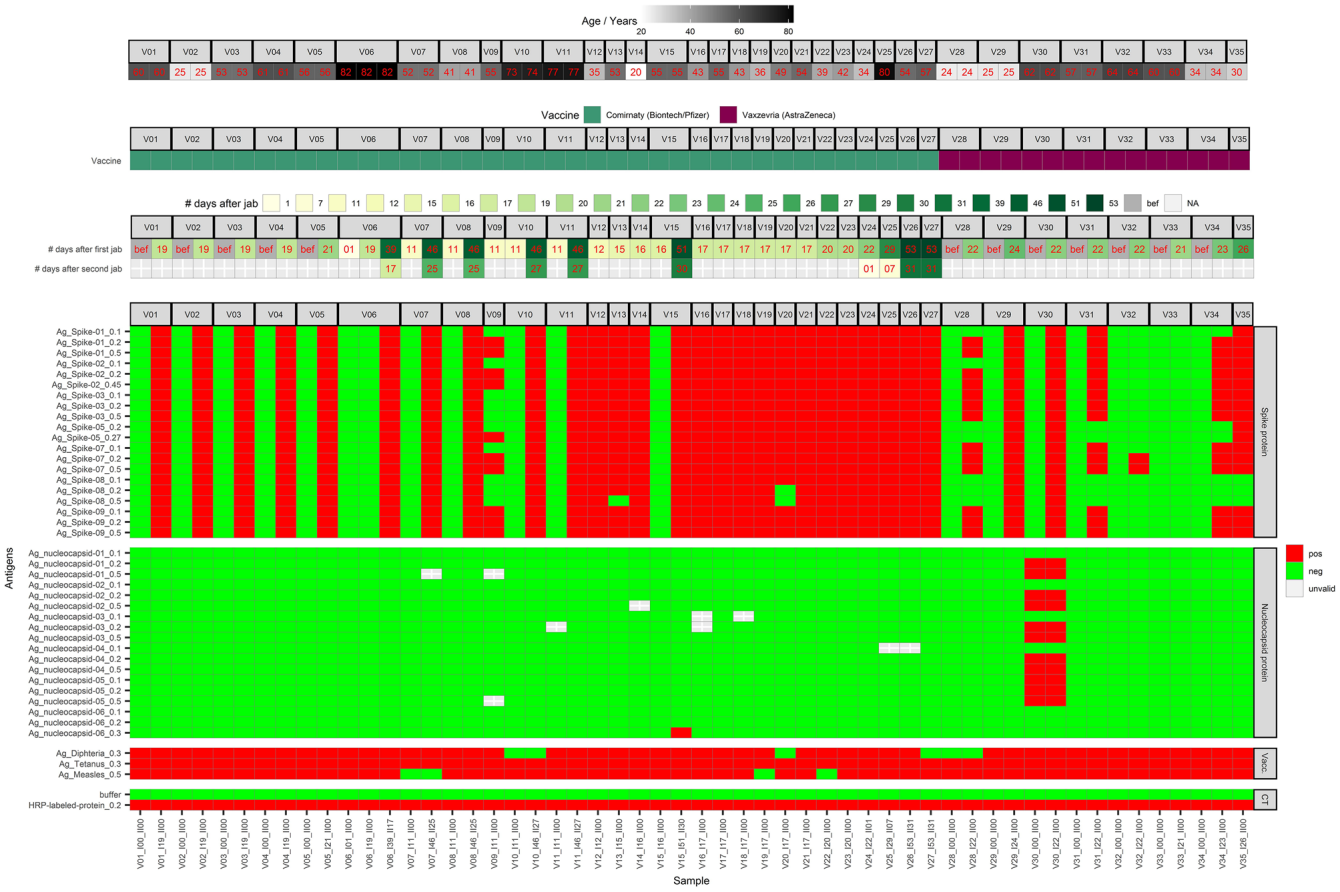
The SARS-CoV2-VAC antigen microarray was used to investigate immunization status after COVID-19 vaccination. Shown are the results of 35 subjects tested who received BioNTech/Pfizer's Cormirnaty vaccine and 8 subjects tested who received AstraZeneca's Vaxzevria vaccine. Sera taken prior vaccination.

The SARS-CoV2-VAC antigen microarray was also used to detect the immunization status after COVID-19 vaccination. The results are shown in Fig. 4 (Raw data: Supplemental Fig. S6).

Out of 35 subjects, 27 (V01–V27) received BioNTech/Pfizer's Comirnaty vaccine (subjects aged 20 to 82 years) and 8 (V28–V35) received AstraZeneca's Vaxzevria vaccine (subjects aged 24 to 64 years).

For 12 subjects (V01–V05, V06, and V28–V34), a serum or a capillary blood sample was taken prior vaccination. For all 12 subjects, no anti-S antibodies could be detected. Subject V30 showed strong cross-reactivity for N antigens in both, the pre- and post-vaccination samples. The only N antigen that did not react in this sample was Ag_nucleocapsid-06 at all spotted concentrations.

#### Sera taken 2 weeks after 1st vaccination

Sampling was performed approximately 14 days after the first vaccination (11–17 days) for 15 subjects (V07–V21). All of them received BioNTech/Pfizer's Comirnaty. For  five subjects (V07, V08, V10, V11 and V15), antibodies could not be detected after this period. All five were tested again four weeks after the 2nd vaccination and were then found to be positive for anti-S antibodies. In sera from another seven subjects, antibodies against all S antigens could be detected. For the remaining three subjects, reactivities with all antigens but one (Ag_Spike-08) were observed.

#### Sera taken 3 weeks after 1st vaccination

16 sera of 35 subjects were tested at approximately 3 weeks after the 1st vaccination. Anti-S antibodies were detected for 14 of 16 subjects (V01–V06, V22–V24, V28–V34), noting that for all BioNTech/Pfizer's Comirnaty vaccinated subjects up to age 61, antibodies against all preparations of S antigens were also detected at all concentrations. For two subjects (V06 and V33), aged 82 and 60 years, no antibodies could be detected after three weeks after the first vaccination. Three weeks after the second vaccination, antibodies against all S antigens were detectable for subject V06. V33 has not been tested again.

For the subjects vaccinated with AstraZeneca's Vaxzevria (V28–V34), antibodies against all tested S antigens could only be detected for two of seven subjects. Most of the other sera, no antibodies directed against antigens Ag_Spike-05 and Ag_Spike-08 were found.

#### Later sampling

One sample (V35, AstraZeneca's Vaxzevria vaccine) was taken 4 weeks after the first vaccination, three samples (V25-V27, BioNTech/Pfizer's Comirnaty) after the 2nd vaccination. For all three BioNTech/Pfizer Comirnaty vaccines, antibodies against all selected S antigens could be detected, whereas for the AstraZeneca's Vaxzevria sample no antibodies could be detected against Ag_Spike-08 even four weeks after the first vaccination.

## Discussion

In a pandemic situation with a new infectious disease, it is—among other issues—enormously important to develop as quickly as possible specific and sensitive diagnostic tools, to adapt these tools to emerging variants of the pathogen^17,19,20^ and, if possible, to develop highly effective vaccines.

The basis for the emergence of the different variants of the SARS-CoV-2 virus is the high mutation rate that single-stranded (ss)RNA viruses accumulate as they replicate^21^. These mutations, individually or in combination, can give the virus a distinct advantage in terms of replication, transmissibility and ability to escape the immune system. For SARS-CoV-2, viral mutation rates and the genetic diversity of virions in a single infected host as a function of viral load are now predictable^22^, but the results published to date in relation with the SARS-CoV-2 infection demonstrate the complexity of characterizing a new virus and drawing conclusions about the course of infection, disease severity and the potential immune response. Thus, even almost 2 years after the beginning of the pandemic, we are still collecting and evaluating data on how strongly or weakly our immune system reacts to a SARS-CoV-2 infection or vaccination, which epitopes are recognized at all and which antibody titers are protective over which period of time^15^. This depends, among other factors, very much on the individual immune response of a person.

For the development of reliable and meaningful diagnostic tests and safe, ever-adapting vaccines, the determination of the optimal antigen under specific reaction conditions is essential. Here, a specific protein microarray technology, with which up to 85 antigens (see “Material and methods”, section: “Microarray production”) or peptides can be screened simultaneously, offers an ideal method.

In addition, it is of great medical importance to distinguish the reactivity patterns following natural infections from those resulting from vaccination containing, e.g., only the spike protein in case of currently used mRNA vaccines. A comparative analysis of sera from patients with different clinical presentations or severities is also important, as is the screening of sera from patients with a known history of other coronavirus infections or exposures for possible cross-reactive antibodies. Complementing the complexity is T-cell immunity, which, as mentioned above, was not analyzed in this work. As described by Andreas et al.^23^, one participant in the CoNAN study^18^ who had PCR-confirmed mild SARS-CoV-2 infection did not develop a measurable antibody response. This result was also confirmed by its testing on the microarray (J-028). Even after vaccination of this patient, serological tests remained negative. However, Interferon gamma (IFNγ) and tumor necrosis factor (TNF) could be detected in respective tests for this subject 6 weeks after infection. This T-cell immunity persisted for months after infection and was enhanced by vaccination^23^. This case report demonstrates that evaluation of prior infection or vaccine response based on antibody detection alone is limited in individual patients and confirms that evaluation of cellular and humoral immune responses is essential to assess overall immunity.

Analyses of ROC curves for the antigens included in the study and subsequent verification revealed insufficient specificities for the antigen preparations Ag_Spike-04 and -06 as well as Ag_nucleocapsid-07, -08 and -09, which precluded their use for further analysis as well as for further development of the SARS-CoV2-VAC antigen microarray.

Furthermore, it could be shown that antigen selection for the detection of convalescent and vaccine IgG response might be different. For the detection of the former, the antigens Ag_Spike-05, -07 and -09 could be identified. Serum J-073 was particularly interesting in this context: it was tested positive in only one of the six ELISAs, and was positive on microarray only for the antigen Ag_Spike-07. The patient reported strong to severe COVID-19 specific symptoms, particularly taste and odor disturbances. Therefore, it is assumed that this patient had COVID-19 and that antigen Ag_Spike-07 is the most sensitive of all antigens tested for detecting convalescence here.

In contrast, it was found that Ag_Spike-05, as well as Ag_Spike-08 are unsuitable for the detection of vaccine-generated antibodies. With regard to the problem of cross-reactivity, it was shown on the basis of the tested, known cross-reactive sera and the identification of cross-reactive sera within our test pool that the spike protein and its receptor binding domains (RBD) are more virus-specific than the N protein. Antigens for which no cross-reactivity was detected are: Ag_Spike-05, Ag_Spike-07 and Ag_Spike-09 as well as the Ag_nucleocapsid-08, at all concentrations tested. This result is due to the fact that the N protein of SARS-CoV-2 has a relatively high degree of homology to the N sequences of the other human coronaviruses, while the S protein is very specific to SARS-CoV-2. These observations demonstrate the criticality of antigen selection and urge caution in the use of poorly performing preparations as test antigens in diagnostic tests.

Another important point could be shown on the basis of the results is the interpretation of missing reactivities despite a positive RT-PCR or despite a vaccination. As the test results for sera S_052, S_054 and S_057 (Fig. 3) and sera V06 and V35 (Fig. 4) show, the time of sampling plays a decisive role: for IgM, seroconversion is reached after a median of 10–13 days after the first symptom or vaccination, for IgG after 12–14 days. Maximum seroconversion is achieved for IgM after 2–3 weeks and for IgG after 3–6 weeks^24,25^. In addition, the possibility of a lack of reactivity due to individual disposition, such as age or pre-existing immunosuppression, should not be disregarded.

Our data for convalescent sera further shows that occasionally, in the context of the individual immune response, only reactivities against a single antigen are detectable. (see samples S_011, S_012, S_014, S_015, S_022, S_026 and S_033, Fig. 3). The fact that both, ELISA assays and IgG antibody LF tests usually use only one antigen to capture serum antibodies contributes to a certain error rate of these tests (as already shown for S_008, S_017 and S_033, Fig. 3). As analyzed for several commercially available tests by Manalac et al.^26^, a parallel use of N and S proteins as capture molecules in antibody LF tests and ELISAs is recommended. This would also allow in most cases to distinguish between post-inoculation and post-infection reactivities. Cases for which this distinction is not possible are those that have been vaccinated with a vaccine made from inactivated virus (e.g. CoronaVac^27^) or have been vaccinated and convalescent.

Since the approval of the first COVID-19 vaccines, there have been legitimate questions if these vaccines will cover emerging strains of the SARS-CoV-2 virus and provide protection to those vaccinated^15^. In addition to those already mentioned above, numerous studies have been conducted on recombination^28^ and mutation rates of the SARS-CoV-2 virus. Vilar et al. have shown^29^ that the proteins the virus consists of mutate at different rates. Particular attention was paid in this study to the main targets of COVID-19 vaccines, therapeutic antibodies and diagnostic tests, i.e., to the spike and nucleocapsid proteins, which exhibit the highest mutational variability^29^. Mutations in the spike proteins at viral domains of host receptor binding are of particular importance, as they could lead not only to an expansion of the host spectrum, but also to changes in transmissibility and possible immune evasion^30^. The currently dominating SARS-CoV-2 Omicron variant, caused more than one million infections in over 100 countries. It also infects people who are fully vaccinated or recovered from infection with previous strains. In contrast to earlier virus variants, a lower mortality rate and generally milder infections have been recorded in South Africa, although the spread of infection is much faster than with the delta variant, as a very high viral load is reached^31^. Because the Omicron variant has been shown to replicate more in the bronchi and not affect lung cells, respiratory distress, oxygen requirements, and hospitalization occur at a very low rate, whereas the Delta variant causes extensive inflammation in the lungs, leading to respiratory distress and often requiring hospitalized oxygen therapy^32,33^. While for the Delta variant no immune escape and neutralization by antibodies was possible as a result of vaccination, Omicron infection, due to 32 mutations of the spike protein near the epitope binding sites for neutralizing or human monoclonal antibodies, results in up to a 40-fold decrease in neutralizing activity. Of particular note is the insertion mutation (ins214EPE). The nucleotide sequence encoding for ins214EPE may indicate recombination after co-infection with seasonal coronavirus, e.g. HCoV 229E^34^. Despite the reduced effect of the neutralizing antibodies, robust T-cell immunity was demonstrated for Omicron, protecting against severe courses. Diagnostically, deletion mutations in both the spike and nucleoprotein result in specificity and sensitivity impairments for both, RT-PCR and rapid antigen assays.

Future monitoring of mutations is essential for the development of effective vaccine variants and reliable serological and molecular tests. In addition to viral genome sequencing, protein microarray technology provides a low-cost, effective and time-saving platform for monitoring mutation rates by allowing reactivity against a variety of antigens to be tested and monitored simultaneously.

## Material and methods

### Sampling

For the verification of SARS-CoV2-VAC antigen microarray, 101 plasma/serum samples from the CoNAN study^18^ were included. This was an exploratory, population-based cohort study in 2020, investigating antibody responses using six different serological assays in a fully RT-PCR-tested community following a large-scale COVID-19 outbreak in the Thuringian village Neustadt am Rennsteig^26^.

Samples were selected out of the total serum panel collected for the CoNAN study (n = 626)^18^, based on the following criteria:

- Positive in PCR, clinical signs, positive in one or more ELISAs.
- Positive in PCR, without or with very weak clinical symptoms, positive or negative in one or more ELISAs.
- Negative sera, negative in PCR (or without PCR test), without clinical signs, negative in ELISAs.
- Sera that were positive in one or more ELISAs although no positive PCR and no clinical symptoms were recorded.

For ROC analysis it was specified to include at least 20 sera with unambiguously positive results, i.e. positive in all six out of six ELISAs and at least 20 sera with unambiguously negative results, i.e. negative in all six out of six ELISAs. (see “Statistical analysis” for details).

The symptoms given in Fig. 1 were recorded within the CoNAN study. Symptoms were considered *severe* if they were greater than 5 on a scale of 1–10 or lasted longer than 7 days. Symptoms were rated as *strong* if they were reported by the subjects but lasted less than 7 days and/or were rated 5 or less. None of the subjects required hospitalization.

Following the verification of the new assay, capillary and venous blood samples were obtained from volunteers with known clinical histories. Venous blood samples were centrifuged at 3000×*g* for 5 min. The plasma supernatants were transferred to screw cap micro tubes (Fisher Scientific, Schwerte, Germany) and stored at − 20 °C for further analysis.

Subjects in this group who had no symptoms were indicated as *asymptomatic*, subjects with symptoms as *mild*, and patients who required hospitalization as *hospitalized* (Fig. 2).

Additional blood samples for this study (usually capillary) were donated by volunteers.

### ELISA analysis

The selected CoNAN samples were ELISA—tested at the Jena University Hospital, Friedrich Schiller University Jena, Germany. All ELISAs used in the study were designed and licensed to detect IgG and IgM antibodies against different antigens of the SARS-CoV-2 virus in routine laboratory praxis^18^. ELISAs from six different manufacturers were used based on convenience and kit availability and served as reference for the array verification.

### Ethics statement, data protection and data management

The study protocol for the CoNAN study was approved by the Medical Ethics Research Committee of the University Hospital Jena, Germany, where the study was conducted. Informed written consent was obtained from each study participant, including all voluntary donors. Confidentiality and personal privacy were respected at all levels of the study. Data associated with the sample were anonymized. All methods used in this study were performed in accordance with the relevant guidelines and regulations of the Medical Ethics Research Committee of the University Hospital Jena, Germany.

### Study design

**Figure 5:**
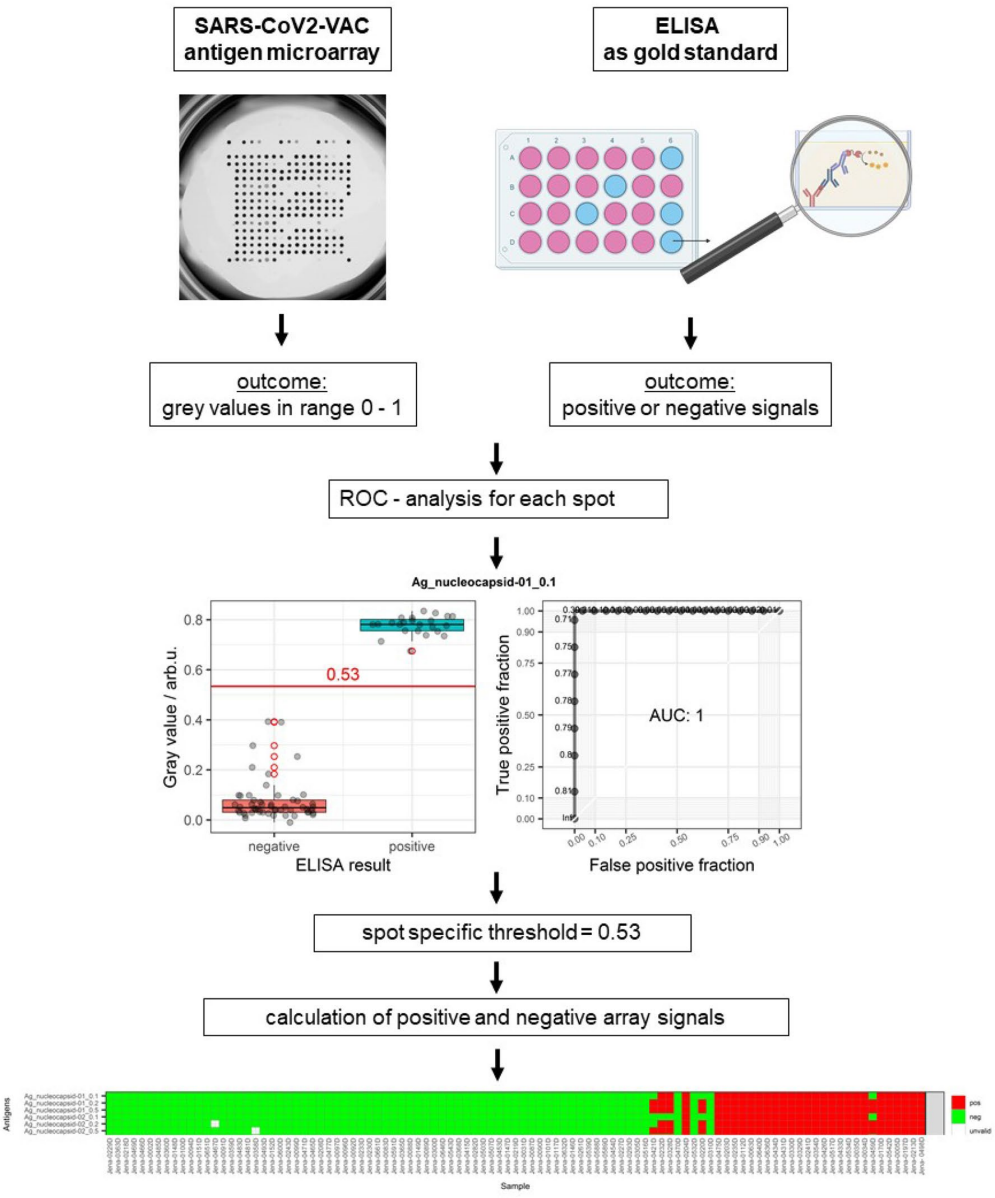
The verification of the new SARS-CoV2-VAC microarray was carried out according to the steps indicated in the scheme.

The verification of the new SARS-CoV2-VAC assay was performed according to the following scheme (Fig. 5).

Using known ELISA results for subjects in the CoNAN study^18^, threshold values for antigens of clinically relevant pathogens in addition to SARS-CoV-2 antigens were established by ROC analysis. The threshold values thus generated were applied to the array data of the known sera. For assay optimization, additional sera from voluntary donors were tested and results were compared to data sets of Real-Time (RT)-PCR, IgG antibody lateral flow (LF) and ELISA.

### Antigen selection and SARS-CoV2-VAC antigen microarray layout

The SARS-CoV2-VAC antigen microarray contained antigens of clinically relevant pathogens in addition to SARS-CoV-2 antigens: SARS-CoV-2 Nucleoprotein N and SARS-CoV-2 Spike S antigens, including S1 and S2 domains.

SARS-CoV-2 nucleocapsid protein (N) is the major structural protein that binds the viral RNA genome. The spike protein (S), a transmembrane protein, consists of two subunits, S1 and S2. S1 mainly contains receptor binding domains (RBD) responsible for cell surface receptor recognition. S2 contains elements necessary for membrane fusion.

The selected antigens were obtained from several manufacturers and were spotted in different concentrations, as presented in Table 2.

**Table 2:** SARS-CoV-2 Antigens, antigen manufacturer and concentrations spotted on the microarray chip**.**

Antigen manufacturer	Antigen	Antigen concentration (µg/µL)
Beijing DIAGREAT Biotechnology Co., Ltd	Nucleocapsid protein	0.45, 0.2, 0.1
	Spike protein	0.5, 0.2, 0.1
Institut Virion\Serion GmbH	Nucleocapsid protein	0.5, 0.2, 0.1 Re-buffered: 0.5, 0.2, 0.1
	Spike protein	0.45, 0.2, 0.1 Re-buffered: 0.27, 0.2
Biomapper Technology Co., Ltd	Nucleocapsid protein	0.5, 0.2, 0.1 Re-buffered: 0.5, 0.2, 0.1
	Spike protein	0.5, 0.2, 0.1 Re-buffered: 0.45, 0.2, 0.1
University Zürich AG Aguzzi	Nucleocapsid protein	0.3, 0.2, 0.1
Sino Biological Inc	Spike protein (subunit 1)	0.5, 0.2, 0.1
BioVendor	Nucleocapsid protein	0.2, 0.1
GeneTex Inc	Nucleocapsid protein	0.35, 0.2, 0.1
Medix Biochemica	Nucleocapsid protein	0.5, 0.2, 0.1
	Spike protein (subunit 1)	0.5, 0.2, 0.1
	Spike protein (RBD1)	0.5, 0.2, 0.1
	Spike protein (RBD2)	0.5, 0.2, 0.1

To simplify graphical mapping, SARS-CoV-2 antigen preparations were named with sequential numbers (Table 1).

Each SARS-CoV-2 antigen was spotted in up to three concentrations by adding a spotting buffer to the antigen prior to the spotting process. For most antigens, 0.5 µg/µL was used as highest concentration, unless the original preparation was provided by the manufacturer at a lower concentration. Some antigens were re-buffered in order to remove potentially interfering additives. The Sino RBD-Protein antigen (spots 29–31, Supplemental Fig. S7) was excluded from further testing because of false positive signals in negative controls without serum during early testing.

In addition, the new SARS-CoV2-VAC antigen microarray contained a group of typical vaccine antigens, i.e. the antigens from *Clostridium tetani*, *Corynebacterium diphtheriae* and the Morbilli virus (Table 3).

**Table 3:** Vaccine antigens and peptides spotted on the microarray chip.

Antigen manufacturer	Antigen	Antigen concentration (µg/µL)
Institut Virion\Serion GmbH	Diphtheria toxoid	0.3
	Tetanus toxoid	0.3
	Measles virus high spec (premium)	0.5
	Mumps Virus	0.5
	Human orthopneumovirus (RSV)	0.5
	Influenza A virus	0.2
peptides & elephants GmbH	Human polio virus (HPV)	0.5
	Chlamydia trachomatis (Ctr)	0.5

In addition, the new array also contained peptides that were not evaluated in this study. Different concentrations of human IgG and human IgM were spotted as hybridization controls. An HRP (horseradish peroxidase)—labeled protein was spotted as a positive control, to confirm substrate addition, and a buffer was spotted as a negative control.

Each antigen concentration was spotted in triplicates. All replicated spots were evenly distributed across the microarray. The complete layout of the microarray is shown in Supplemental Fig. S7.

### Microarray production

The microarrays used in this study were manufactured by INTER-ARRAY (fzmb GmbH, Research Centre for Medical Technology and Biotechnology, Bad Langensalza, Germany).

All antigens were covalently immobilized directly onto the epoxy-coated plastic surface in the bottom of eight-well microtiter strips (see Supplemental Fig. S7). The spotted area is 3.2 mm by 3.2 mm. The microarray accommodates 289 spots with an average diameter of approximately 120 µm.

After manufacturing, each single 8-well strip was sealed under argon atmosphere and stored at 4 °C until use.

### Microarray test procedures

Antibody detection using these protein microarrays was performed to the following previously optimized protocol. The microarrays were first incubated twice with washing buffer (1xPBS/0.05%; Tween 20/0.25%; Triton X 100) at 37 °C and 400 rpm for 3 min. Afterwards, protein arrays were incubated with blocking buffer (1xPBS/0.05%; Tween 20/0.25%; Triton X 100 and 2% milk powder) at 37 °C and 300 rpm for 5 min in order to block unspecific binding sides. Subsequently diluted serum samples (1:100, in buffer) were incubated for 30 min at 37 °C and 300 rpm. After a washing step as described above (37 °C, 400 rpm, 5 min), the protein microarrays were incubated with a diluted (1:1000) HRP-coupled anti-human IgG antibody (Sigma-Aldrich, Steinheim, Germany) at 37 °C and 300 rpm for 30 min. Then the protein microarrays were washed twice with washing buffer (37 °C, 400 rpm, 3 min). Finally, the microarrays were incubated with the substrate SeramunGrün chip (Seramun Diagnostica GmbH, Heidesee, Germany) for exactly 10 min at 25 °C, without shaking.

### Microarray image analysis

The protein microarrays were read out by the ArrayMate device and data were analyzed using IconoClust software according to the manufacturer’s specifications (both by Abbott Rapid Diagnostics Jena GmbH, Jena, Germany).

Relative signal intensities of defined regions (at predefined spot coordinates) on the microarray were determined during read out. The normalized intensities (NI) of the spots were determined as NI = 1 − (M/BG), where M is the average intensity of the spot and BG is the intensity of the local background. Hence, NI values ranged between 0 (no signal) and 1 (maximal intensity).

### Statistical analysis

The analysis of the determined grey value signals of each individual antigen was performed by an in-house-developed script in the programming language R (version 4.0.2)^35^.

The receiver operator characteristics (ROC) curve analysis were created using the *pROC* package (version 1.16.2). For the SARS-CoV-2 antigens, the ELISA results of the CoNAN study served as reference. Patient samples were classified as “positive” only if all six separate ELISA results of the CoNAN study were positive, and classified “negative” only if all six ELISA results were negative. Patient samples exhibiting mixed ELISA test results were excluded from ROC analysis. The analysis using *Clostridium tetani*, *Corynebacterium diphtheriae* and Morbilli virus antigens was more straightforward using positive/negative ELISA tested reference sera (Institut Virion\Serion GmbH, Würzburg, Germany) for ROC analyses. It should be noted that the ROC analysis of the spotted *Clostridium tetani* and *Corynebacterium diphtheriae* antigens was performed with microarrays containing a three times lower concentration of tetanus and diphtheria antigens (Ag_Tetanus_0.1 and Ag_Diphtheria_0.1*)* than the microarrays used for further analyses (Ag_Tetanus_0.3 and Ag_Diphtheria_0.3).

The area under the curve (AUC), diagnostic sensitivity, and specificity for each antigen were calculated to evaluate the accuracy of the individual antigens on the SARS-CoV2 VAC-antigen microarray. According to Gardener 2006^36^, an AUC of 0.5 to 0.7 is interpreted as less accurate, 0.7 to 0.9 as moderately accurate, and 0.9 to 1 as highly accurate. The perfect discrimination is achieved if the AUC for an antigen had a value of 1.0^37^.

The cut-off values of each antigen were set to the maximum Youden-Index^38^. The signal thresholds thus determined were applied to the corresponding antigen grey levels of the different samples discriminating positive and negative test results. All grey values greater than the corresponding threshold were considered positive. Grey values that were less than or equal to the signal threshold were classified as negative. The cut-off values of the positive and negative controls, HRP-labeled-protein_0.2 and buffer*,* were computed employing the CoNAN study data. First, the median values of the grey values of the *HRP-labeled-protein_0.2* (*median_HRP*) and buffer (*median_buffer*) were calculated across all CoNAN samples. The threshold for the positive and negative controls was then defined as *median buffer* value plus 50% of the difference between *median_HRP* and *median_buffer* (*median_buffer* + *0.5 (median_HRP − median_buffer)*).

Data visualization was performed by the *plotROC* package (version 2.2.1) and *ggplot2* package (version 3.3.2).

## Supplementary Information

Supplementary Information.

## Data Availability

All datasets generated and analyzed during the current study are provided within the manuscript, or as Supplemental Files and are available from the corresponding author on reasonable request.
