# Supplementary material for: Development of a new antigen-based microarray platform for screening and detection of human IgG antibodies against SARS-CoV-2
Source: Sci Rep. 2022 May 16;12:8067. doi: 10.1038/s41598-022-10823-7 (PMC9109672; doi:10.1038/s41598-022-10823-7)
Supplement: Supplementary file 1 — Supplementary Information. [file 41598_2022_10823_MOESM1_ESM.zip › Supplemental_File/Figure_S1.pdf]

Ag_Spike−01_0.1

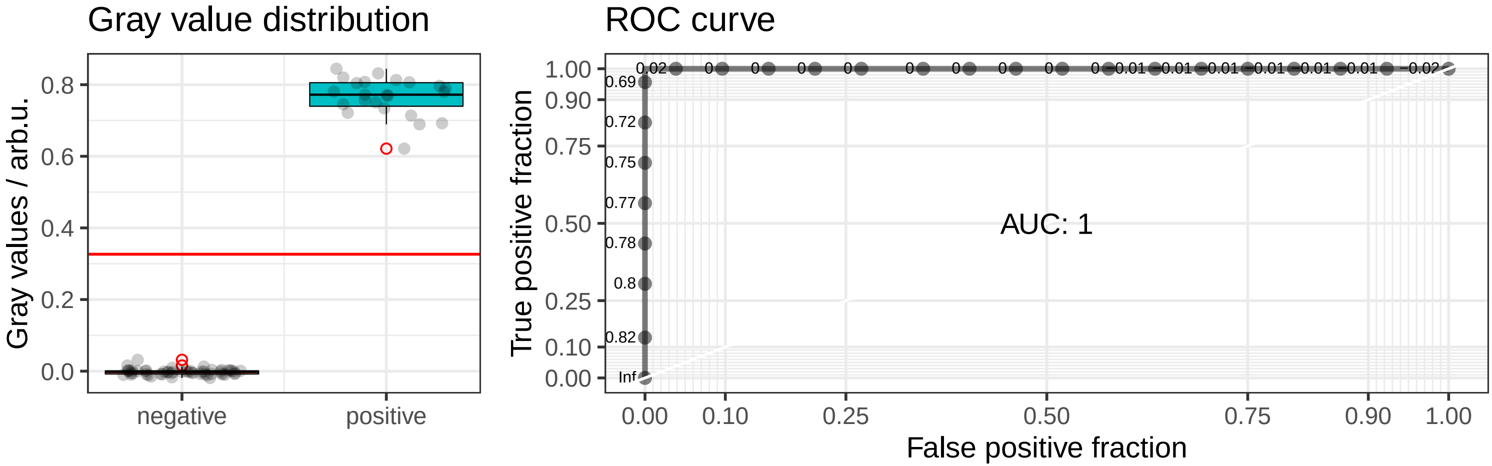

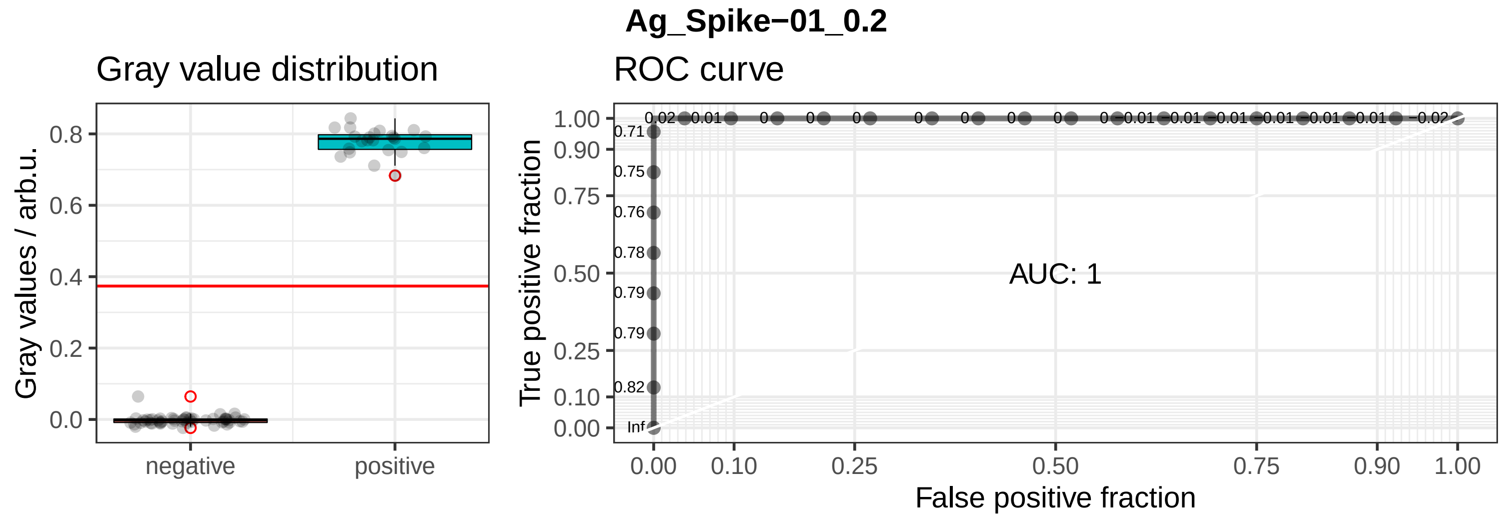

Ag_Spike−01_0.2
Gray value distribution
Gray values / arb.u.
0.0
0.2
0.4
0.6
0.8
negative
positive
ROC curve
True positive fraction
False positive fraction
0.00
0.10
0.25
0.50
0.75
0.90
1.00
AUC: 1
Inf
0.82
0.79
0.79
0.78
0.76
0.75
0.71
0.02
0.01
0
−0.01
−0.02

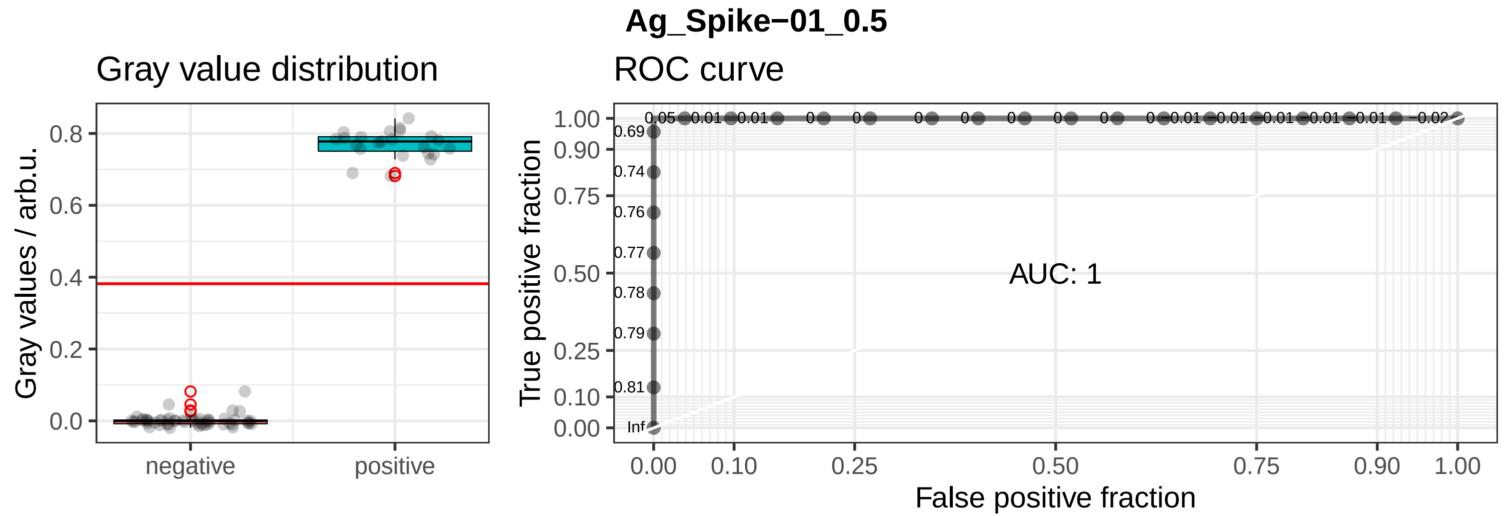
Ag_Spike−01_0.5
Gray value distribution
Gray values / arb.u.
0.0
0.2
0.4
0.6
0.8
negative
positive
ROC curve
True positive fraction
0.00
0.10
0.25
0.50
0.75
0.90
1.00
False positive fraction
AUC: 1
Inf
0.81
0.79
0.78
0.77
0.76
0.74
0.69
0.05
0.01
0
−0.01
−0.02

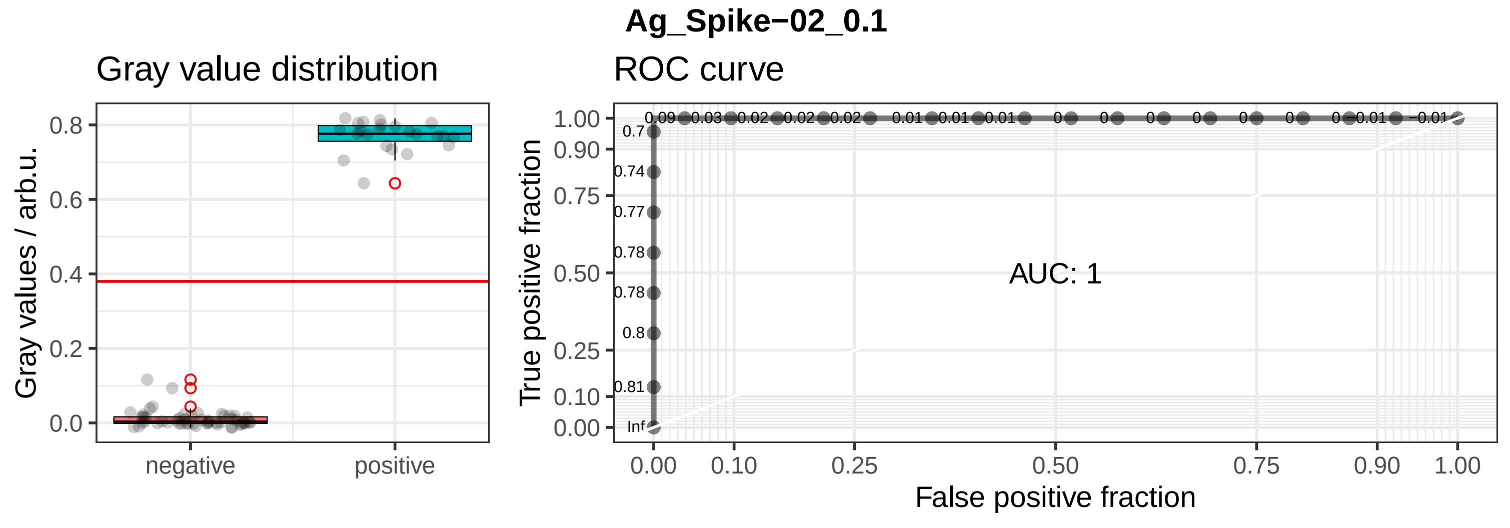
Ag_Spike−02_0.1
Gray value distribution
Gray values / arb.u.
0.8
0.6
0.4
0.2
0.0
negative
positive
ROC curve
True positive fraction
False positive fraction
1.00
0.90
0.75
0.50
0.25
0.10
0.00
0.00
0.10
0.25
0.50
0.75
0.90
1.00
AUC: 1
Inf
0.81
0.8
0.78
0.78
0.77
0.74
0.7
0.09
0.03
0.02
0.02
0.02
0.01
0.01
0.01
0
0
0
0
0
0
0
−0.01
−0.01

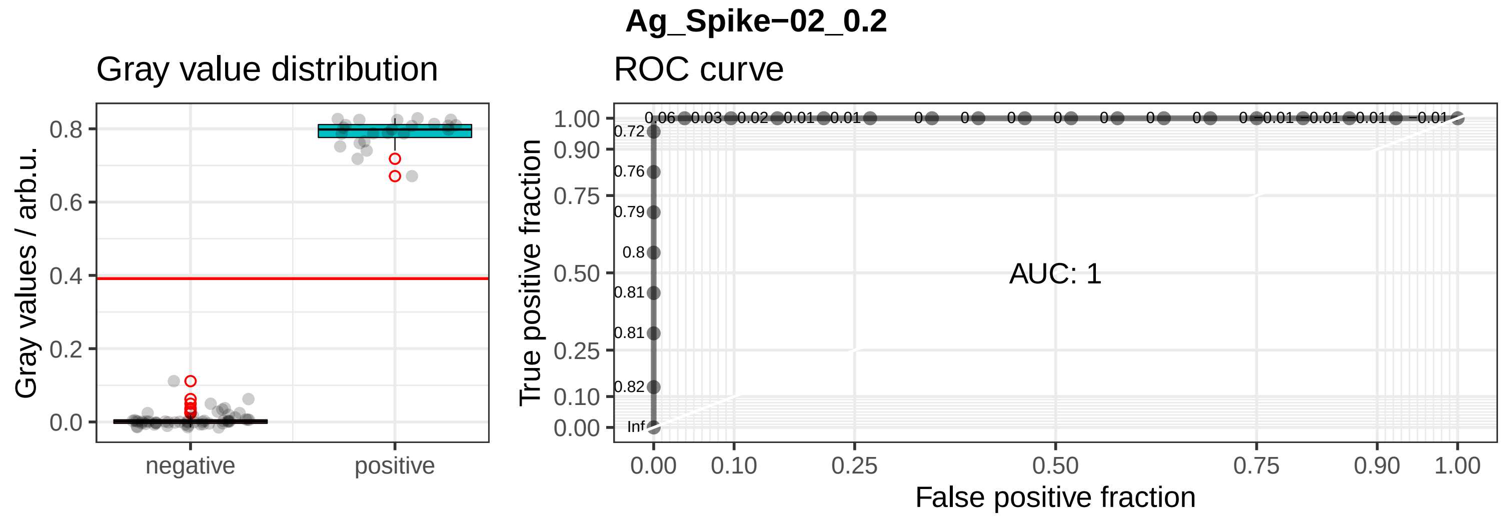
Ag_Spike−02_0.2
Gray value distribution
Gray values / arb.u.
negative
positive
ROC curve
True positive fraction
False positive fraction
AUC: 1
Inf
0.82
0.81
0.81
0.8
0.79
0.76
0.72
0.06
0.03
0.02
0.01
0.01
−0.01

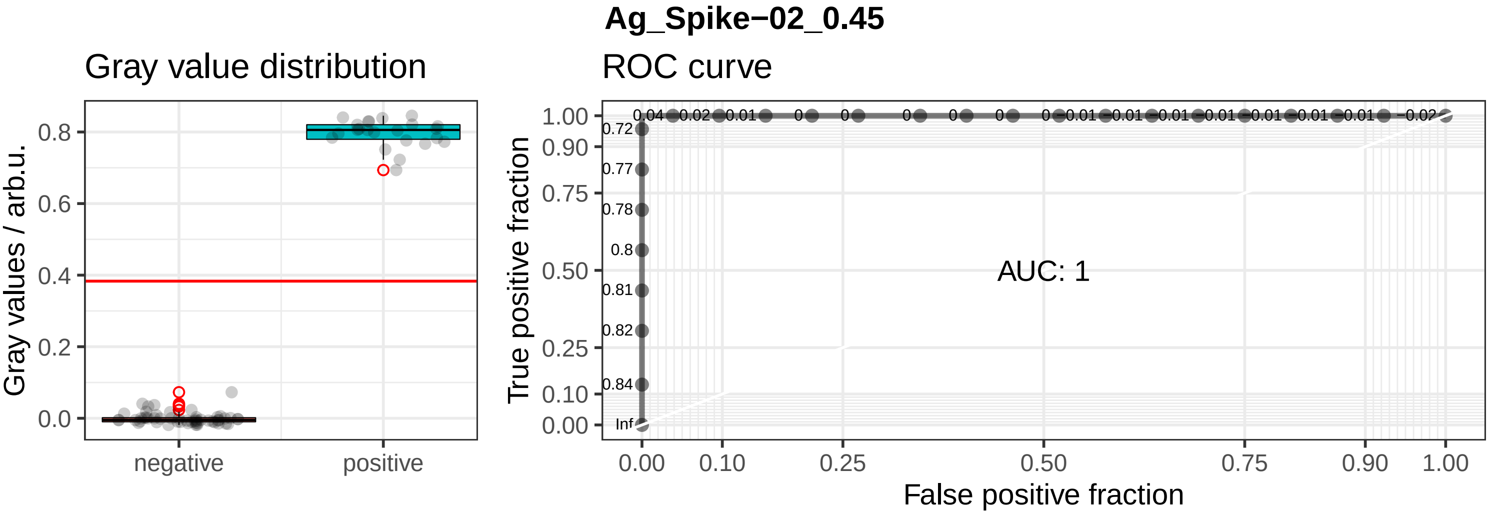
Ag_Spike−02_0.45
Gray value distribution
Gray values / arb.u.
0.8
0.6
0.4
0.2
0.0
negative
positive
ROC curve
True positive fraction
False positive fraction
1.00
0.90
0.75
0.50
0.25
0.10
0.00
0.00
0.10
0.25
0.50
0.75
0.90
1.00
AUC: 1

Ag_Spike−03_0.1

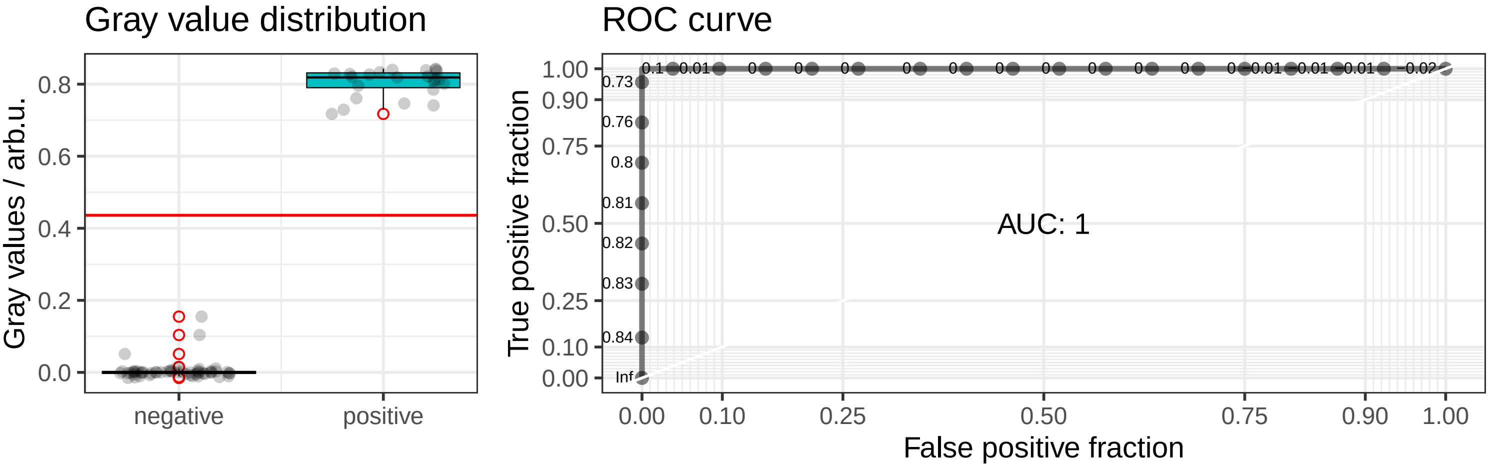

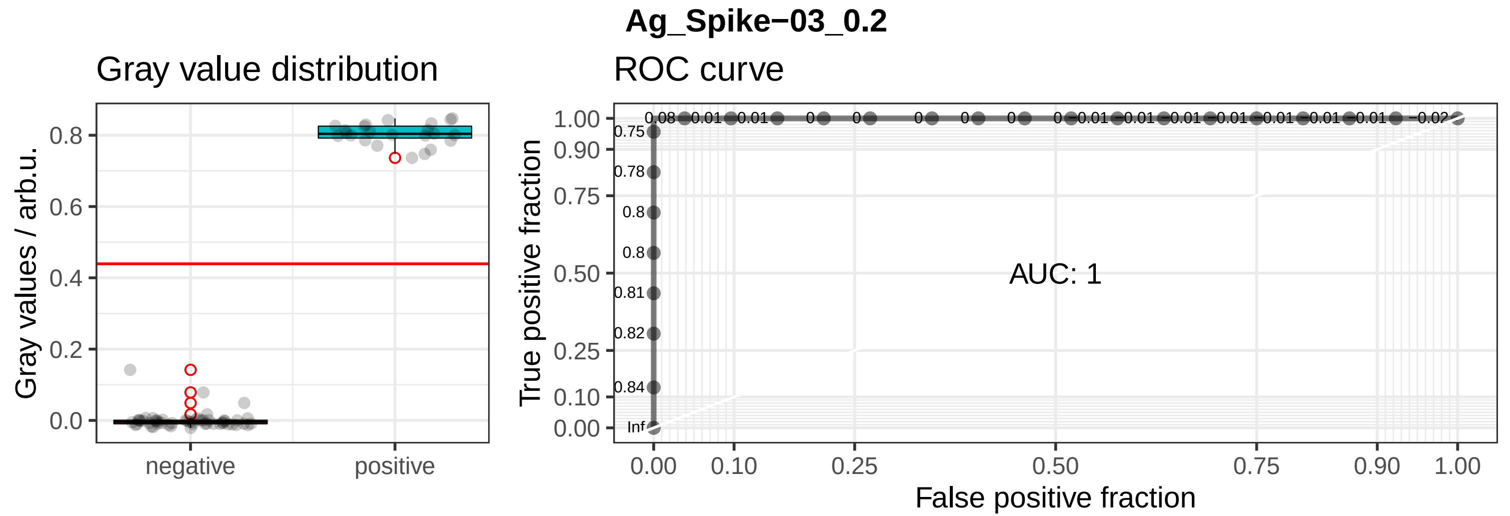

Ag_Spike−03_0.2
Gray value distribution
Gray values / arb.u.
0.8
0.6
0.4
0.2
0.0
negative
positive
ROC curve
True positive fraction
False positive fraction
1.00
0.90
0.75
0.50
0.25
0.10
0.00
0.00
0.10
0.25
0.50
0.75
0.90
1.00
AUC: 1
0.08
0.01
0.01
0
0
0
0
0
0
−0.01
−0.01
−0.01
−0.01
−0.01
−0.01
−0.01
−0.02
0.75
0.78
0.8
0.8
0.81
0.82
0.84
Inf

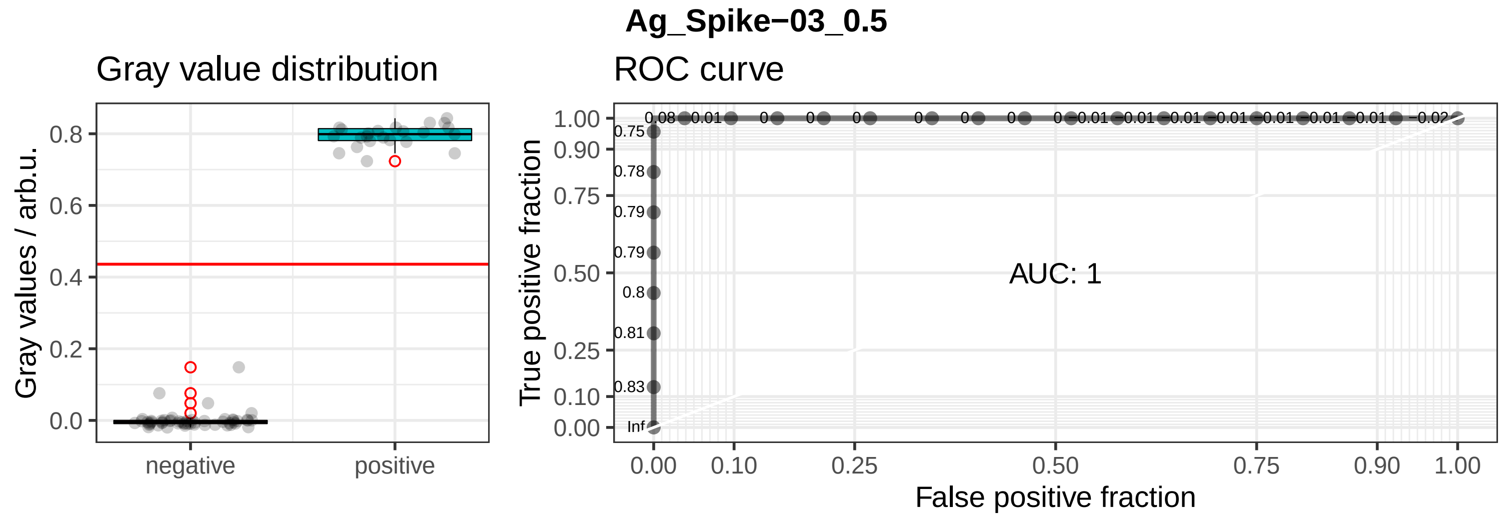
Ag_Spike−03_0.5
Gray value distribution
Gray values / arb.u.
0.0
0.2
0.4
0.6
0.8
negative
positive
ROC curve
True positive fraction
False positive fraction
0.00
0.10
0.25
0.50
0.75
0.90
1.00
AUC: 1
Inf
0.83
0.81
0.8
0.79
0.79
0.78
0.75
0.08
0.01
0
−0.01
−0.02

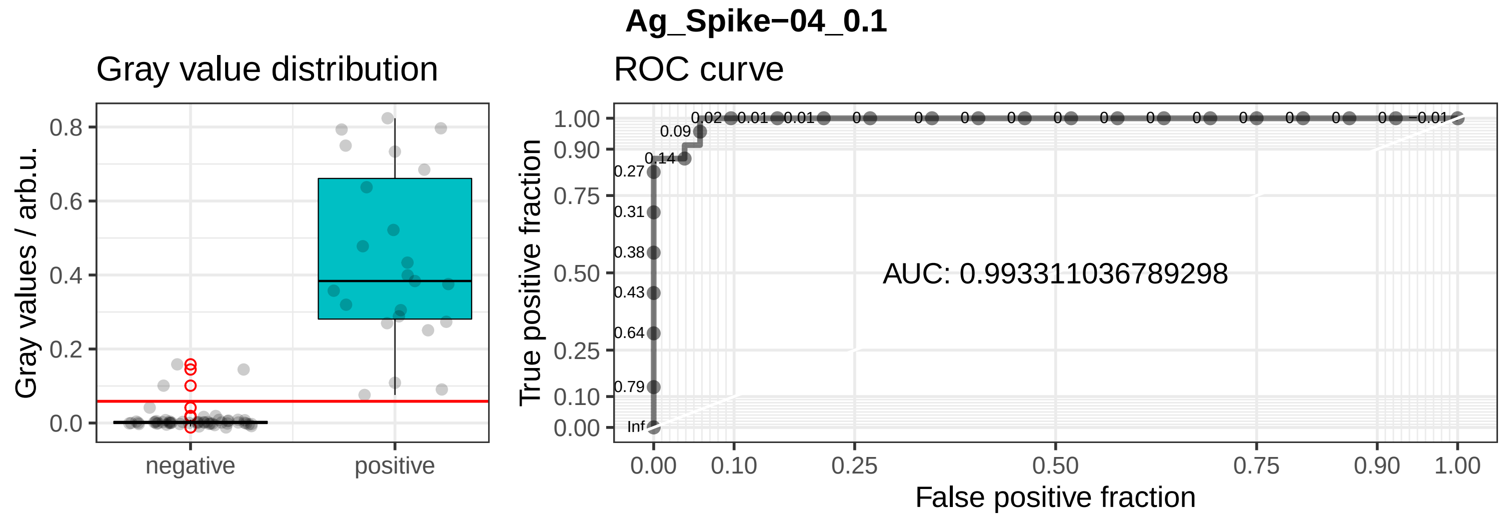

Ag_Spike−04_0.1
Gray value distribution
Gray values / arb.u.
0.8
0.6
0.4
0.2
0.0
negative
positive
ROC curve
True positive fraction
False positive fraction
1.00
0.90
0.75
0.50
0.25
0.10
0.00
0.00
0.10
0.25
0.50
0.75
0.90
1.00
AUC: 0.993311036789298
Inf
0.79
0.64
0.43
0.38
0.31
0.27
0.14
0.09
0.02
0.01
0.01
−0.01

Ag_Spike−04_0.2

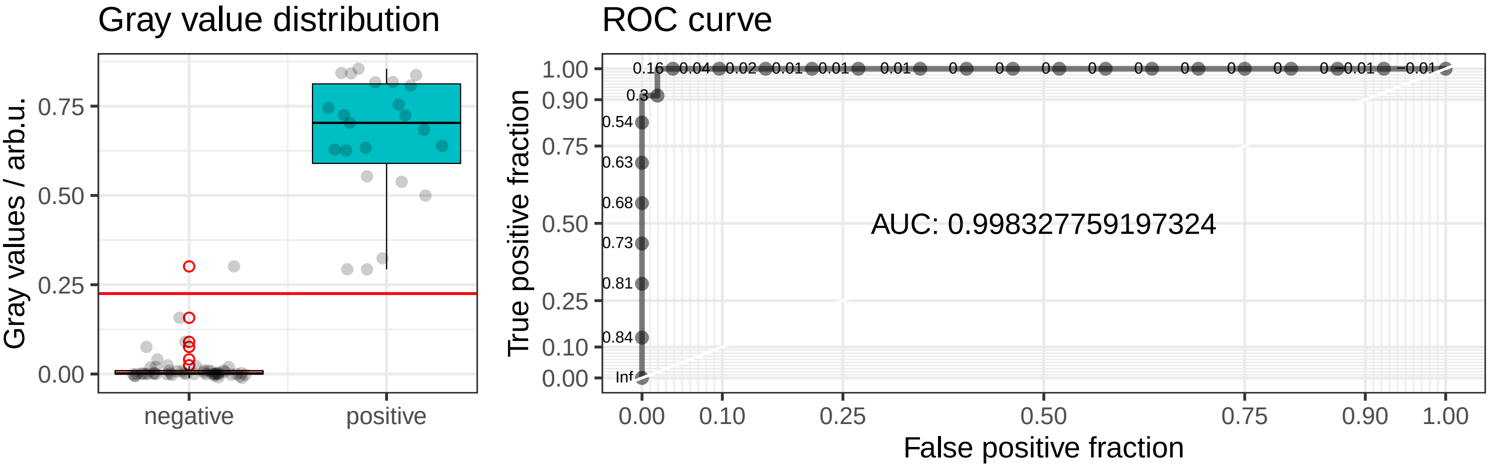

Ag_Spike−04_0.45

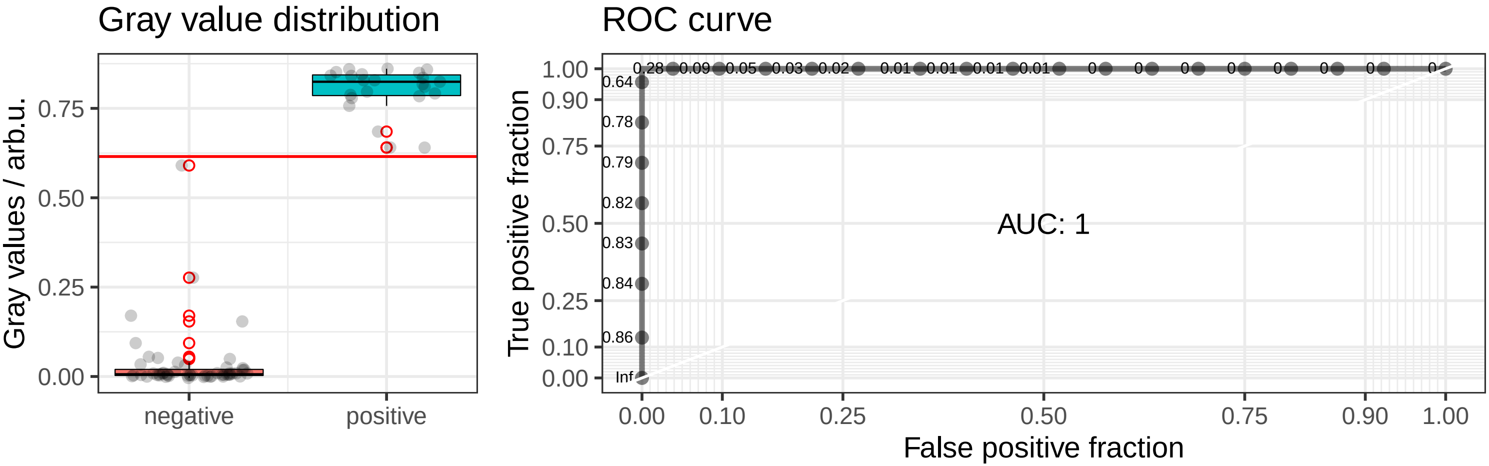

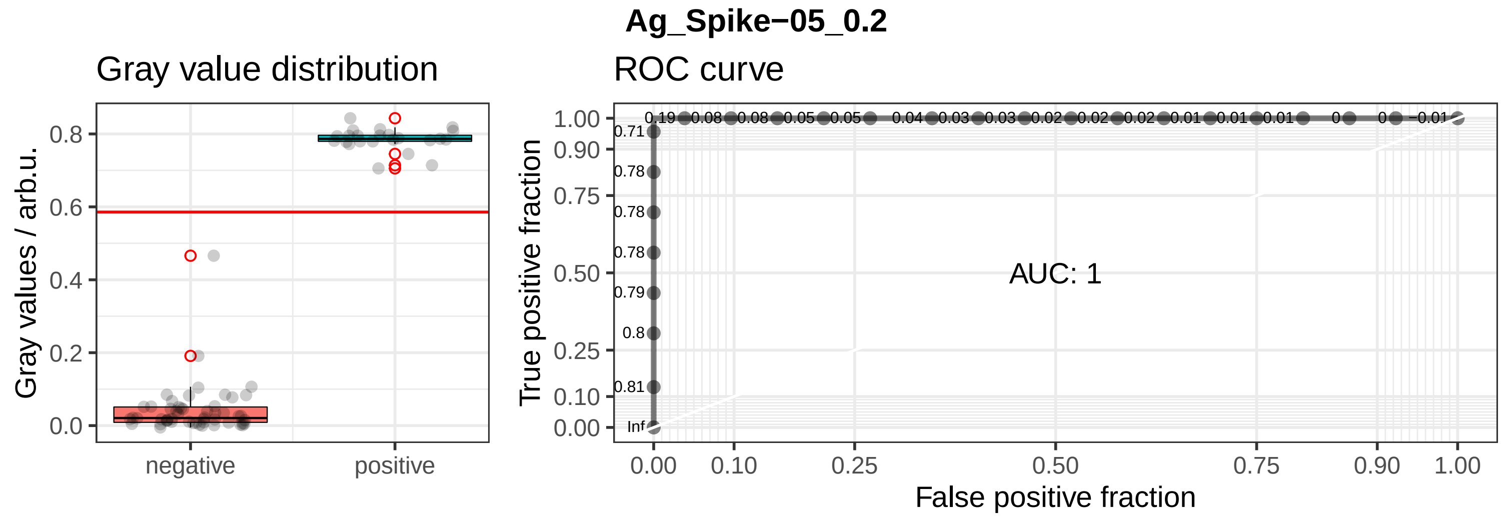
Ag_Spike−05_0.2
Gray value distribution
Gray values / arb.u.
negative
positive
ROC curve
True positive fraction
False positive fraction
AUC: 1
0.00
0.10
0.25
0.50
0.75
0.90
1.00
Inf
0.81
0.8
0.79
0.78
0.78
0.78
0.71
0.19
0.08
0.08
0.05
0.05
0.04
0.03
0.03
0.02
0.02
0.02
0.01
0.01
0.01
0
0
−0.01

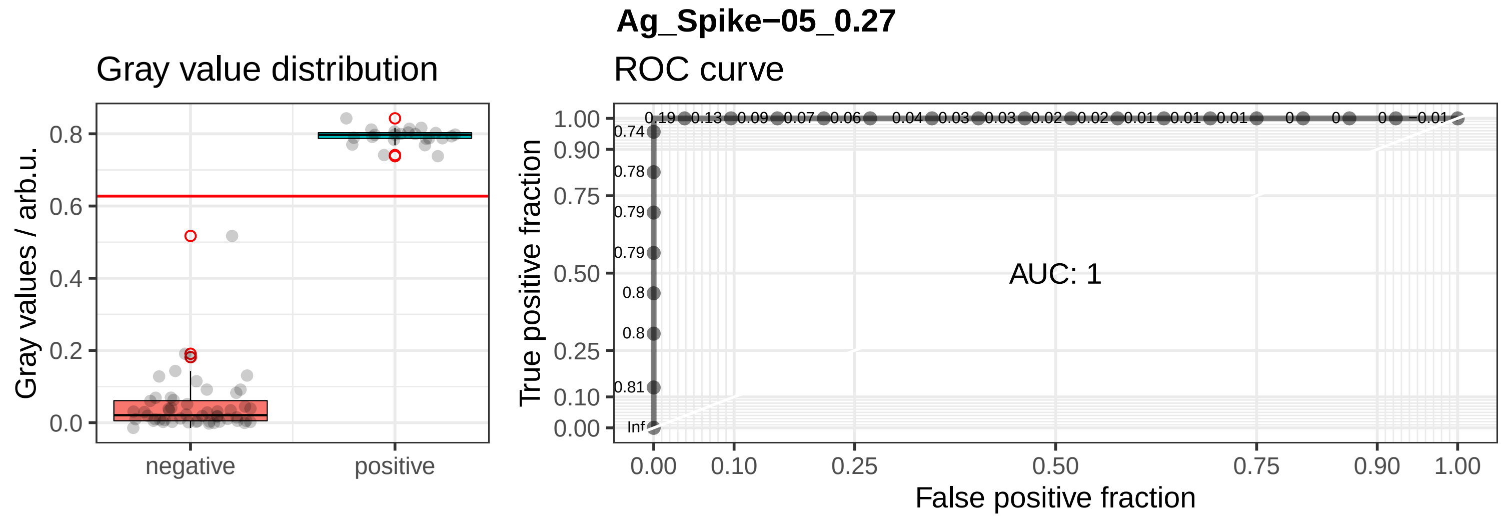
Ag_Spike−05_0.27
Gray value distribution
Gray values / arb.u.
0.0
0.2
0.4
0.6
0.8
negative
positive
ROC curve
True positive fraction
False positive fraction
0.00
0.10
0.25
0.50
0.75
0.90
1.00
AUC: 1
Inf
0.81
0.8
0.8
0.79
0.79
0.78
0.74
0.19
0.13
0.09
0.07
0.06
0.04
0.03
0.03
0.02
0.02
0.01
0.01
0.01
0
0
0
−0.01

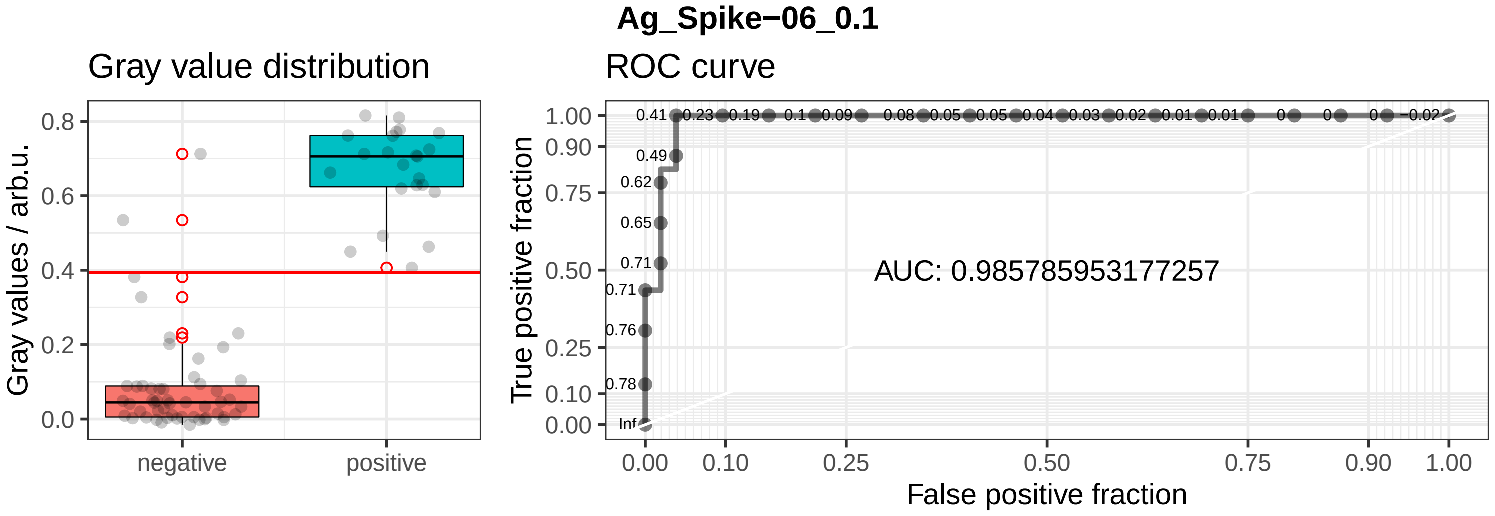
Ag_Spike−06_0.1
Gray value distribution
Gray values / arb.u.
0.0
0.2
0.4
0.6
0.8
negative
positive
ROC curve
True positive fraction
False positive fraction
0.00
0.10
0.25
0.50
0.75
0.90
1.00
AUC: 0.985785953177257
Inf
0.78
0.76
0.71
0.71
0.65
0.62
0.49
0.41
0.23
0.19
0.1
0.09
0.08
0.05
0.05
0.04
0.03
0.02
0.01
0.01
0
0
0
−0.02

Ag_Spike−06_0.2

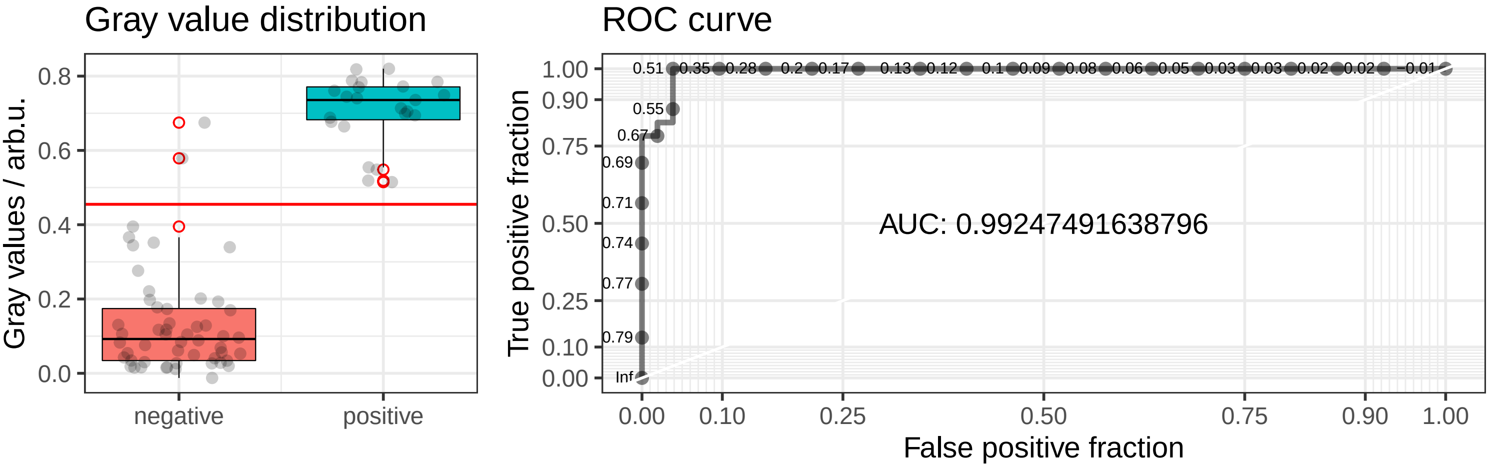

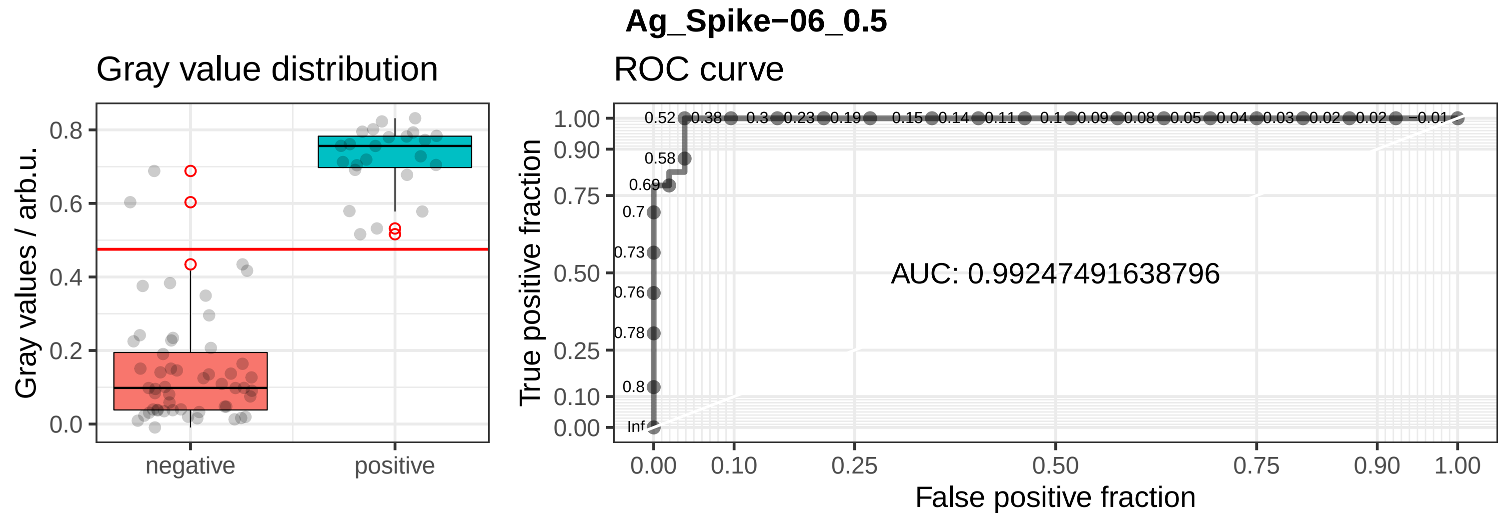
Ag_Spike−06_0.5
Gray value distribution
Gray values / arb.u.
0.0
0.2
0.4
0.6
0.8
negative
positive
ROC curve
True positive fraction
False positive fraction
0.00
0.10
0.25
0.50
0.75
0.90
1.00
AUC: 0.99247491638796
Inf
0.8
0.78
0.76
0.73
0.7
0.69
0.58
0.52
0.38
0.3
0.23
0.19
0.15
0.14
0.11
0.1
0.09
0.08
0.05
0.04
0.03
0.02
0.02
−0.01

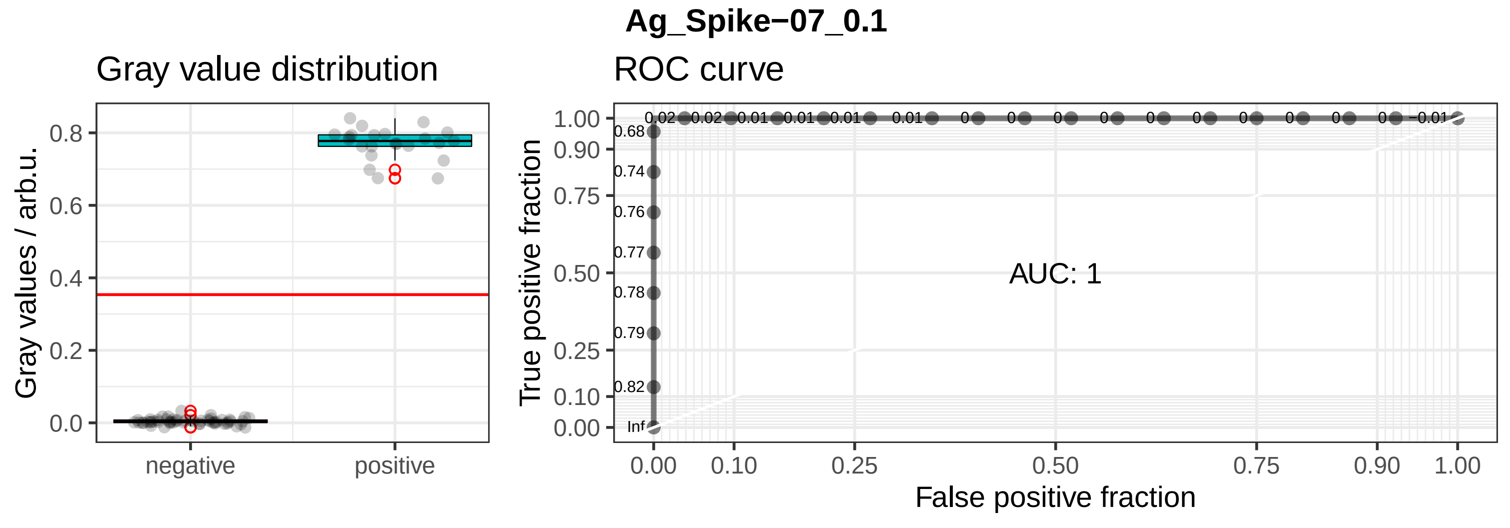

Ag_Spike−07_0.1
Gray value distribution
ROC curve
Gray values / arb.u.
0.8
0.6
0.4
0.2
0.0
negative
positive
True positive fraction
1.00
0.90
0.75
0.50
0.25
0.10
0.00
0.02
0.01
0
−0.01
0.68
0.74
0.76
0.77
0.78
0.79
0.82
Inf
AUC: 1
False positive fraction

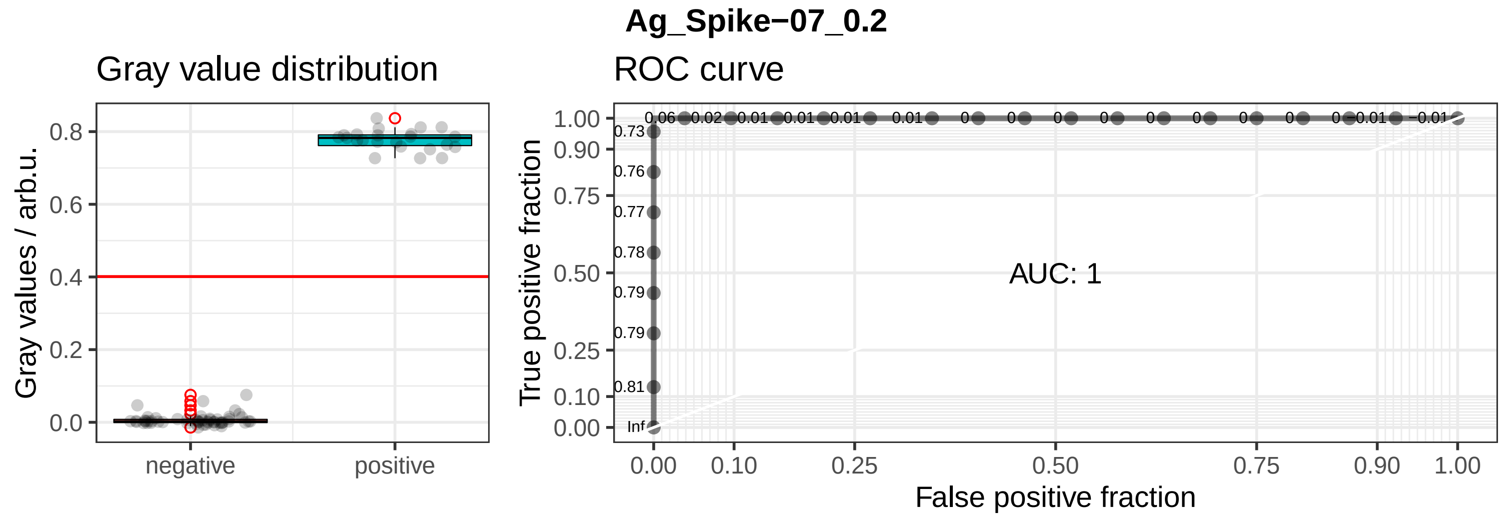
Ag_Spike−07_0.2
Gray value distribution
Gray values / arb.u.
0.0
0.2
0.4
0.6
0.8
negative
positive
ROC curve
True positive fraction
False positive fraction
0.00
0.10
0.25
0.50
0.75
0.90
1.00
AUC: 1
Inf
0.81
0.79
0.79
0.78
0.77
0.76
0.73
0.06
0.02
0.01
0.01
0.01
0.01
0
−0.01
−0.01

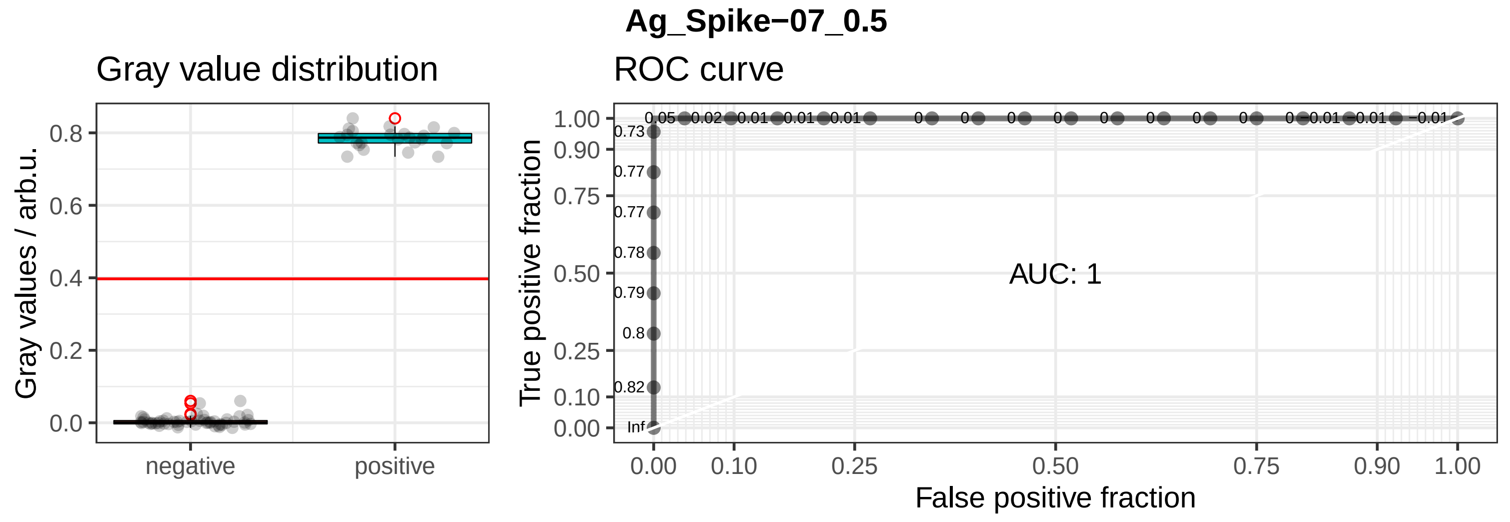

Ag_Spike−07_0.5
Gray value distribution
Gray values / arb.u.
0.0
0.2
0.4
0.6
0.8
negative
positive
ROC curve
True positive fraction
False positive fraction
0.00
0.10
0.25
0.50
0.75
0.90
1.00
AUC: 1
Inf
0.82
0.8
0.79
0.78
0.77
0.77
0.73
0.05
0.02
0.01
0.01
0.01
0
−0.01

Ag_Spike−08_0.1

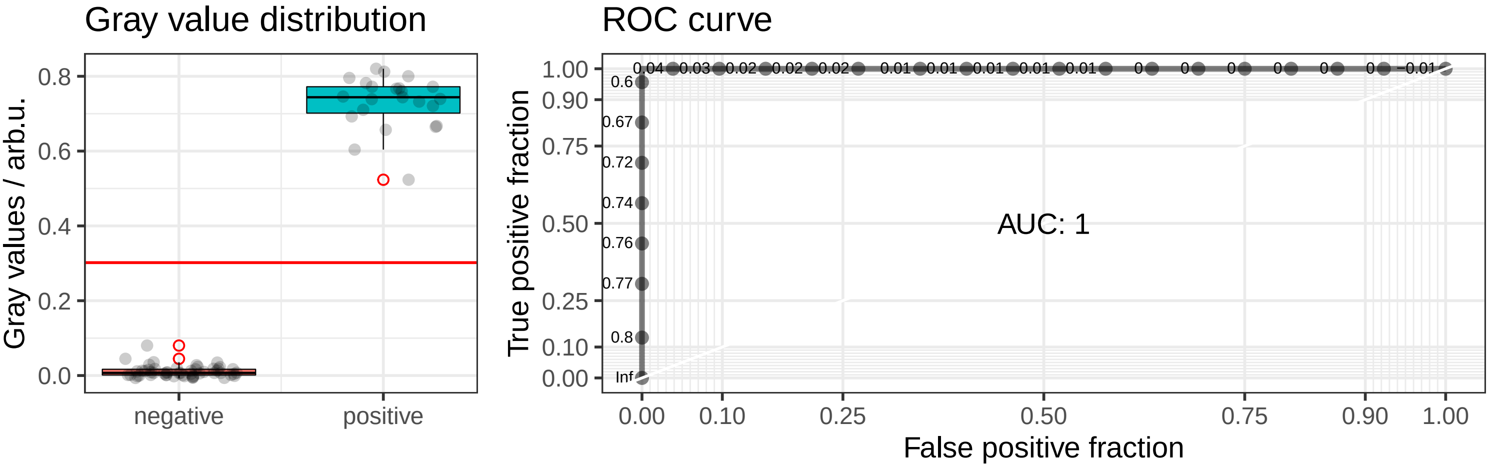

Ag_Spike−08_0.2

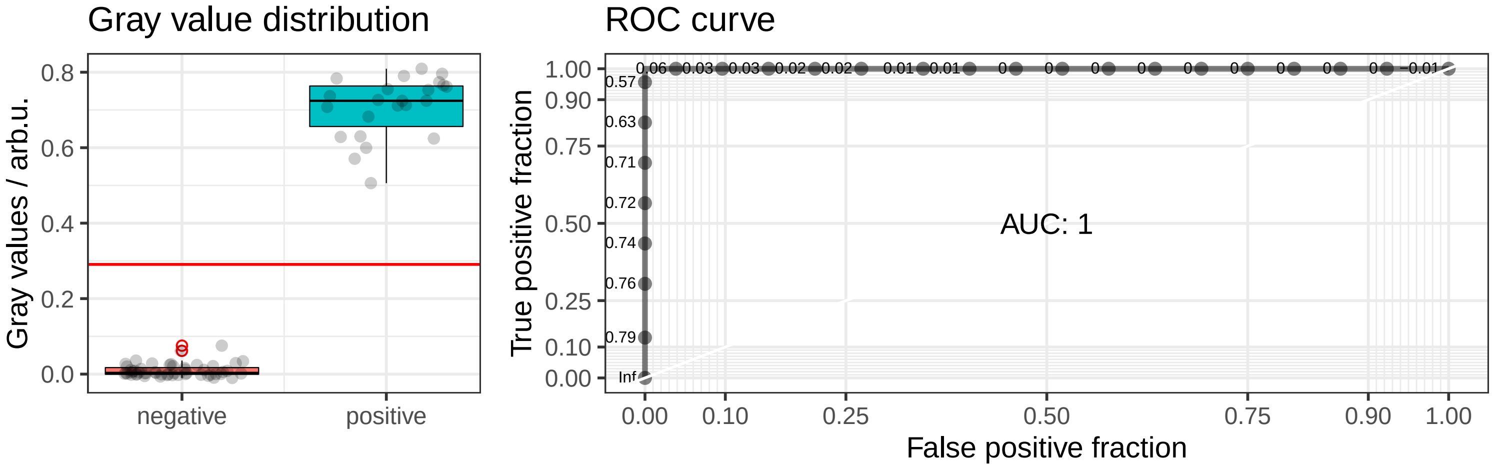

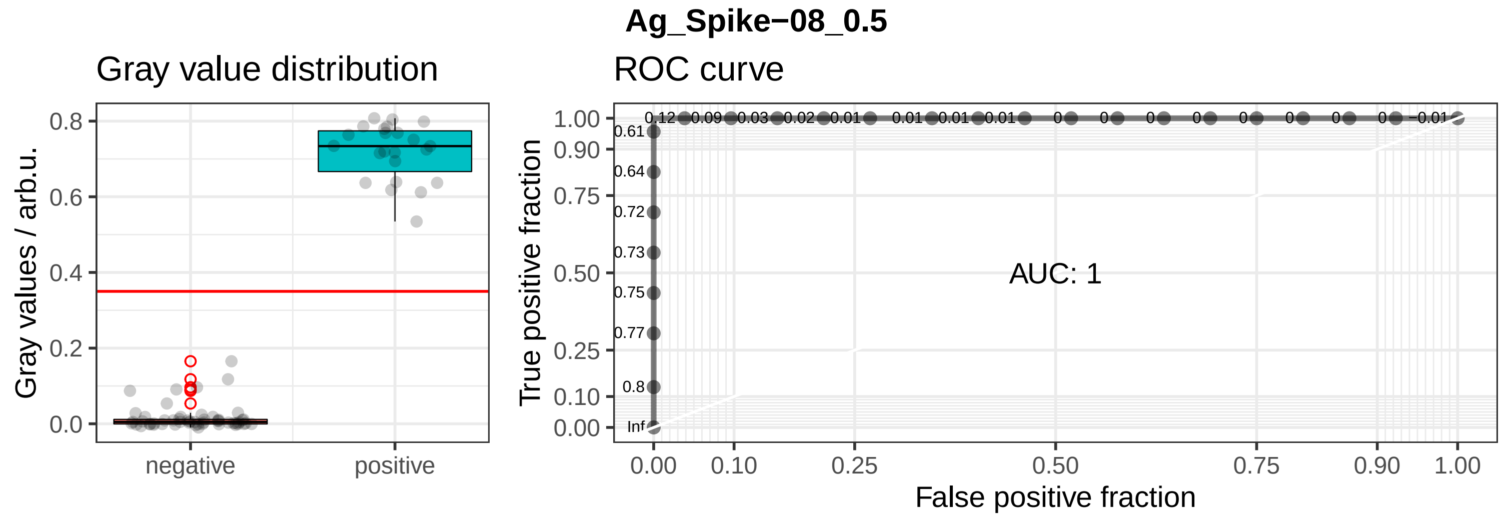
Ag_Spike−08_0.5
Gray value distribution
Gray values / arb.u.
0.8
0.6
0.4
0.2
0.0
negative
positive
ROC curve
True positive fraction
False positive fraction
AUC: 1
0.00
0.10
0.25
0.50
0.75
0.90
1.00
0.12
0.09
0.03
0.02
0.01
0
−0.01
0.61
0.64
0.72
0.73
0.75
0.77
0.8
Inf

Ag_Spike−09_0.1

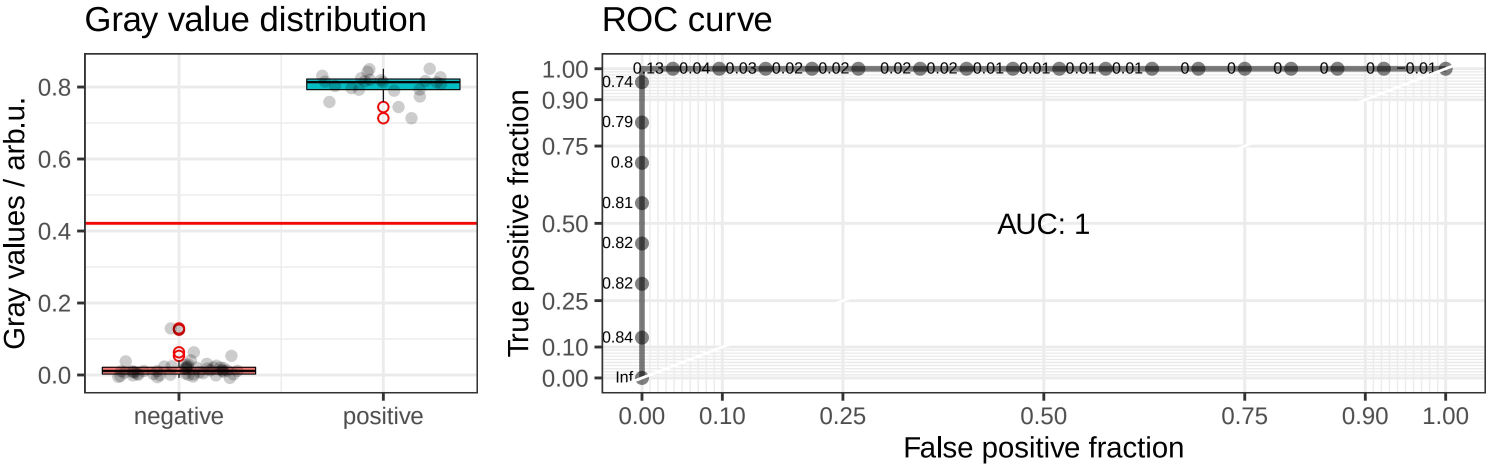

Ag_Spike−09_0.2

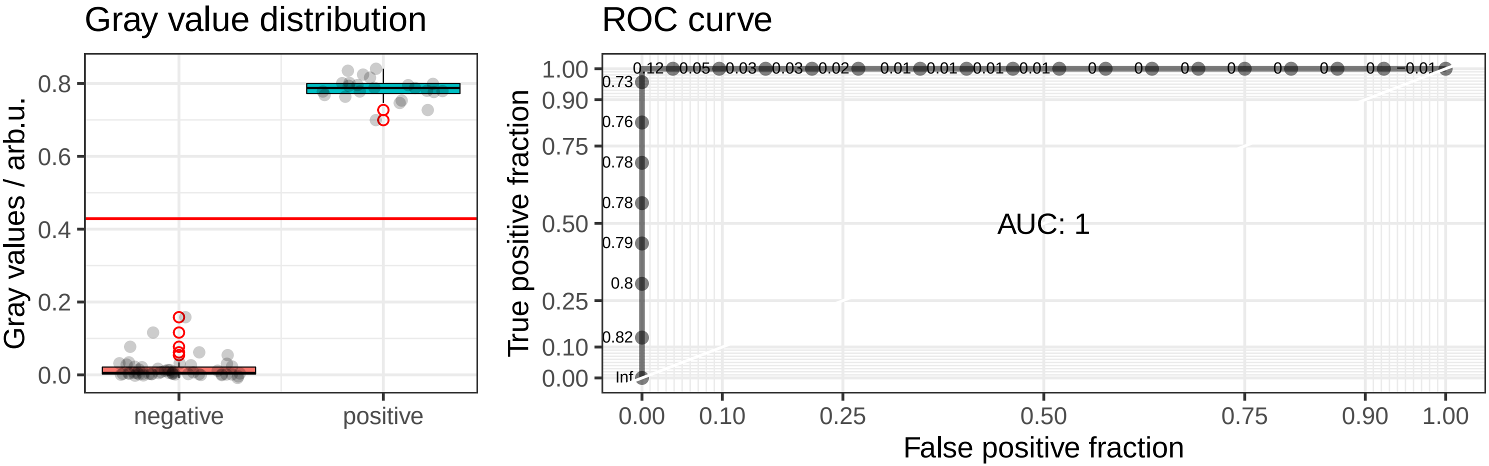

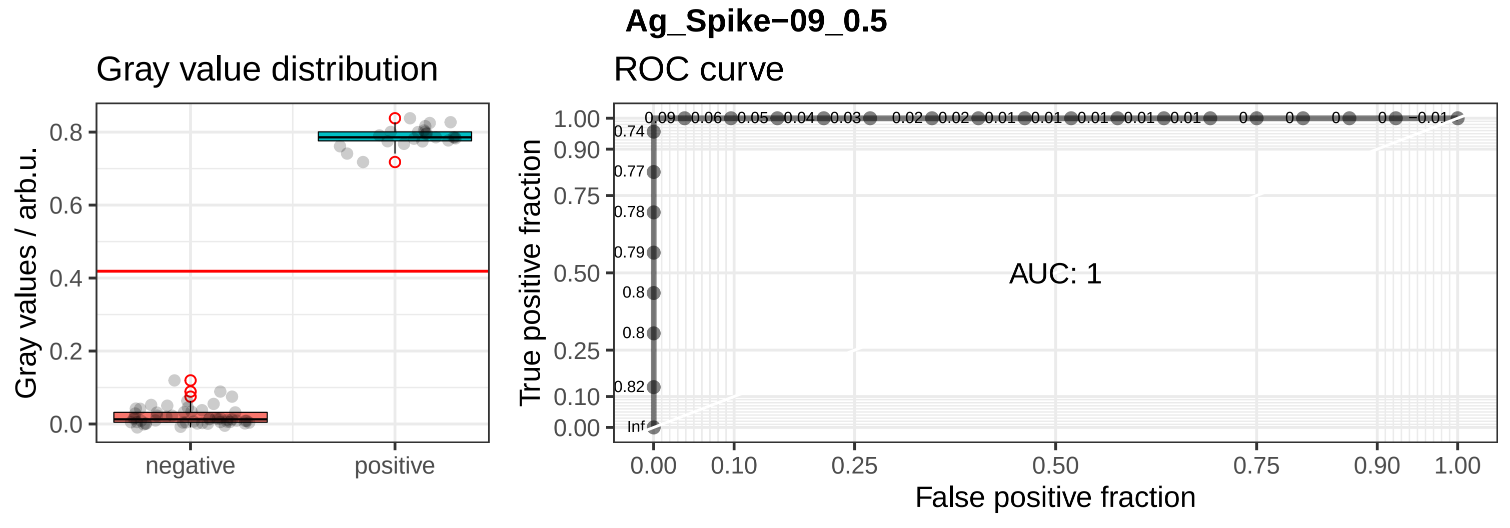

Ag_Spike−09_0.5
Gray value distribution
Gray values / arb.u.
0.8
0.6
0.4
0.2
0.0
negative
positive
ROC curve
True positive fraction
False positive fraction
1.00
0.90
0.75
0.50
0.25
0.10
0.00
0.00
0.10
0.25
0.50
0.75
0.90
1.00
AUC: 1
Inf
0.82
0.8
0.8
0.79
0.78
0.77
0.74
0.09
0.06
0.05
0.04
0.03
0.02
0.02
0.01
0.01
0.01
0.01
0.01
0
0
0
0
−0.01

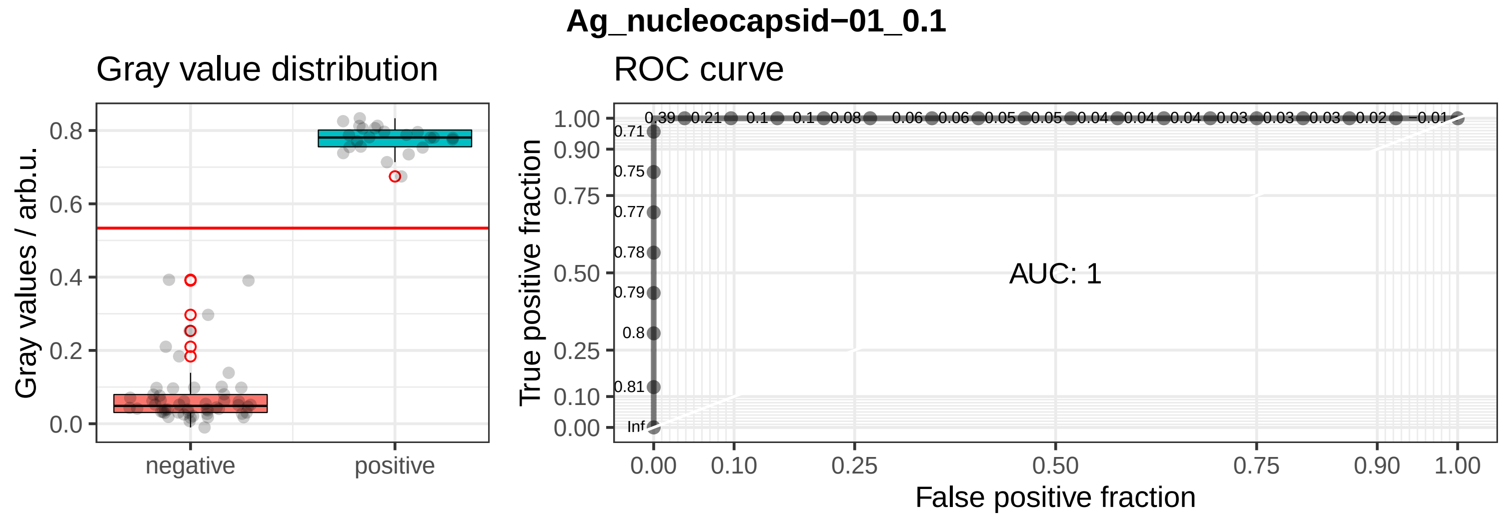
Ag_nucleocapsid−01_0.1
Gray value distribution
Gray values / arb.u.
0.8
0.6
0.4
0.2
0.0
negative
positive
ROC curve
True positive fraction
1.00
0.90
0.75
0.50
0.25
0.10
0.00
0.39 0.21 0.1 0.1 0.08 0.06 0.06 0.05 0.05 0.04 0.04 0.04 0.03 0.03 0.03 0.02 −0.01
0.71
0.75
0.77
0.78
0.79
0.8
0.81
Inf
AUC: 1
0.00 0.10 0.25 0.50 0.75 0.90 1.00
False positive fraction

Ag_nucleocapsid−01_0.2

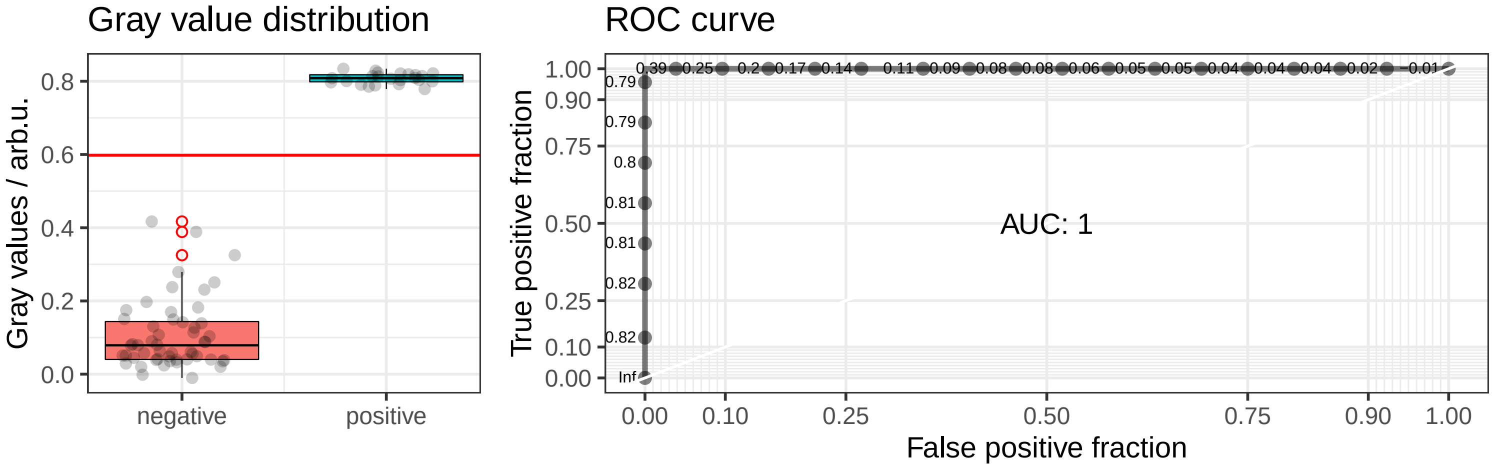

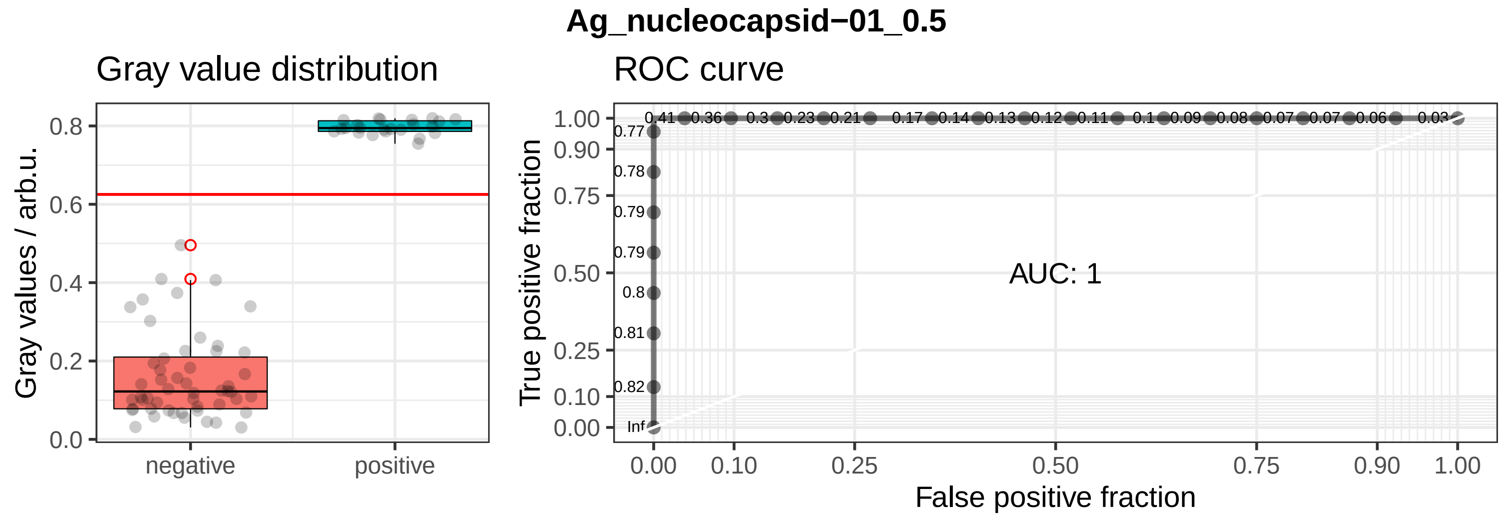

Ag_nucleocapsid−01_0.5
Gray value distribution
Gray values / arb.u.
0.0
0.2
0.4
0.6
0.8
negative
positive
ROC curve
True positive fraction
False positive fraction
AUC: 1
0.00
0.10
0.25
0.50
0.75
0.90
1.00
Inf
0.82
0.81
0.8
0.79
0.79
0.78
0.77
0.41
0.36
0.3
0.23
0.21
0.17
0.14
0.13
0.12
0.11
0.1
0.09
0.08
0.07
0.07
0.06
0.03

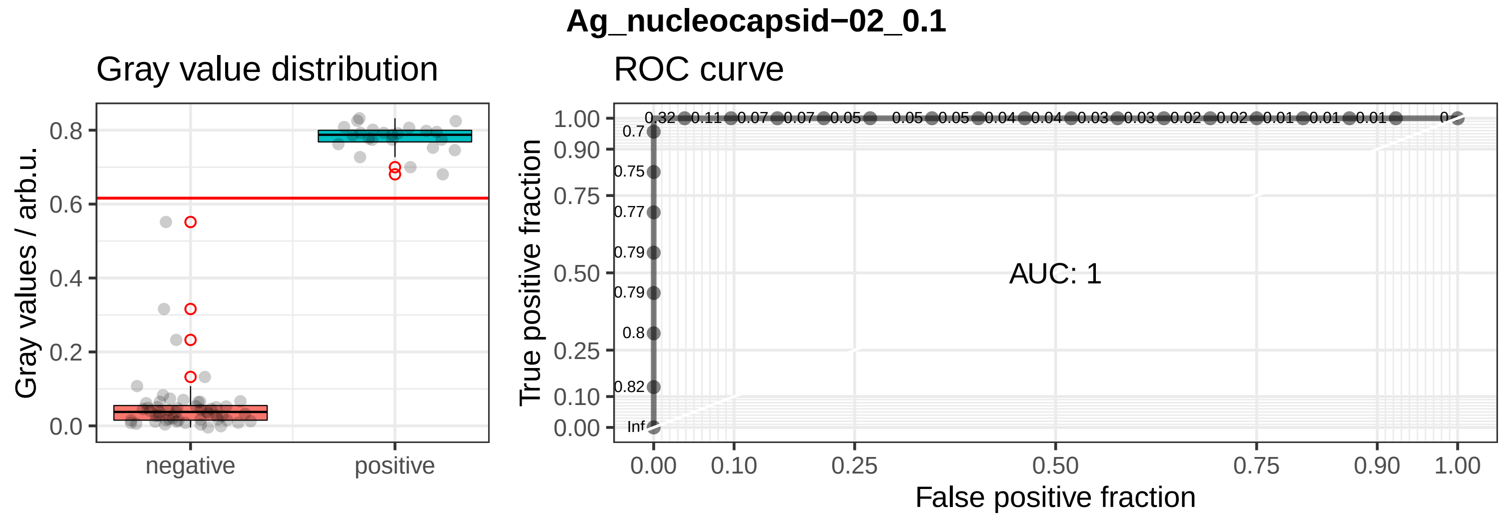
Ag_nucleocapsid−02_0.1
Gray value distribution
Gray values / arb.u.
0.0
0.2
0.4
0.6
0.8
negative
positive
ROC curve
True positive fraction
False positive fraction
0.00
0.10
0.25
0.50
0.75
0.90
1.00
AUC: 1
0.32
0.11
0.07
0.07
0.05
0.05
0.05
0.04
0.04
0.03
0.03
0.02
0.02
0.01
0.01
0.01
0
0.7
0.75
0.77
0.79
0.79
0.8
0.82
Inf

Ag_nucleocapsid−02_0.2

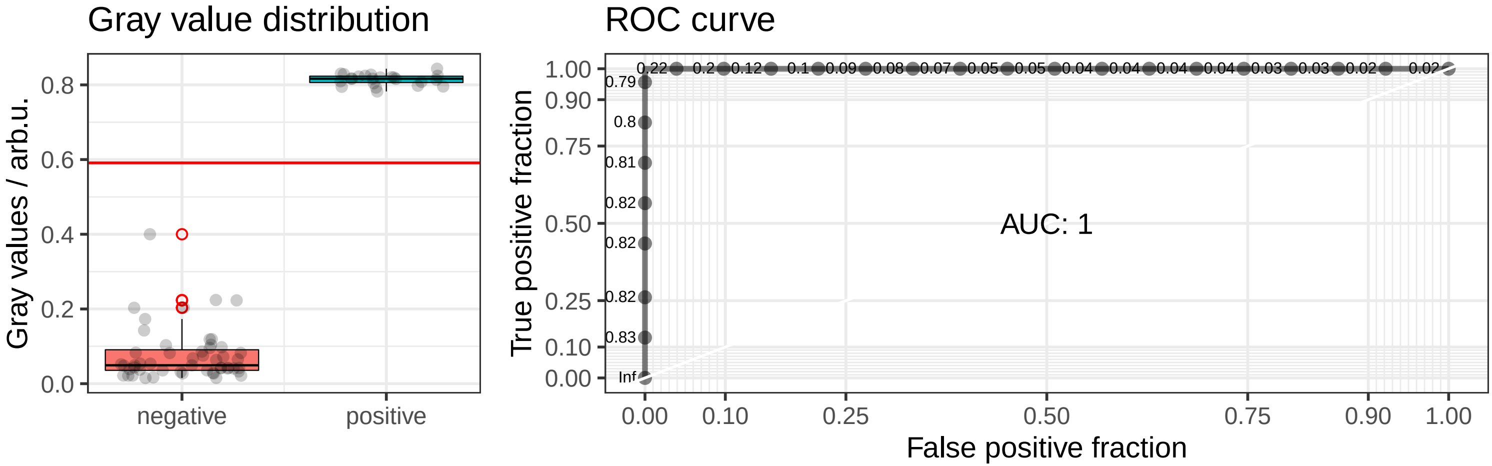

Ag_nucleocapsid−02_0.5

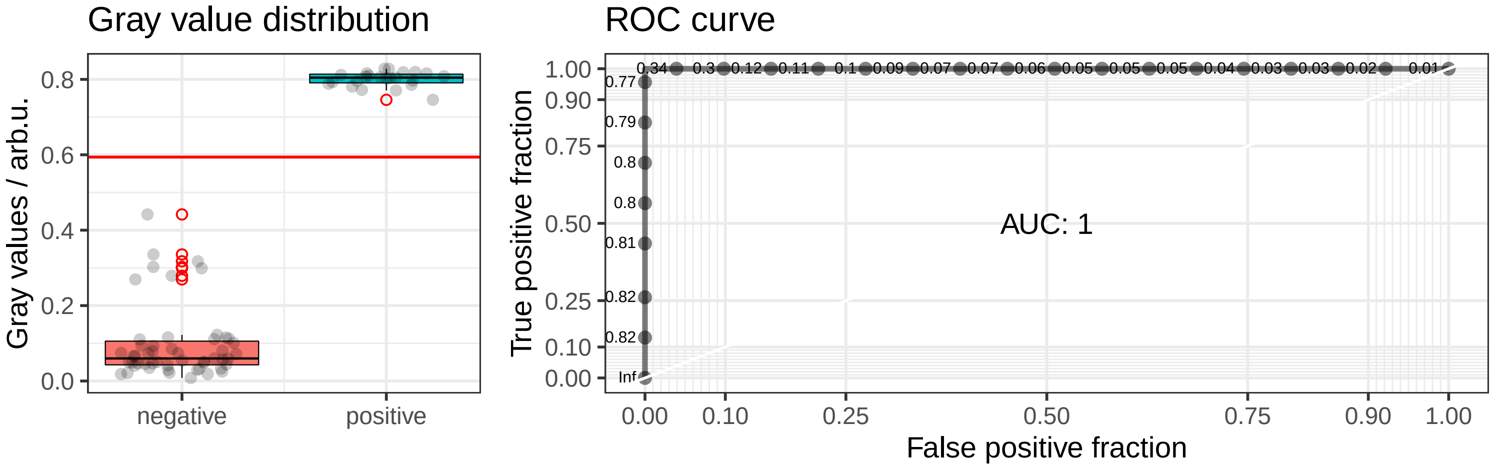

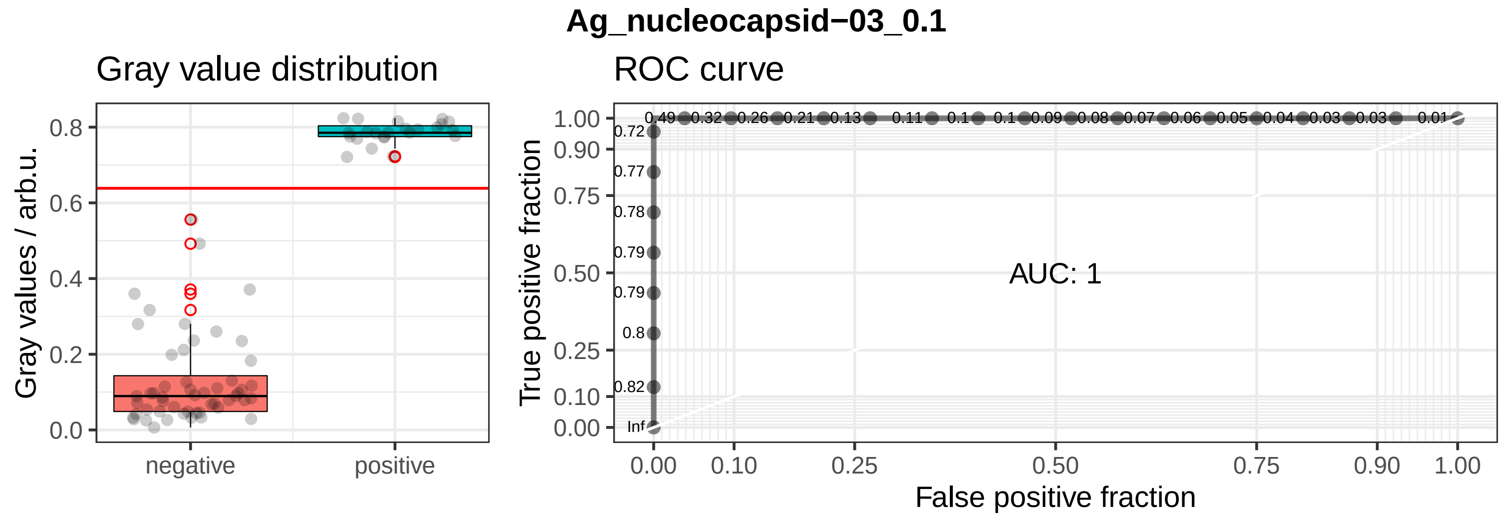

Ag_nucleocapsid−03_0.1
Gray value distribution
Gray values / arb.u.
0.0
0.2
0.4
0.6
0.8
negative
positive
ROC curve
True positive fraction
False positive fraction
0.00
0.10
0.25
0.50
0.75
0.90
1.00
AUC: 1
Inf
0.82
0.8
0.79
0.79
0.78
0.77
0.72
0.49
0.32
0.26
0.21
0.13
0.11
0.1
0.1
0.09
0.08
0.07
0.06
0.05
0.04
0.03
0.03
0.01

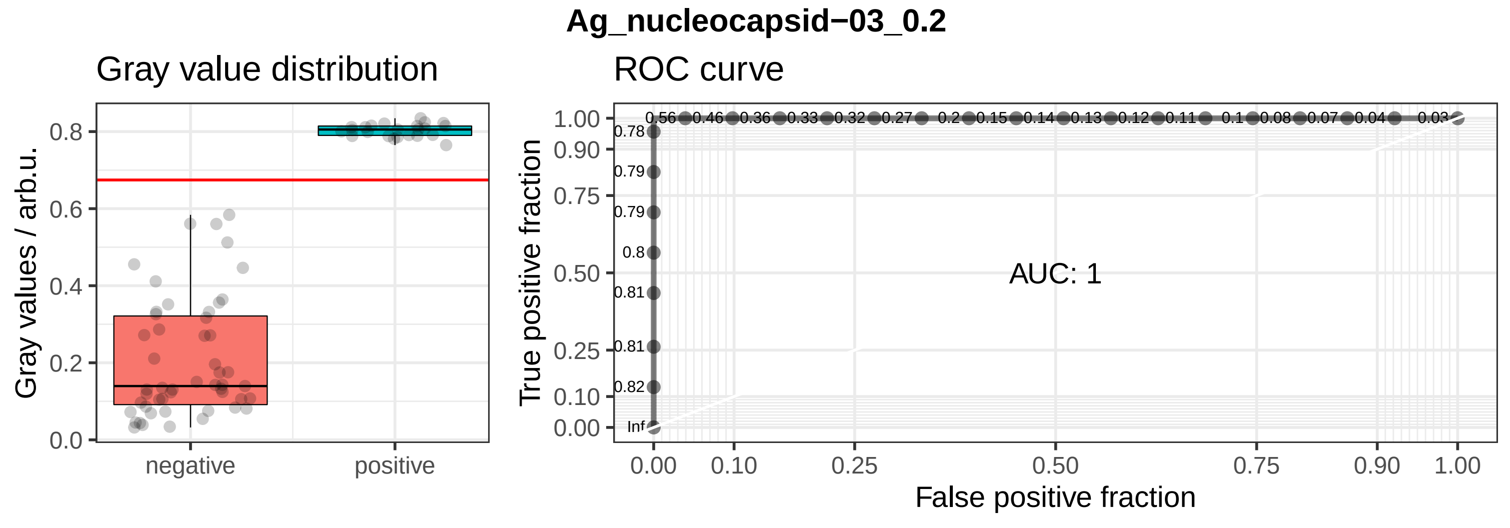
Ag_nucleocapsid−03_0.2
Gray value distribution
ROC curve
Gray values / arb.u.
negative
positive
True positive fraction
False positive fraction
AUC: 1

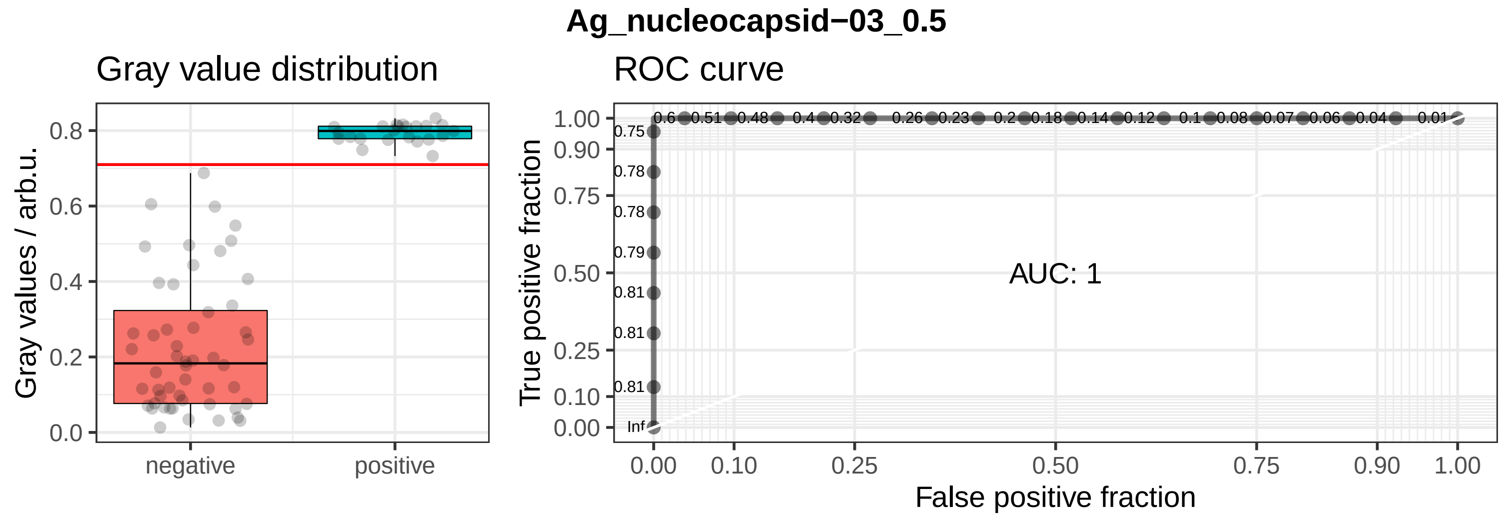

Ag_nucleocapsid−03_0.5
Gray value distribution
Gray values / arb.u.
0.0
0.2
0.4
0.6
0.8
negative
positive
ROC curve
True positive fraction
False positive fraction
0.00
0.10
0.25
0.50
0.75
0.90
1.00
AUC: 1
Inf
0.81
0.81
0.81
0.79
0.78
0.78
0.75
0.6
0.51
0.48
0.4
0.32
0.26
0.23
0.2
0.18
0.14
0.12
0.1
0.08
0.07
0.06
0.04
0.01

Ag_nucleocapsid−04_0.1

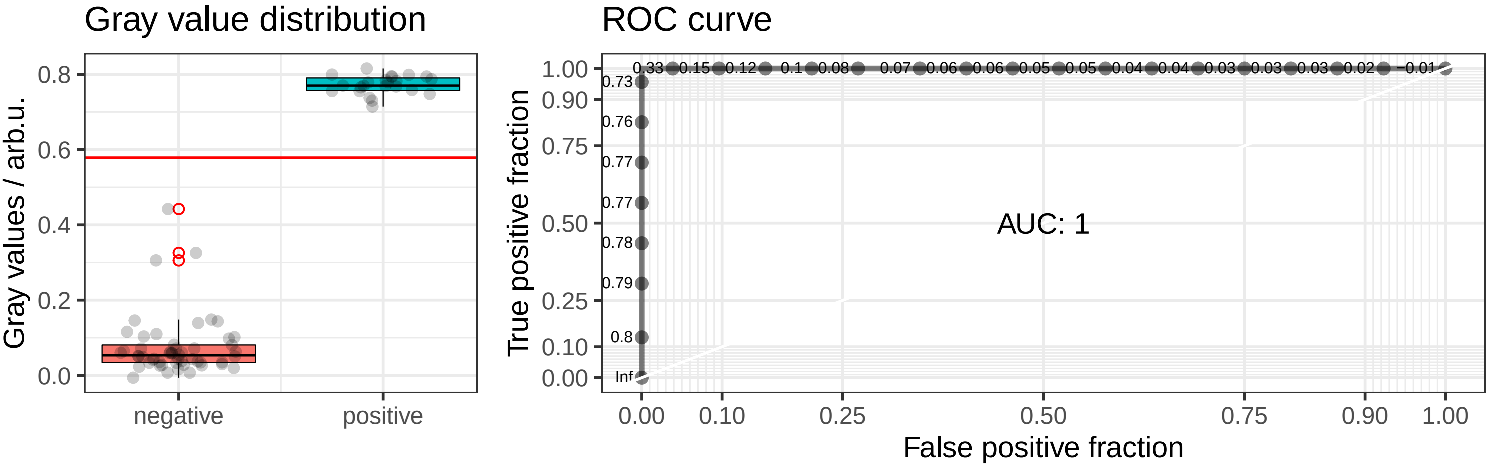

Ag_nucleocapsid−04_0.2

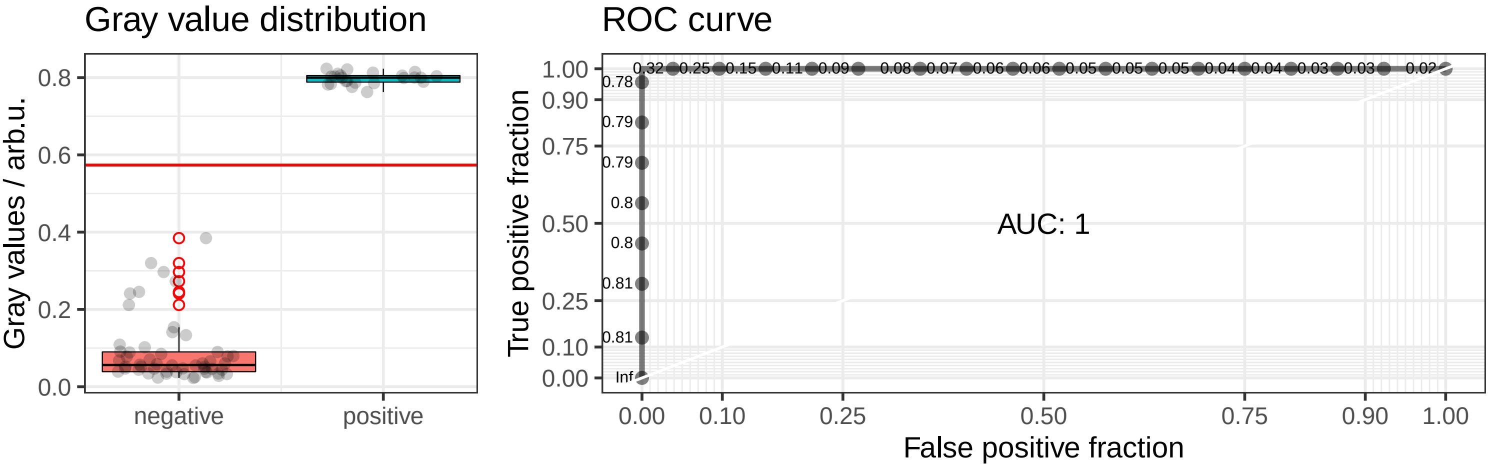

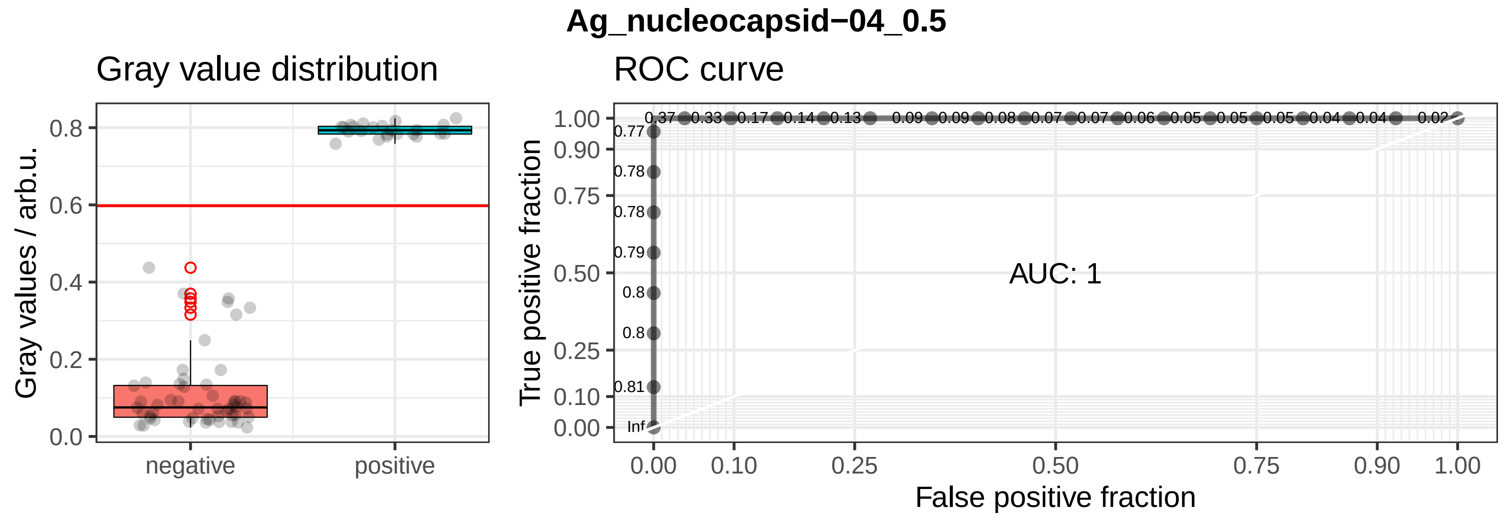
Ag_nucleocapsid−04_0.5
Gray value distribution
Gray values / arb.u.
0.0
0.2
0.4
0.6
0.8
negative
positive
ROC curve
True positive fraction
False positive fraction
0.00
0.10
0.25
0.50
0.75
0.90
1.00
AUC: 1
Inf
0.81
0.8
0.8
0.79
0.78
0.78
0.77
0.37
0.33
0.17
0.14
0.13
0.09
0.09
0.08
0.07
0.07
0.06
0.05
0.05
0.05
0.04
0.04
0.02

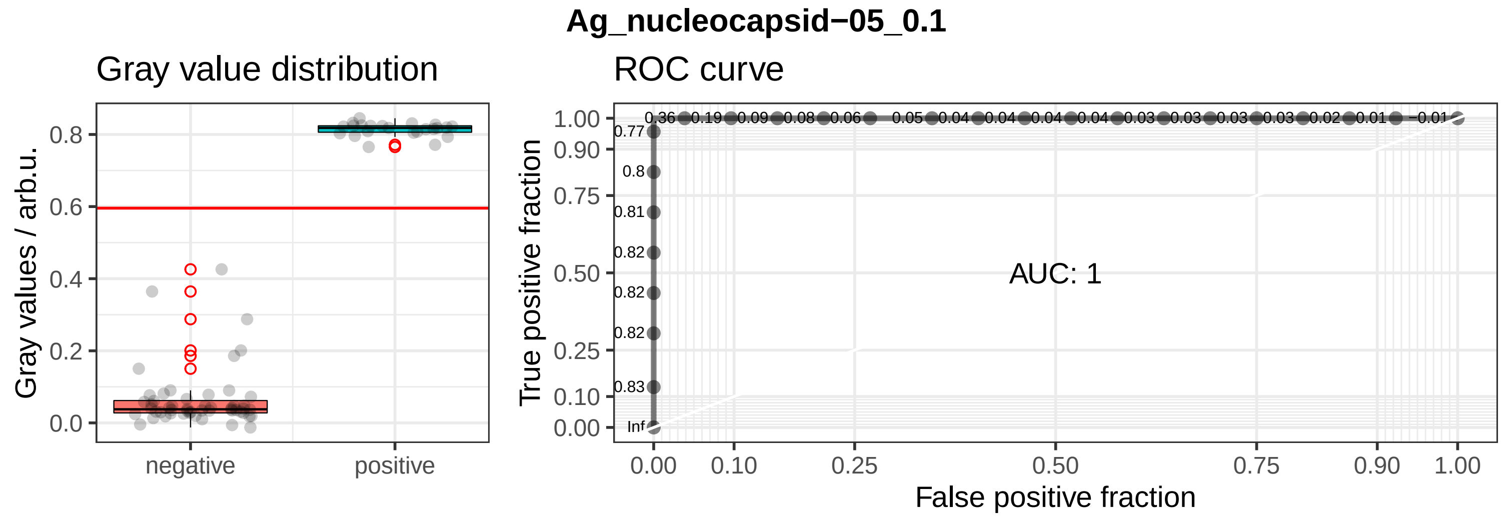
Ag_nucleocapsid−05_0.1
Gray value distribution
Gray values / arb.u.
0.8
0.6
0.4
0.2
0.0
negative
positive
ROC curve
True positive fraction
1.00
0.90
0.75
0.50
0.25
0.10
0.00
0.36
0.19
0.09
0.08
0.06
0.05
0.04
0.04
0.04
0.04
0.03
0.03
0.03
0.03
0.02
0.01
−0.01
0.77
0.8
0.81
0.82
0.82
0.82
0.83
Inf
AUC: 1
0.00
0.10
0.25
0.50
0.75
0.90
1.00
False positive fraction

Ag_nucleocapsid−05_0.2

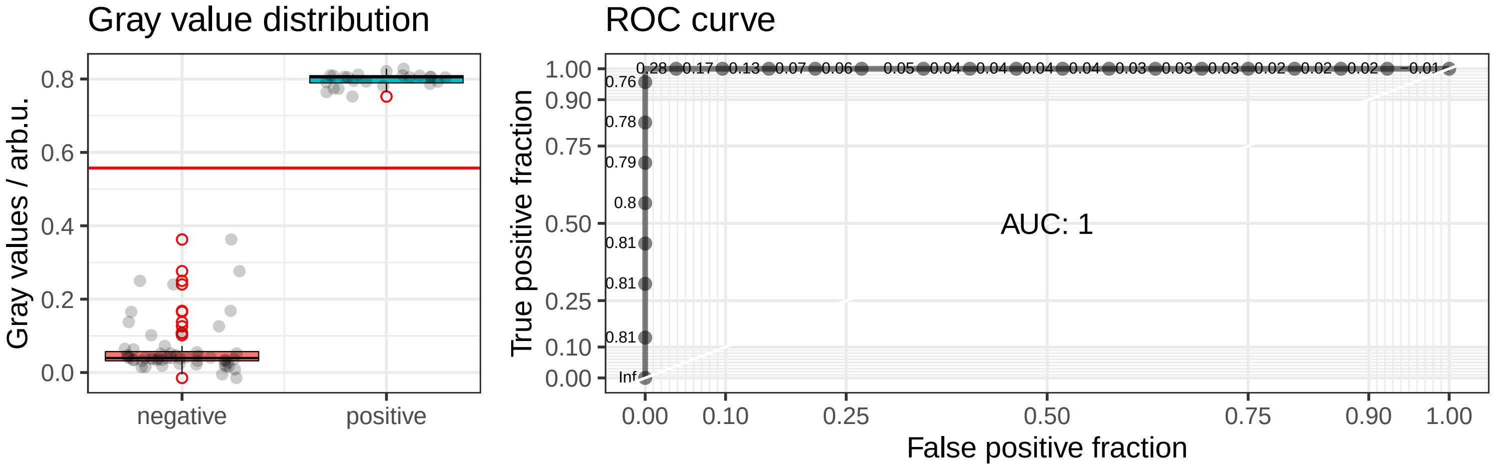

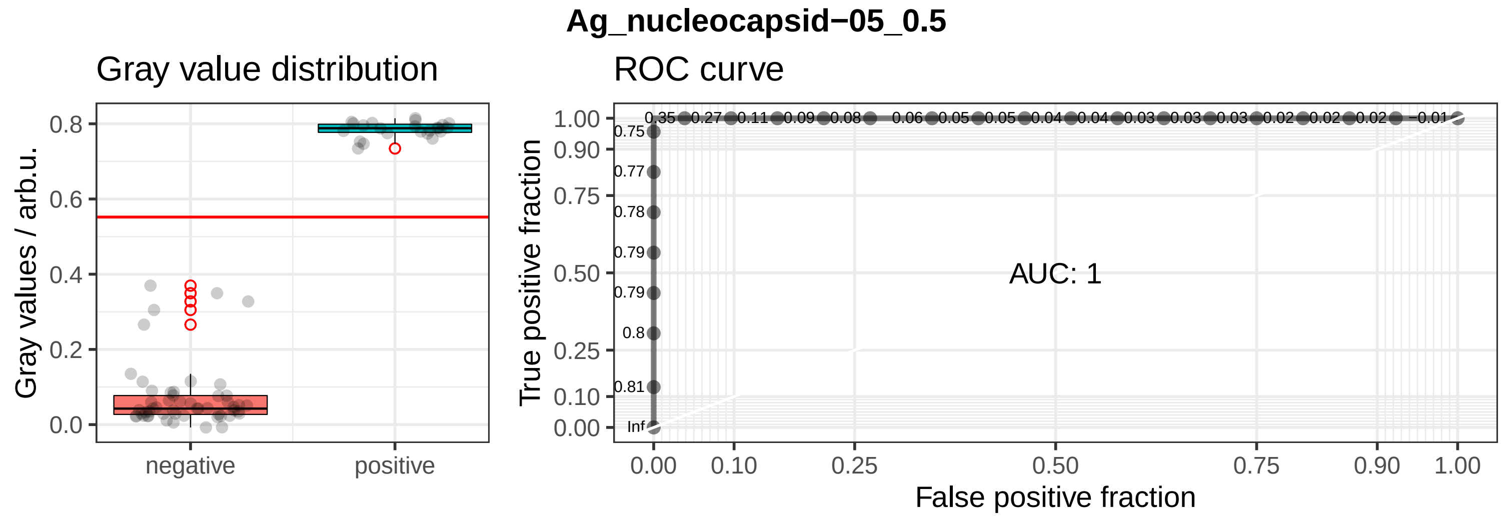
Ag_nucleocapsid−05_0.5
Gray value distribution
Gray values / arb.u.
0.8
0.6
0.4
0.2
0.0
negative
positive
ROC curve
True positive fraction
1.00
0.90
0.75
0.50
0.25
0.10
0.00
0.35 0.27 0.11 0.09 0.08 0.06 0.05 0.05 0.04 0.04 0.03 0.03 0.03 0.02 0.02 0.02 −0.01
0.75
0.77
0.78
0.79
0.79
0.8
0.81
Inf
AUC: 1
0.00 0.10 0.25 0.50 0.75 0.90 1.00
False positive fraction

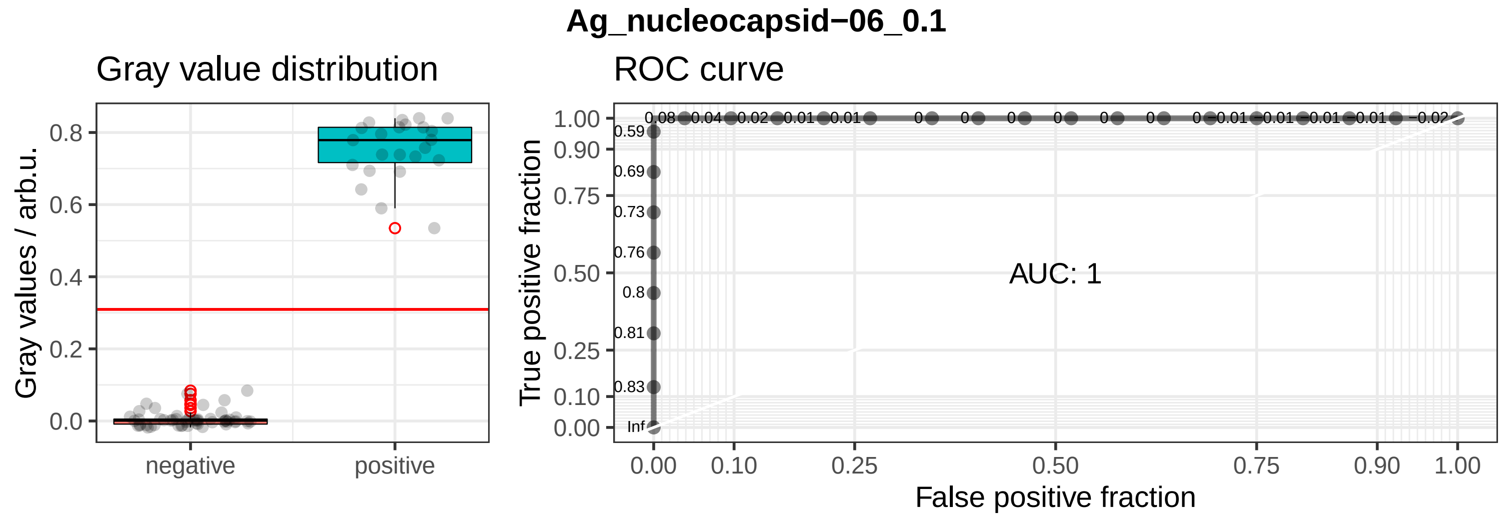
Ag_nucleocapsid−06_0.1
Gray value distribution
Gray values / arb.u.
0.8
0.6
0.4
0.2
0.0
negative
positive
ROC curve
True positive fraction
False positive fraction
1.00
0.90
0.75
0.50
0.25
0.10
0.00
0.00
0.10
0.25
0.50
0.75
0.90
1.00
AUC: 1
0.08
0.04
0.02
0.01
0.01
0
0
0
0
0
0
0
−0.01
−0.01
−0.01
−0.01
−0.02
0.59
0.69
0.73
0.76
0.8
0.81
0.83
Inf

Ag_nucleocapsid−06_0.2

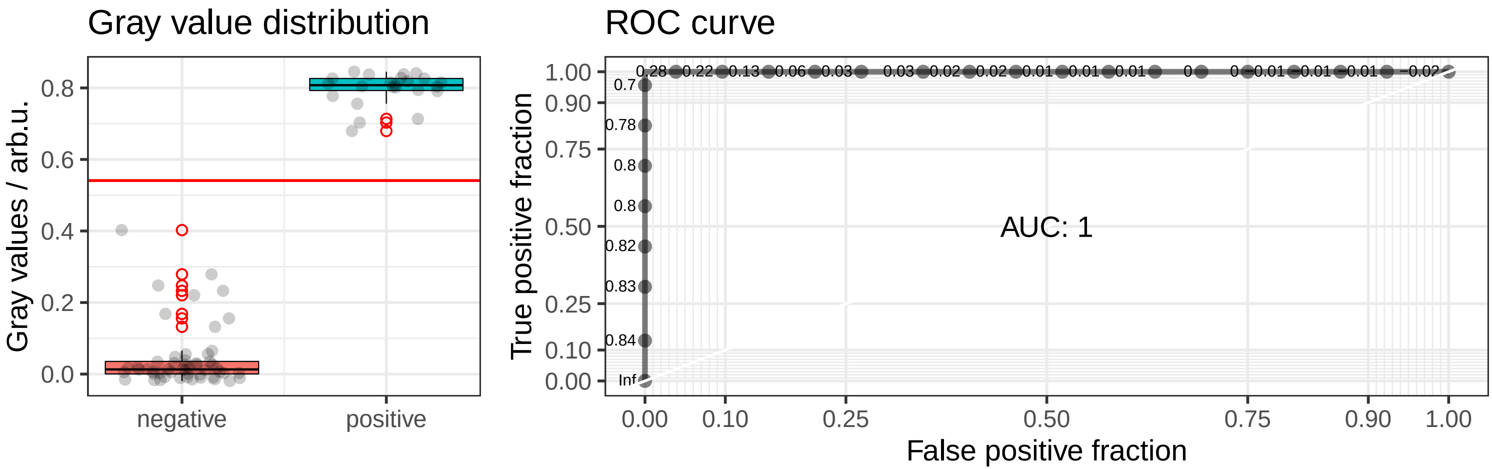

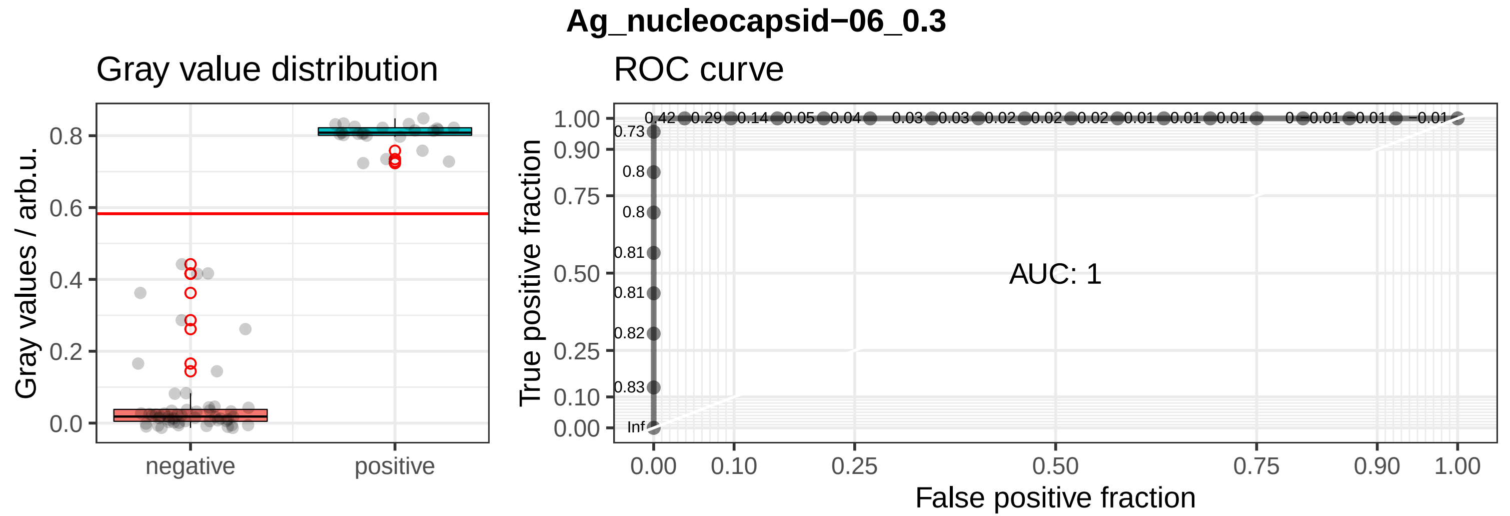
Ag_nucleocapsid−06_0.3
Gray value distribution
Gray values / arb.u.
0.0
0.2
0.4
0.6
0.8
negative
positive
ROC curve
True positive fraction
False positive fraction
0.00
0.10
0.25
0.50
0.75
0.90
1.00
AUC: 1
Inf
0.83
0.82
0.81
0.81
0.8
0.8
0.73
0.42
0.29
0.14
0.05
0.04
0.03
0.03
0.02
0.02
0.02
0.01
0.01
0.01
0
−0.01
−0.01
−0.01

Ag_nucleocapsid−07_0.1

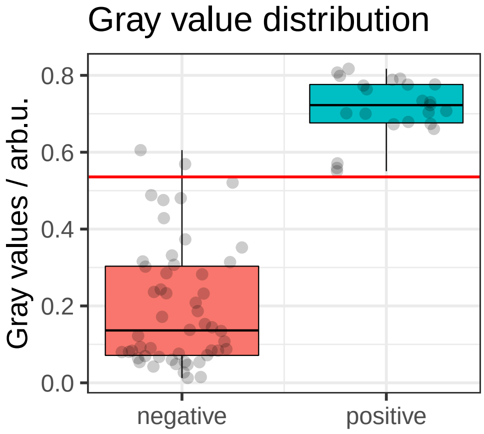

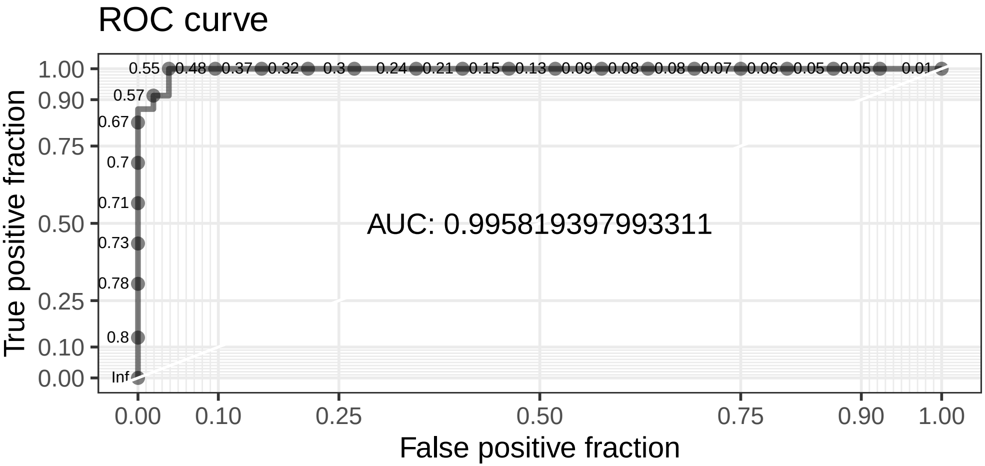

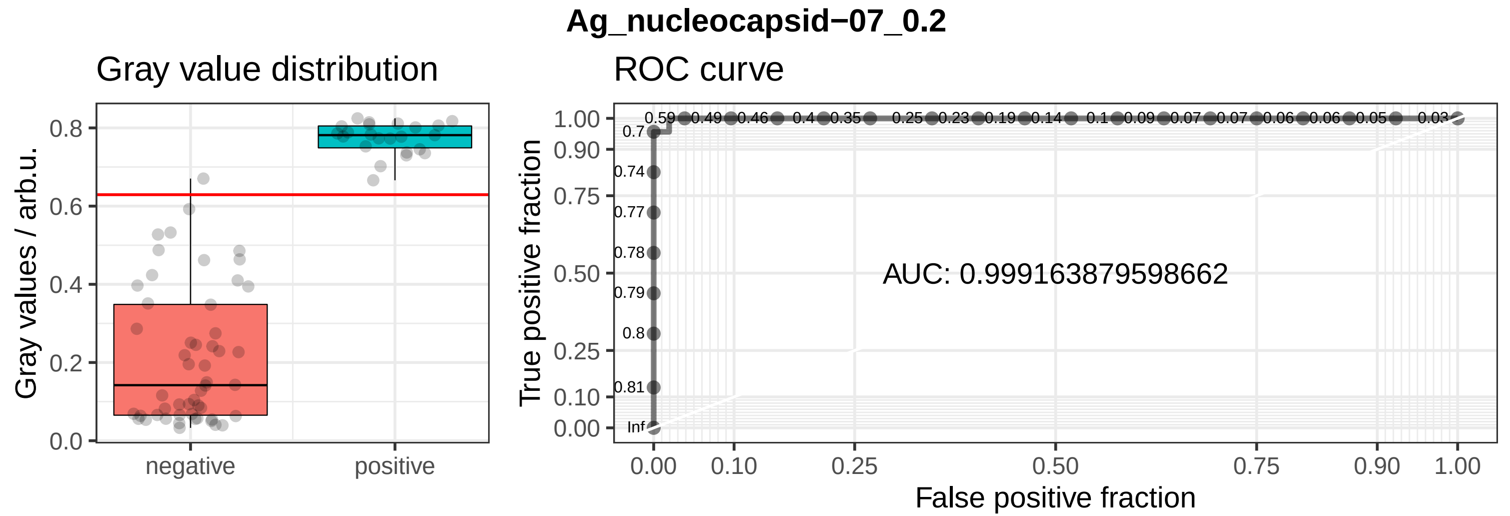

Ag_nucleocapsid−07_0.2
Gray value distribution
Gray values / arb.u.
0.0
0.2
0.4
0.6
0.8
negative
positive
ROC curve
True positive fraction
False positive fraction
0.00
0.10
0.25
0.50
0.75
0.90
1.00
AUC: 0.999163879598662
Inf
0.81
0.8
0.79
0.78
0.77
0.74
0.7
0.59
0.49
0.46
0.4
0.35
0.25
0.23
0.19
0.14
0.1
0.09
0.07
0.07
0.06
0.06
0.05
0.03

Ag_nucleocapsid−08_0.1

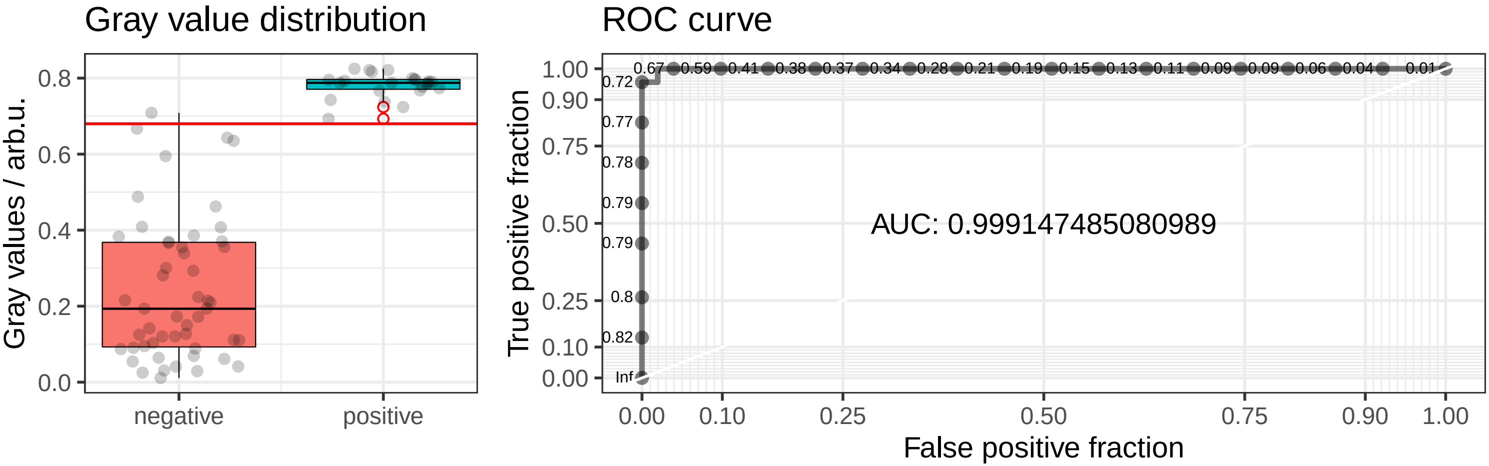

Ag_nucleocapsid−08_0.2

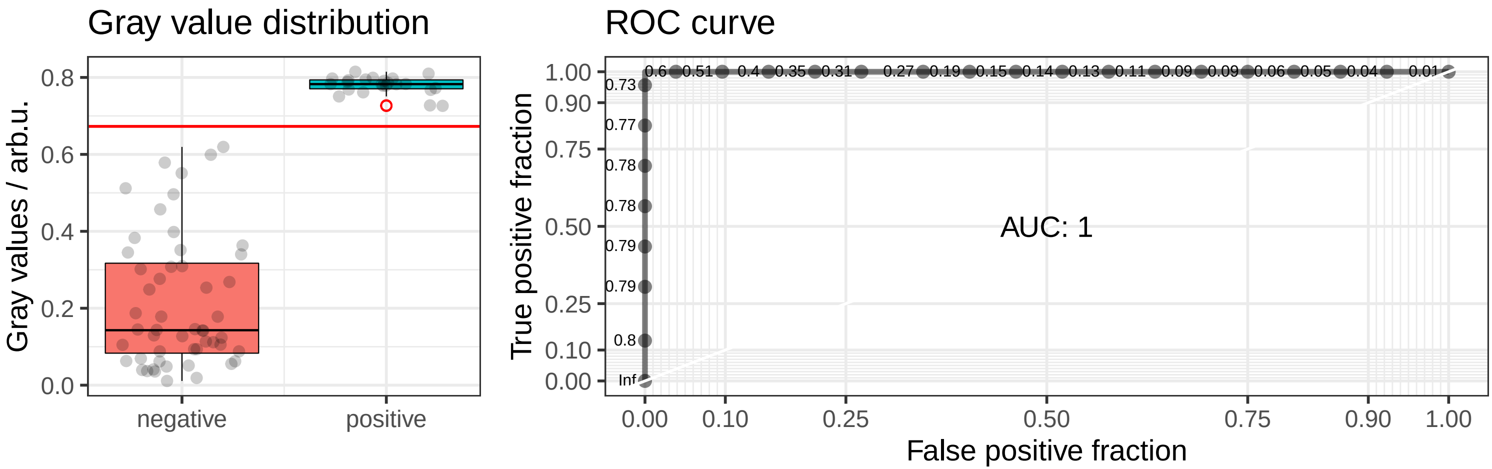

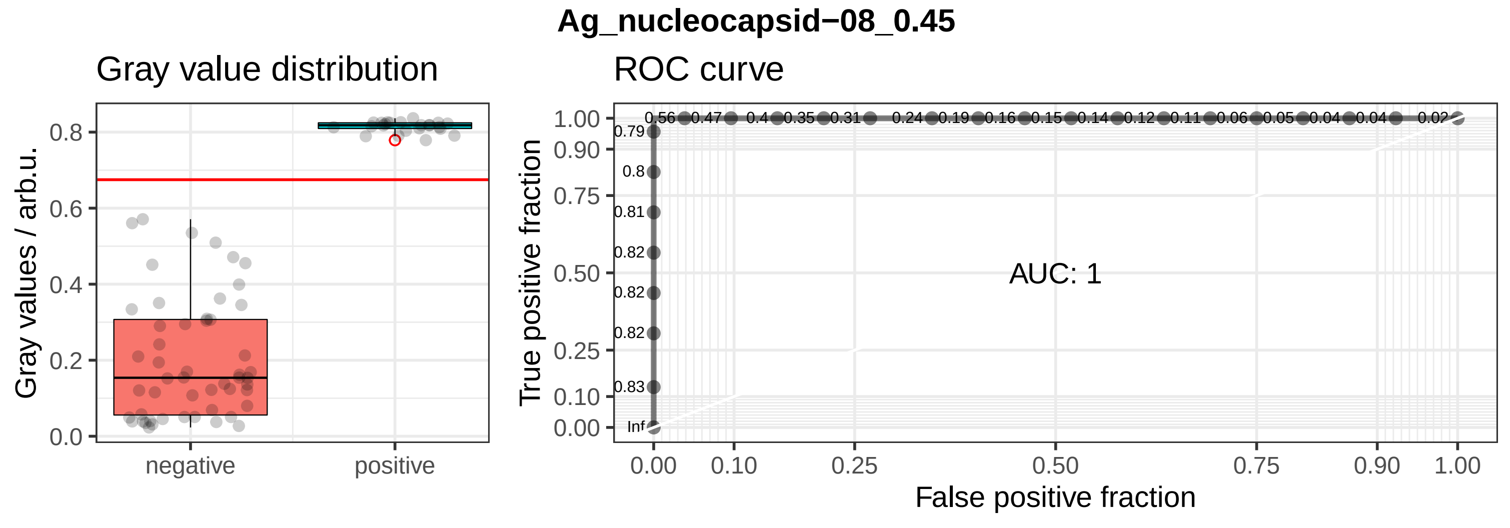

Ag_nucleocapsid−08_0.45
Gray value distribution
Gray values / arb.u.
0.0
0.2
0.4
0.6
0.8
negative
positive
ROC curve
True positive fraction
False positive fraction
0.00
0.10
0.25
0.50
0.75
0.90
1.00
AUC: 1
Inf
0.83
0.82
0.82
0.82
0.81
0.8
0.79
0.56
0.47
0.4
0.35
0.31
0.24
0.19
0.16
0.15
0.14
0.12
0.11
0.06
0.05
0.04
0.04
0.02

Ag_nucleocapsid−09_0.1

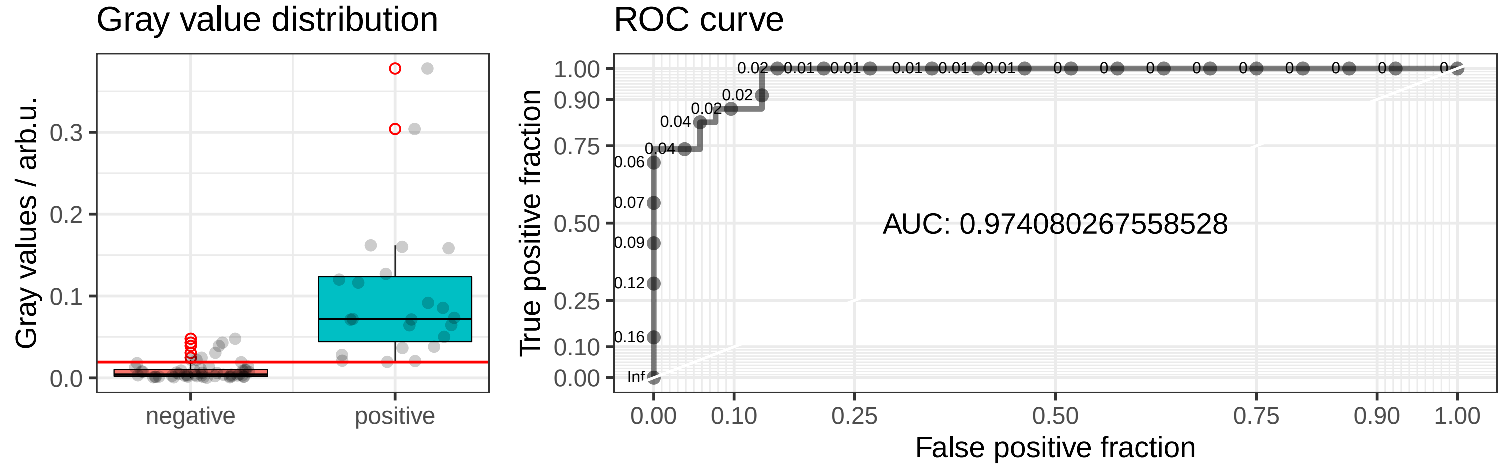

Ag_nucleocapsid−09_0.2

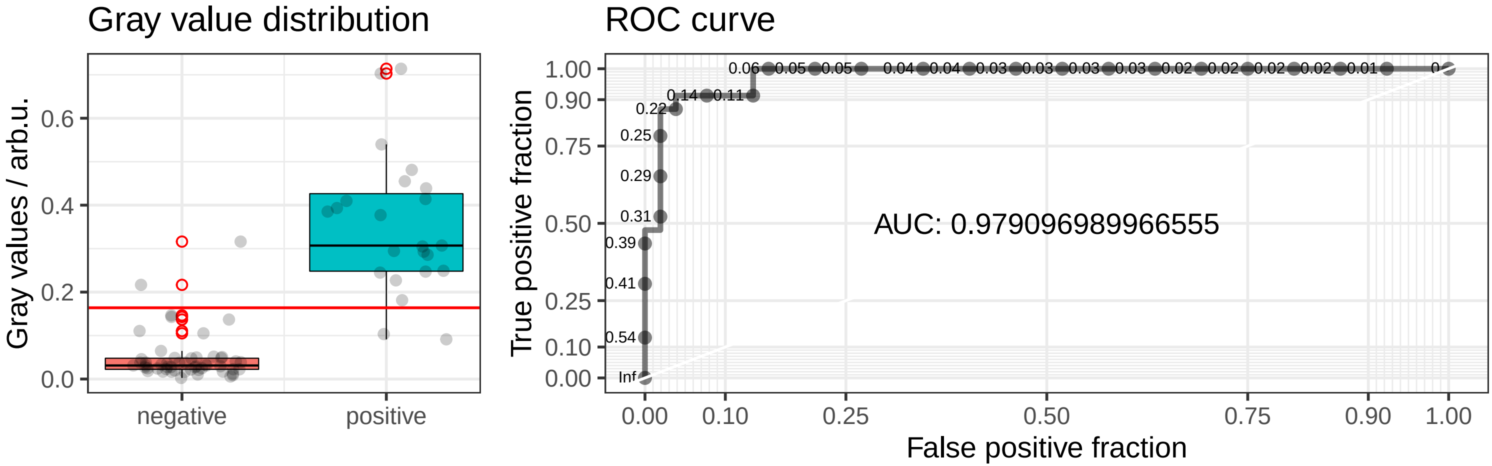

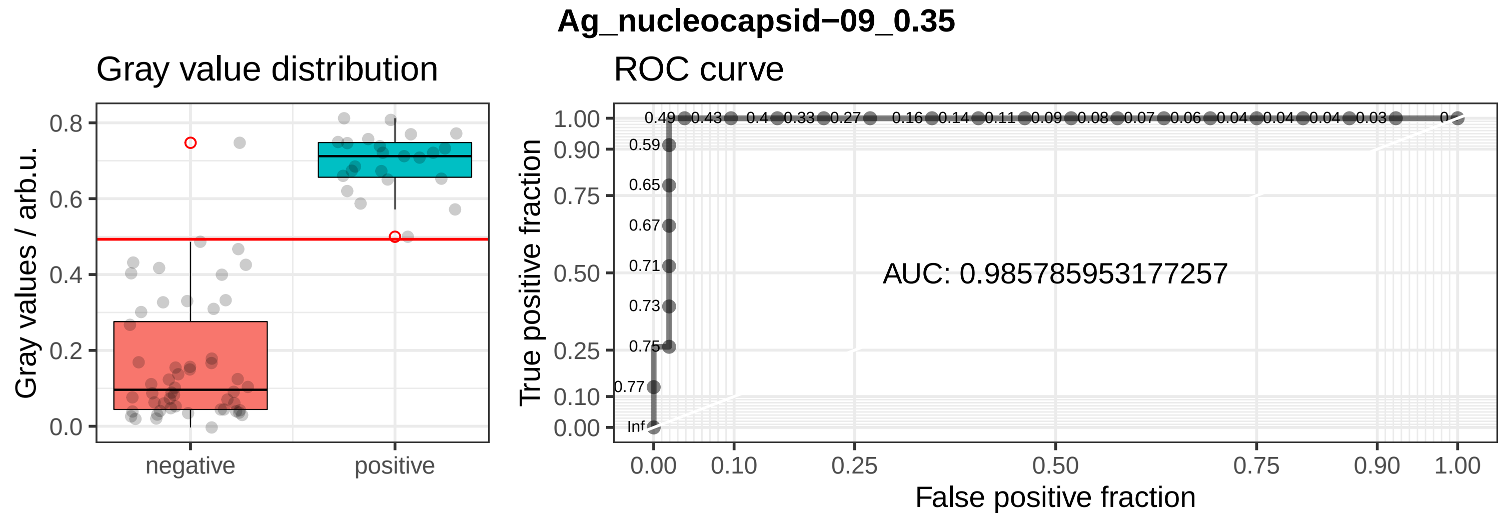

Ag_nucleocapsid−09_0.35
Gray value distribution
Gray values / arb.u.
0.8
0.6
0.4
0.2
0.0
negative
positive
ROC curve
True positive fraction
False positive fraction
1.00
0.90
0.75
0.50
0.25
0.10
0.00
AUC: 0.985785953177257
0.49
0.43
0.4
0.33
0.27
0.16
0.14
0.11
0.09
0.08
0.07
0.06
0.04
0.04
0.04
0.03
0.59
0.65
0.67
0.71
0.73
0.75
0.77
Inf

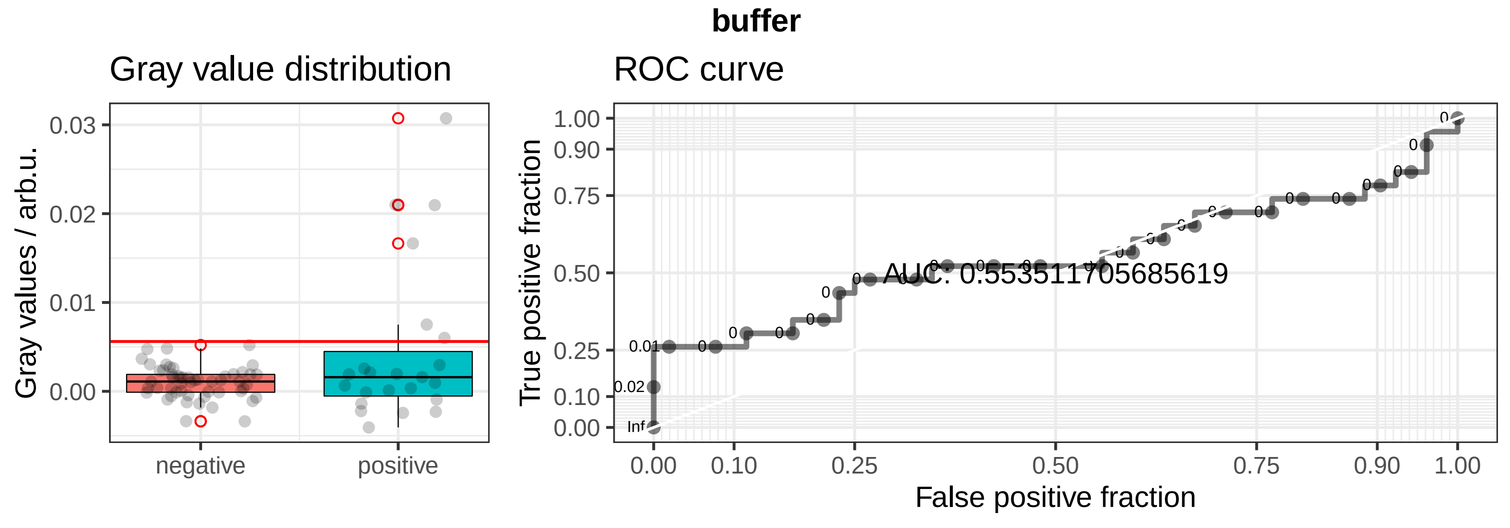

buffer
Gray value distribution
Gray values / arb.u.
0.03
0.02
0.01
0.00
negative
positive
ROC curve
True positive fraction
False positive fraction
1.00
0.90
0.75
0.50
0.25
0.10
0.00
0.00
0.10
0.25
0.50
0.75
0.90
1.00
AUC: 0.553511705685619
Inf
0.02
0.01

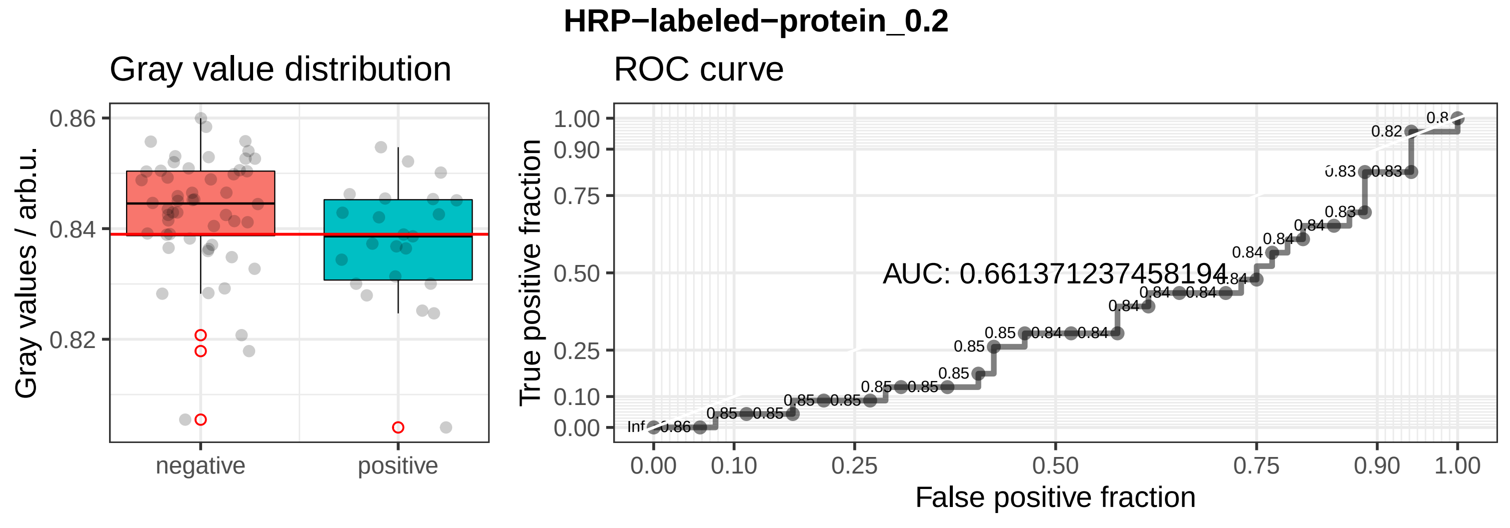
HRP−labeled−protein_0.2
Gray value distribution
Gray values / arb.u.
0.86
0.84
0.82
negative
positive
ROC curve
True positive fraction
False positive fraction
1.00
0.90
0.75
0.50
0.25
0.10
0.00
AUC: 0.661371237458194
