# Supplementary material for: Development of a new antigen-based microarray platform for screening and detection of human IgG antibodies against SARS-CoV-2
Source: Sci Rep. 2022 May 16;12:8067. doi: 10.1038/s41598-022-10823-7 (PMC9109672; doi:10.1038/s41598-022-10823-7)
Supplement: Supplementary file 1 — Supplementary Information. [file 41598_2022_10823_MOESM1_ESM.zip › Supplemental_File/Figure_S2.pdf]

Ag_Diphteria_Toxoid_VS_0.1

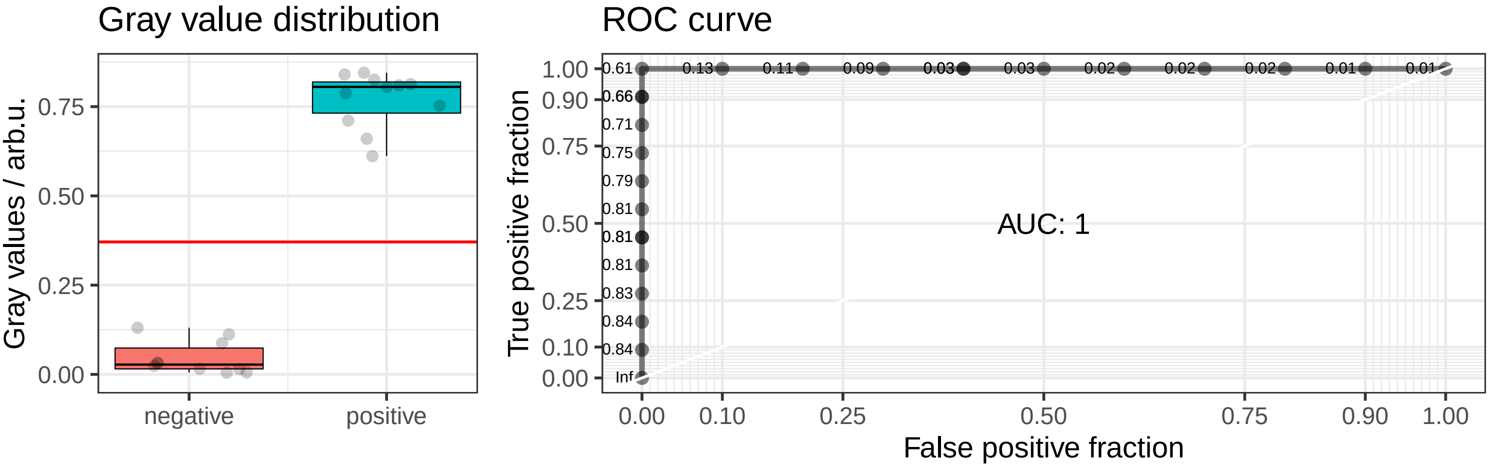

Ag_Diphteria_Toxoid_VS_0.5

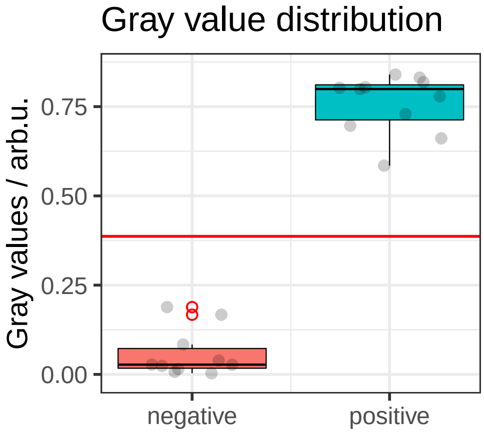

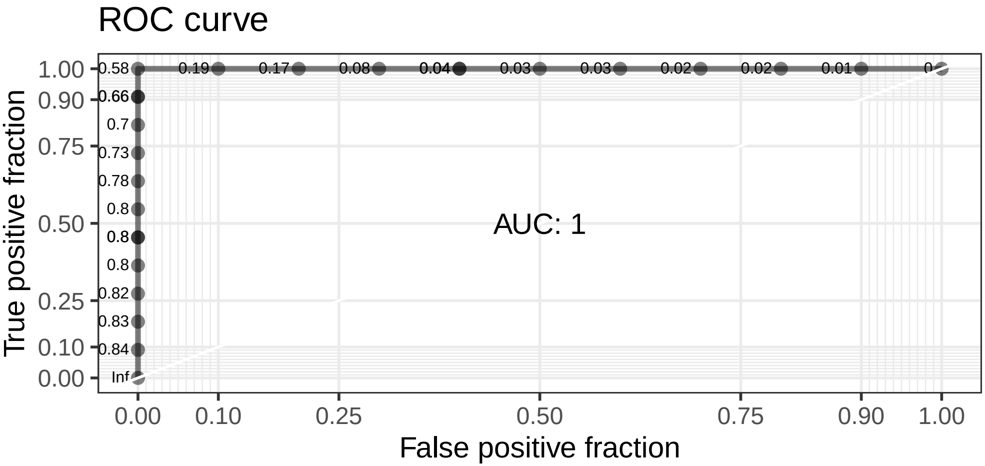

Ag_Masern_Virus_Premium_VS_0.2

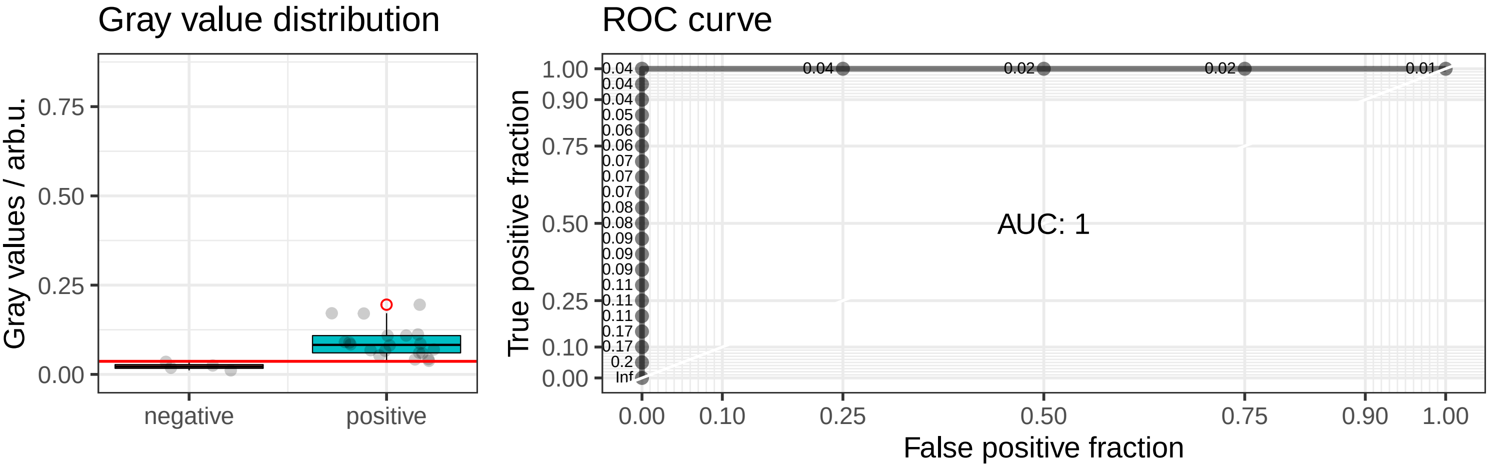

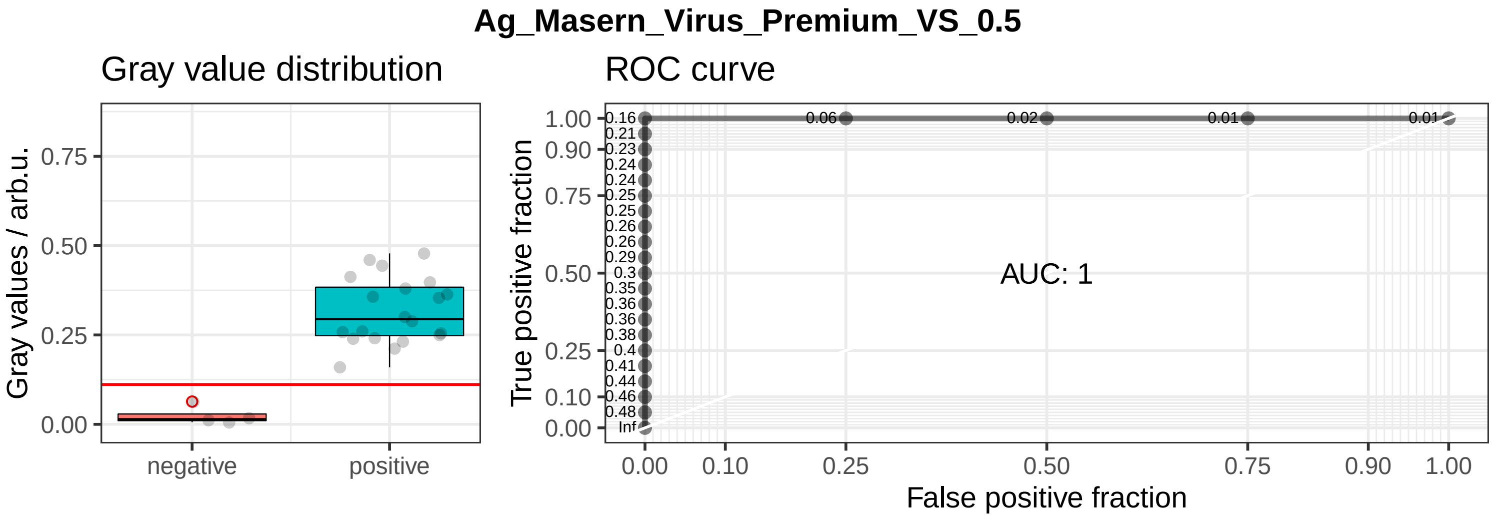

Ag_Masern_Virus_Premium_VS_0.5
Gray value distribution
Gray values / arb.u.
0.75
0.50
0.25
0.00
negative
positive
ROC curve
True positive fraction
1.00
0.90
0.75
0.50
0.25
0.10
0.00
0.16
0.21
0.23
0.24
0.24
0.25
0.25
0.26
0.26
0.29
0.3
0.35
0.36
0.36
0.38
0.4
0.41
0.44
0.46
0.48
Inf
0.06
0.02
0.01
0.01
AUC: 1
0.00
0.10
0.25
0.50
0.75
0.90
1.00
False positive fraction

Ag_Mumps_Virus_VS_0.2

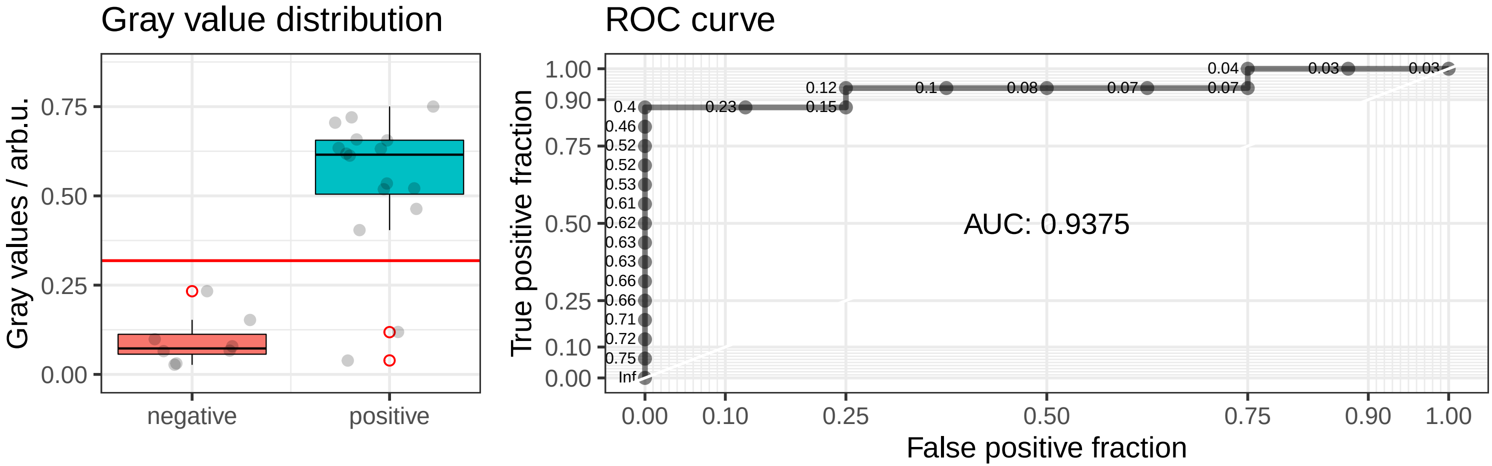

Ag_Mumps_Virus_VS_0.5

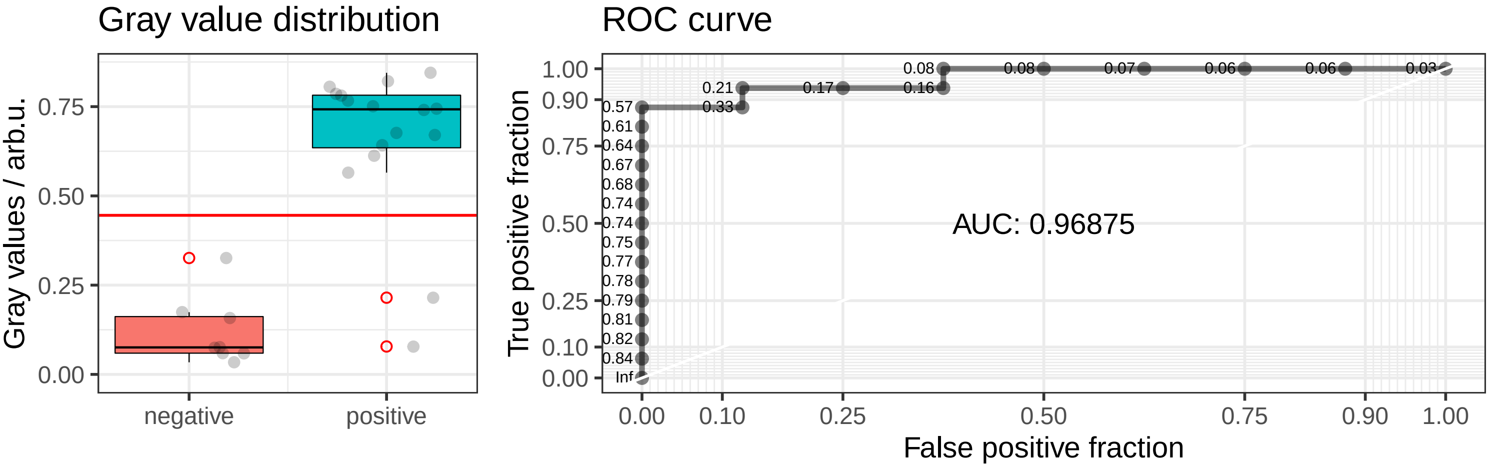

Ag_Tetanus_Toxid_VS_0.1

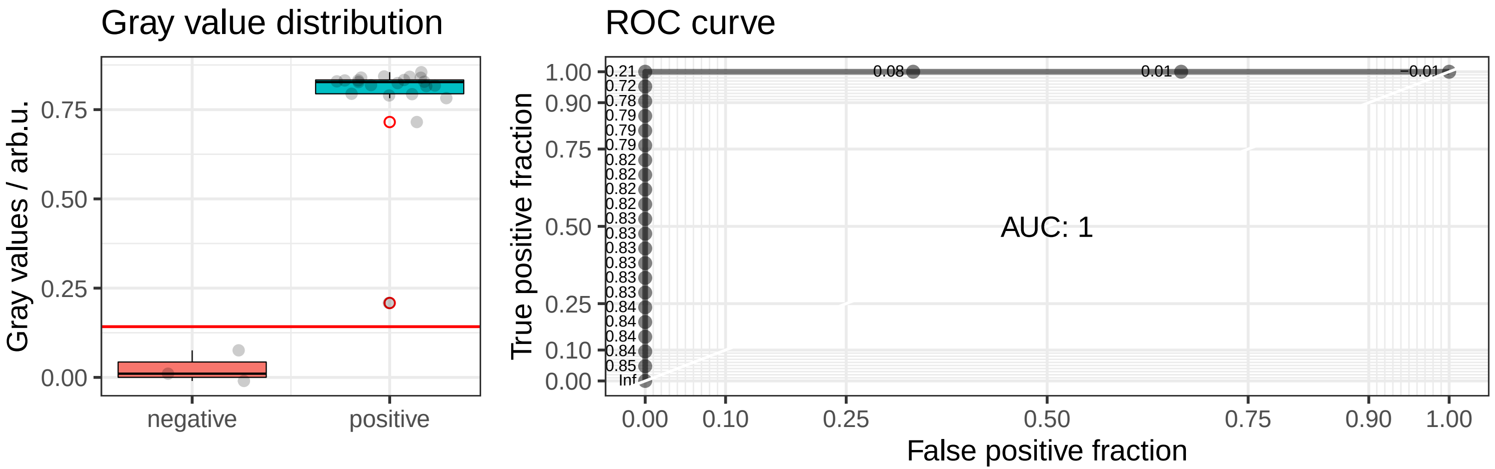

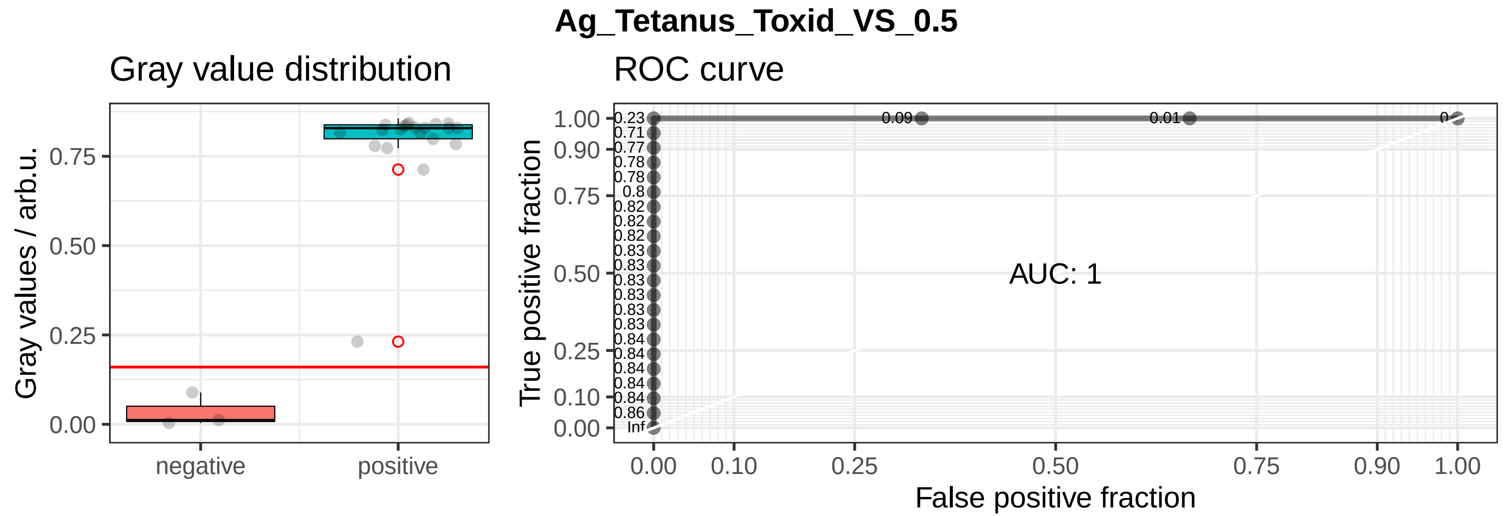

Ag_Tetanus_Toxid_VS_0.5
Gray value distribution
Gray values / arb.u.
0.75
0.50
0.25
0.00
negative
positive
ROC curve
True positive fraction
False positive fraction
1.00
0.90
0.75
0.50
0.25
0.10
0.00
0.10
0.25
0.50
0.75
0.90
1.00
AUC: 1
0.23
0.71
0.77
0.78
0.78
0.8
0.82
0.82
0.82
0.83
0.83
0.83
0.83
0.83
0.83
0.84
0.84
0.84
0.84
0.84
0.86
Inf
0.09
0.01
