# Supplementary material for: Development of a new antigen-based microarray platform for screening and detection of human IgG antibodies against SARS-CoV-2
Source: Sci Rep. 2022 May 16;12:8067. doi: 10.1038/s41598-022-10823-7 (PMC9109672; doi:10.1038/s41598-022-10823-7)
Supplement: Supplementary file 1 — Supplementary Information. [file 41598_2022_10823_MOESM1_ESM.zip › Supplemental_File/Supplementary_Information.docx]

# **Development of a new antigen-based microarray platform for screening and detection of human IgG antibodies against SARS-CoV-2**

Authors:

Sindy Burgold-Voigt^1,2*^, Elke Müller^1,2^, David Zopf^,1,3^, Stefan Monecke^1,2,4^, Sascha D. Braun^1,2^, Katrin Frankenfeld^5^, Michael Kiehntopf^6^, Sebastian Weis^7,8^ Thomas Schumacher^9^, Mathias W. Pletz^7^, Ralf Ehricht^1,2,3^ and the CoNAN study group^10^

Affiliations:

^1^Leibniz-Institute of Photonic Technology (Leibniz-IPHT), Jena, Germany.

^2^InfectoGnostics Research Campus, Centre for Applied Research, Jena, Germany.

^3^Friedrich Schiller University Jena, Institute of Physical Chemistry, Jena, Germany.

^4^Institute for Medical Microbiology and Virology, Dresden University Hospital, Dresden, Germany.

^5^INTER-ARRAY, Research Center for Medical Technology and Biotechnology (fzmb GmbH), Bad Langensalza, Germany.

^6^Institute for Clinical Chemistry and Laboratory Diagnostics and Integrated Biobank Jena (IBBJ), Jena University Hospital - Friedrich Schiller University, Jena, Germany.

^7^Institute for Infectious Diseases and Infection Control, Jena University Hospital - Friedrich Schiller University, Jena, Germany.

^8^Leibnitz-Institute for Infection Biology and Natural Product Research-Hans Knöll Institute - HKI, Jena, Germany.

^9^Institut Virion\Serion GmbH, Würzburg, Germany.

## ^10^Informations can be found in Supplemental File S9.

## Supplementary Information

**Supplemental Files S1** and **S2** are provided as single .pdf-files.

In **Supplemental File S1** each individual receiver operating characteristic curves (ROC) and grey value distribution for each spotted concentration of 18 different SARS-CoV-2 antigen preparations are listed.

**Supplemental File S2** contains each individual receiver operating characteristic curves (ROC) and grey value distribution for different concentrations of the additional vaccination antigens.

**Supplemental Figures S3 – S6** are listed below. All figures show the heat map of the raw array data of each study, described in the paper, before threshold values were applied.

**Supplemental Files S8** and **S9** are provided as single files.


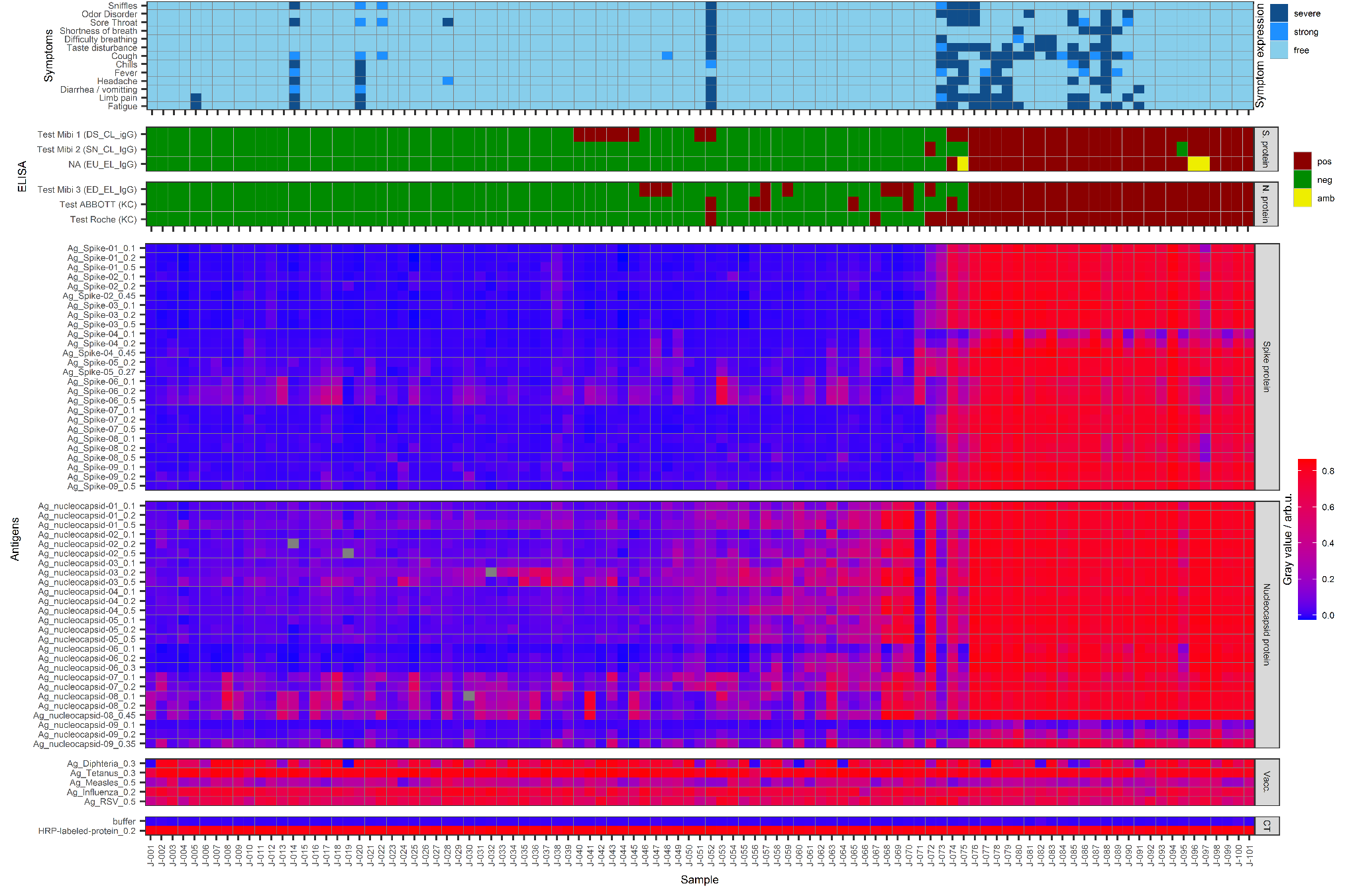


**Figure S3:** Raw data for CoNAN samples before applying threshold values.


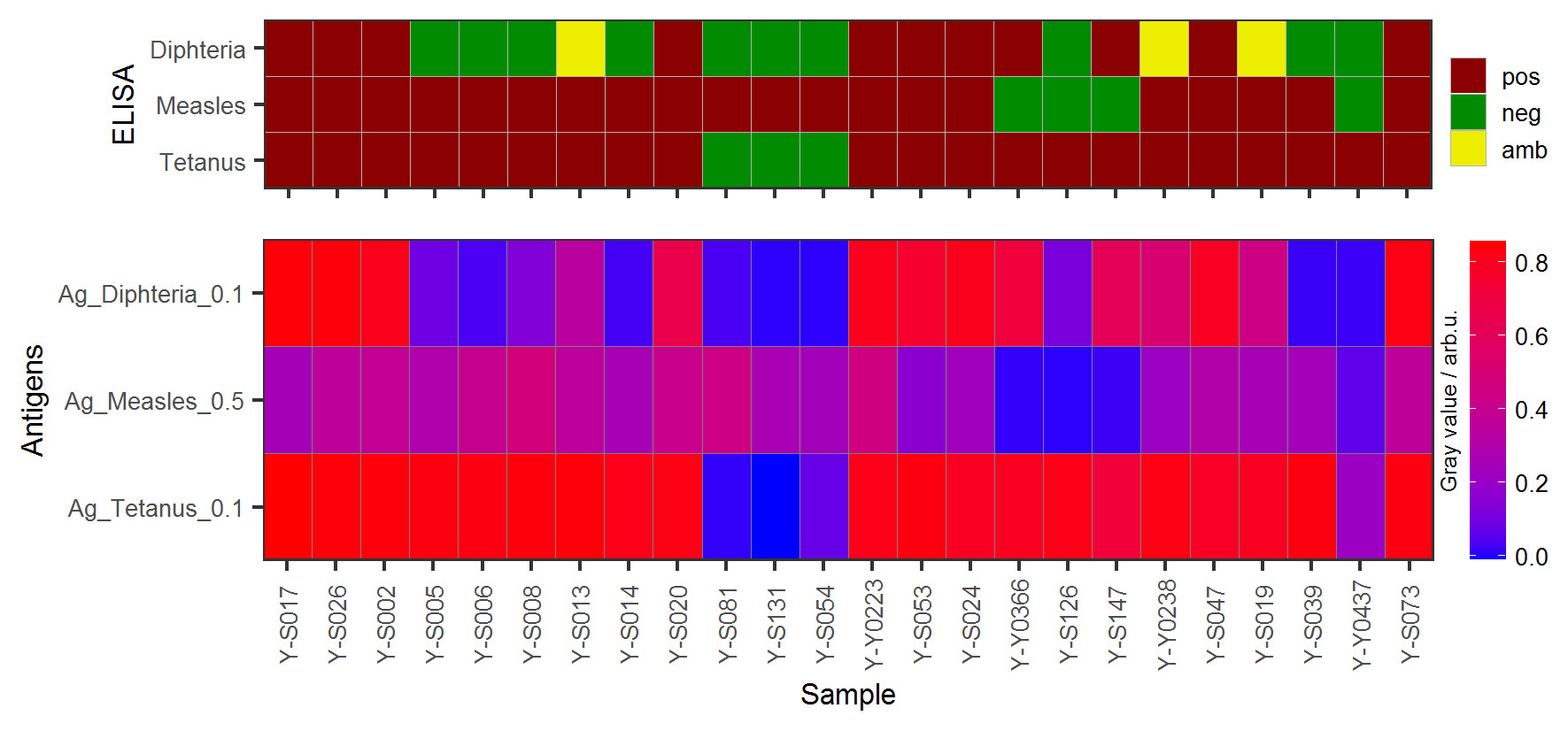


**Figure S4:** Raw data for additional vaccination antigens on microarray before applying threshold values.


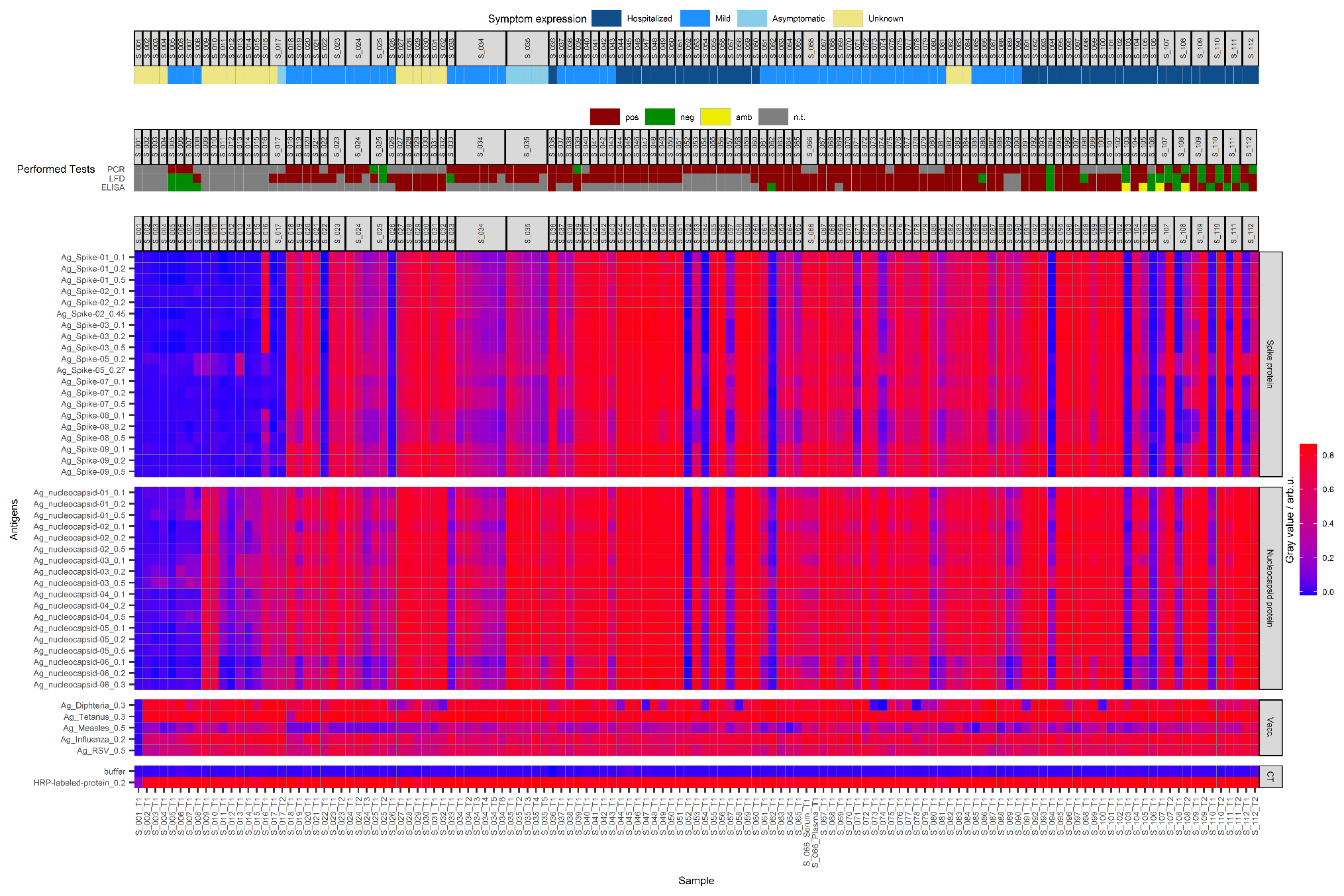


**Figure S5:** Raw data for 131 tested sera on new microarray before applying threshold values.


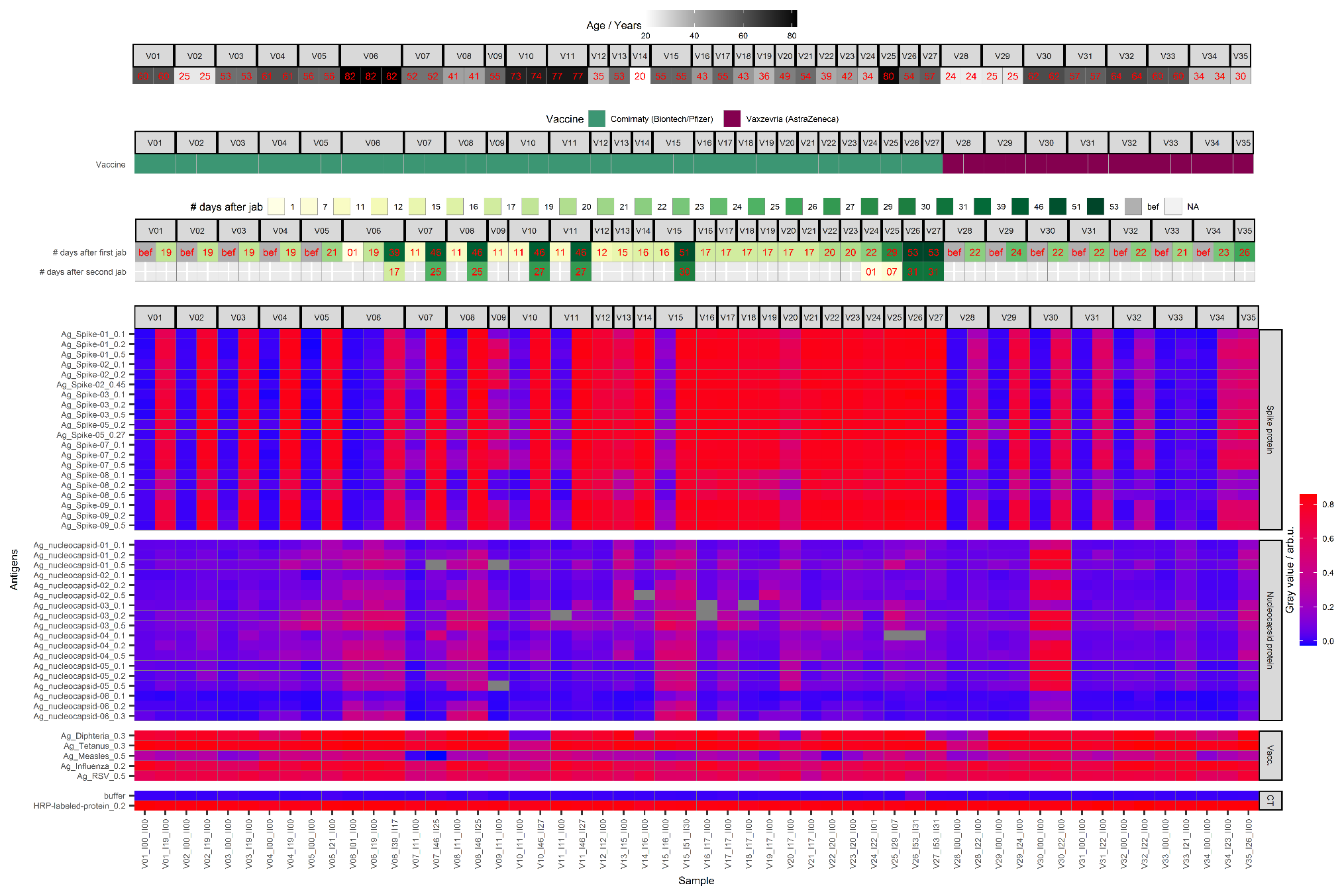


**Figure S6:** Raw data for vaccination sera before applying threshold values.

The complete layout of the SARS-CoV2-VAC microarray used in the study is shown in **Figure S7**. The substances and concentrations of the individual spots are listed below.


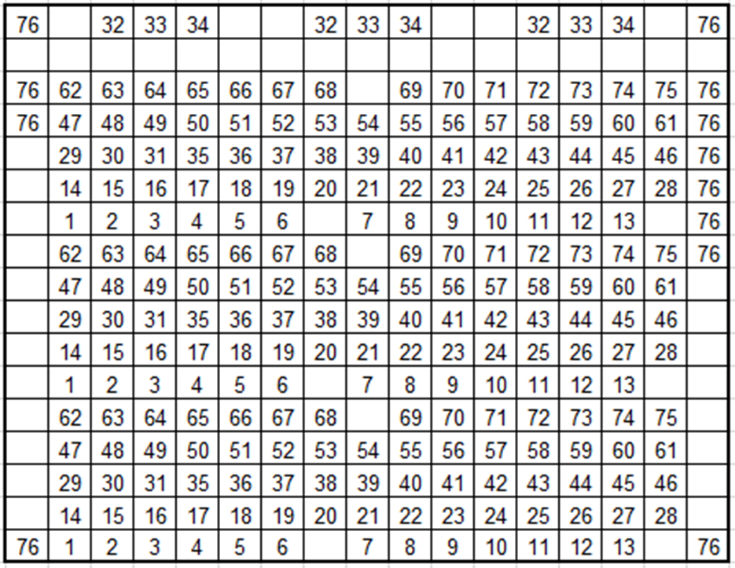

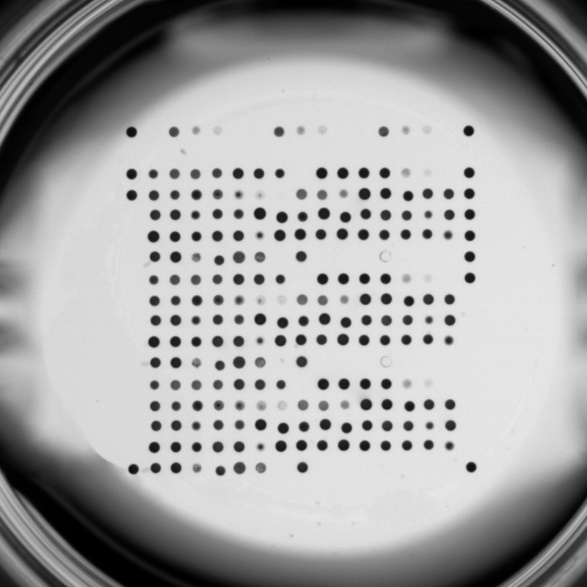


**Figure S7:** Layout of the produces new SARS-CoV2-VAC antigen microarray chip. Antigen were spotted in the listed concentration in brackets (µg / µL):

**1**: Diphtheria Toxoid (0.3)

**2**: Tetanus Toxoid (0.3)

**3**: Measles Virus (0.5)

**4**: Mumps Virus (0.5)

**5**: Influenza A Virus (0.2)

**6**: Human Orthopneumovirus (RSV) (0.5)

**7:** Human Polio Virus (HPV) (0.5)

**8-13:** Chlamydia trachomatis Peptides

**14-16**: Nucleocapsid Protein, Diagreat (**14** = 0.45, **15** = 0.2, **16** = 0.1)

**17-19**: Spike Protein (RBD), Diagreat (**17** = 0.5, **18** = 0.2, **19** = 0.1)

**20-22**: Nucleocapsid Protein, Virion\Serion (**20** = 0.5, **21** = 0.2, **22** = 0.1)

**23-25**: Nucleocapsid Protein, re-buffered, Virion-Serion (**23** = 0.5, **24** = 0.2, **25** = 0.1)

**26-28**: Nucleocapsid Protein, re-buffered,

Group of Prof. Aguzzi (**26** = 0.3, **27** = 0.2, **28** = 0.1)

**29-31**: Spike Protein (RBD), Sino Biological (**29** = 0.2, **30** = 0.3 and **31** = 0.2, re-buffered)

**32-34**: Spike Protein, Virion\Serion (**32** = 0.45, **33** = 0.2, **34** = 0.1)

**35-36**: Spike Protein, re-buffed, Virion\Serion (**35** = 0.27, **36** = 0.2)

**37-39**: Nucleocapsid Protein, Biomapper (**37** = 0.5, **38** = 0.2, **39** = 0.1)

**40-42**: Nucleocapsid Protein, re-buffered, Biomapper (**40** = 0.5, **41** = 0.2, **42** = 0.1)

**43-45**: Spike Protein, Biomapper (**43** = 0.5, **44** = 0.2, **45** = 0.1)

**46-48**: Spike Protein, re-buffered, Biomapper (**46** = 0.45, **47** = 0.2, **48** = 0.1)

**49-50**: Nucleocapsid Protein, re-buffered, BioVendor (**49** = 0.2, **50** = 0.1)

**51-53**: Nucleocapsid Protein, GeneTex (**51** = 0.35, **52** = 0.2, **53** = 0.1)

**54-56**: Spike Protein (S1), rebuffered, Sino Biological (**54** = 0.45, **55** = 0.2, **56** = 0.1)

**57-59**: Nucleocapsid Protein, Medix (**57** = 0.5, **58** = 0.2, **59** = 0.1)

**60-62**: Spike Protein (S1), Medix (**60** = 0.5, **61** = 0.2, **62** = 0.1)

**63-65**: Spike Protein (RBD 1), Medix (**63** = 0.5, **64** = 0.2, **65** = 0.1)

**66-68**: Spike Protein (RBD 2), Medix (**66** = 0.5, **67** = 0.2, **68** = 0.1)

**69-72**: human IgG (**69** = 0.2, **70** = 0.15, **71** = 0.1, **72** = 0.05)

**73-74**: human IgM (**73** = 0.2, **74** = 0.1)

**75**: buffer

**76**: recombinant protein A/G-HRP

**Supplemental File 8** is provided as a separate .xlsx -file which is divided into four spreadsheets containing the microarray raw data of:

a.) CoNAN verification study

b.) verification study of additional vaccination antigens

c.) tested sera for application of new microarray and

d.) tested vaccination sera.

On all spreadsheets, the first column contains the spotted substance names and concentrations. The first row contains the sample IDs.

Relative signal intensities of defined regions (at predefined spot coordinates) on the microarray were determined per sample for each substance. The normalized intensities (NI) of the spots were determined as NI = 1-(M/BG), where M is the average intensity of the spot and BG is the intensity of the local background. Thus, the NI values ranged between 0 (no signal) to 1 (maximum intensity).

**Supplemental File 9** contains the members (names and affiliations) of the CoNAN study group.
